# Novel Indole-Based Sulfonylhydrazones as Potential Anti-Breast Cancer Agents: Synthesis, In Vitro Evaluation, ADME, and QSAR Studies

**DOI:** 10.3390/ph18081231

**Published:** 2025-08-20

**Authors:** Violina T. Angelova, Rositsa Mihaylova, Zvetanka Zhivkova, Nikolay Vassilev, Boris Shivachev, Irini Doytchinova

**Affiliations:** 1 Faculty of Pharmacy, Medical University of Sofia, 1000 Sofia, Bulgaria; rmihaylova@pharmfac.mu-sofia.bg (R.M.); zzhikova@pharmfac.mu-sofia.bg (Z.Z.); idoytchinova@pharmfac.mu-sofia.bg (I.D.); 2 Laboratory “Nuclear Magnetic Resonance”, Institute of Organic Chemistry with Centre of Phytochemistry, Bulgarian Academy of Sciences, 1113 Sofia, Bulgaria; nikolay.vassilev@orgchm.bas.bg; 3 Institute of Mineralogy and Crystallography “Acad. Ivan Kostov”, Bulgarian Academy of Sciences, 1113 Sofia, Bulgaria; blshivachev@gmail.com

**Keywords:** indole, sulfonylhydrazones, MCF-7, MDA-MB-231, breast cancer, anticancer activity, QSAR, X-ray

## Abstract

**Background**: Breast cancer continues to pose a significant global health challenge despite advances in early detection and targeted therapies. The development of novel chemotherapeutic agents remains crucial, particularly those with selective cytotoxicity toward specific breast cancer subtypes. **Methods**: A series of ten hybrid indolyl-methylidene phenylsulfonylhydrazones and one bis-indole derivative were designed, synthesized, and structurally characterized using NMR and high-resolution mass spectrometry (HRMS). Prior to synthesis, in silico screening was performed to assess drug likeness and ADME-related properties. Single-crystal X-ray diffraction was conducted for compound **3e**. The cytotoxic potential of the synthesized compounds was evaluated using the MTT assay against MCF-7 (ER-α⁺) and MDA-MB-231 (triple-negative) breast cancer cell lines. Additionally, quantitative structure–activity relationship (QSAR) analysis was conducted to identify key structural features contributing to activity. **Results**: Most compounds exhibited selective cytotoxicity against MCF-7 cells. Notably, compound **3b** demonstrated the highest potency with an IC_50_ of 4.0 μM and a selectivity index (SI) of 20.975. Compound **3f** showed strong activity against MDA-MB-231 cells (IC_50_ = 4.7 μM). QSAR analysis revealed that the presence of a non-substituted phenyl ring and specific indolyl substituents (5-methoxy, 1-acetyl, 5-chloro) significantly contributed to enhanced cytotoxic activity and ligand efficiency. **Conclusion**: The synthesized phenylsulfonylhydrazone hybrids exhibit promising and selective cytotoxicity, particularly against ER-α⁺ breast cancer cells. Structural insights from QSAR analysis provide a valuable foundation for the further optimization of this scaffold as a potential source of selective anticancer agents.

## 1. Introduction

Breast cancer (BC) remains a major global health challenge, with survival rates varying considerably due to the heterogeneous nature of the disease and differences in diagnostic and therapeutic strategies [1]. Prognosis is largely determined by tumor biology, disease stage at diagnosis, and access to effective treatment options. Despite notable progress in targeted therapies, including immunotherapy and antibody-drug conjugates such as sacituzumab govitecan (Trodelvy) and trastuzumab deruxtecan (Enhertu) [2], drug resistance and treatment-related toxicity continue to limit therapeutic efficacy, especially in advanced and metastatic cases [3,4]. Accordingly, the development of novel anticancer agents, particularly small molecules with improved efficacy and selectivity, remains a central research focus [5,6,7,8,9,10,11,12,13,14,15,16]. High-throughput screening and molecular targeting technologies have accelerated the discovery of various therapeutic classes, including small molecules, monoclonal antibodies, and immune checkpoint inhibitors [17,18,19,20,21].

Sulfonylhydrazones are regarded as excellent pharmacophores in drug design due to their versatile chemical and biological properties [22,23]. They demonstrate a broad spectrum of pharmacological activities, including antibacterial [24,25,26], antifungal [27], and anti-inflammatory [28] effects. Additionally, sulfonylhydrazones have been found to possess neuroprotective properties attributed to their antioxidant [29,30,31] and anticholinesterase activities [32,33,34,35]. Recent studies have pointed out their potency in inhibiting tumor growth through several mechanisms [36,37,38,39]. The sulfonylhydrazone scaffold is highly interactive, capable of acting as both hydrogen bond donor and acceptor. In addition, some essential properties such as lipophilicity, water solubility, and electronic characteristics could be modulated by different substituents [23,40,41,42].

Notably, the indole core is a privileged structure in drug discovery, present in numerous bioactive compounds such as serotonin, melatonin, and indomethacin. Its ability to interact with enzyme active sites and receptors through hydrogen bonding and π–π stacking makes it highly valuable in medicinal chemistry. Importantly, hybrids combining indole and sulfonylhydrazones offer exceptional versatility, capable of interacting with diverse biological targets and exhibiting antioxidant, anti-inflammatory, and enzyme-inhibitory properties [32,43,44]. Their potential in cancer therapy is particularly promising, as they operate through a dual mechanism of action: DNA intercalation (via the indole moiety) and enzyme inhibition (via the sulfonylhydrazone group) [36,37,38,45,46,47]. Such a combination offers opportunities for synergistic effects and enhanced therapeutic profiles.

Previously, we applied quantitative structure–activity relationship (QSAR) analysis to a series of arylsulfonylhydrazones, identifying key structural determinants of anticancer activity against estrogen receptor-positive (ER+) MCF-7 and triple-negative breast cancer (TNBC) MDA-MB-231 cell lines [48]. Based on these models, we designed and synthesized new derivatives, many of which displayed potent in vitro cytotoxicity, particularly against MCF-7 cells. Several compounds exhibited IC_50_ values below 1 μM, with the most active derivatives featuring 5-Cl, 5-OCH_3_, or 1-COCH_3_ substituents on the indole rings as critical contributors to enhanced activity.

In this study, we expanded upon our previous findings [48] by designing and synthesizing new indole-sulfonylhydrazone hybrids to identify compounds with enhanced anticancer activity. Our workflow combined in silico drug likeness and ADME profiling with targeted synthesis and in vitro cytotoxicity evaluation against MCF-7 and MDA-MB-231 cells. Based on prior SAR insights, we prioritized 5-Cl, 1-COCH_3_, and 5-Br substitutions to refine biological activity and selectivity. Additionally, we investigated a bis-indole hydrazone derivative lacking the sulfonyl group, motivated by preliminary evidence [49] suggesting superior anticancer potential of such analogues.

## 2. Results

### 2.1. Design of Novel Indolyl-methylidene-Substituted Phenylsulfonylhydrazones

Building on our previous QSAR analysis and in vitro cytotoxicity evaluation of arylsulfonylhydrazones as anticancer agents [48], we designed a series of ten hybrid indole derivatives (compounds **3a–j**) incorporating the phenylsulfonylhydrazone fragment (–C=N–NH–SO_2_–Ar), along with one symmetrical bis-indole hydrazone derivative (compound **4**). These hybrids combine two key pharmacophores—indole and sulfonylhydrazone—and were subjected to in silico screening to evaluate drug likeness, pharmacokinetic properties, and in vitro anticancer activity against two breast cancer cell lines: MCF-7 (ER-α⁺) and MDA-MB-231 (triple-negative). The Ar substituents included phenyl, 4-methylphenyl, 4-methoxyphenyl, and various dimethylphenyl groups, while the indole moiety featured substitutions such as 1-acetyl, 5-bromo, or 5-chloro. Additionally, compound **4** was designed as a symmetrical molecule bearing identical 5-chloro-indole substituents on both sides of the hydrazone (–C=N–N=C–) linker (Table 1).

### 2.2. In Silico Screening of the Designed Compounds for Drug Likeness

Before synthesis, the designed structures were evaluated in silico for drug likeness, considering their physicochemical properties, ADME characteristics, and pharmacokinetic (PK) parameters.

#### 2.2.1. Physicochemical Properties

The primary physicochemical properties calculated for the newly designed indole derivatives (compounds **3a–j**) containing the phenylsulfonylhydrazone fragment (–C=N–NH–SO_2_–Ar) and the symmetrical bis-indole hydrazone derivative (**4**) are summarized in Table 2. These include molecular weight (Mw), pKa values for acidic (A) and basic (B) groups, fractions of ionized species (fA and fB), logP, distribution coefficient at pH 7.4 (logD_7_._4_), polar surface area (PSA), number of free rotatable bonds (FRB), hydrogen bond donors (HBD), and hydrogen bond acceptors (HBA).

The molecular weights of the compounds (355–420 g/mol) fall within the threshold defined by Lipinski’s rule of five [49]. The compounds exhibit pKa values between 8.87 and 9.32, suggesting weakly ionizable functional groups. In contrast, the symmetrical molecule **4** acts as a weak base, with a pKa of 6.28, likely due to the protonation of hydrazone nitrogen atoms (–C=N–N=C–) and potential stabilization effects from molecular symmetry and internal interactions. At physiological pH (7.4), the neutral forms of all compounds predominate, as reflected by the negligible fractions of ionized species (fA and fB) and the similar values of logP and logD_7_._4_. All logP values are below 5, consistent with Lipinski’s rule. The PSA values range from 82 to 98 Å^2^, indicating good potential for oral absorption, though most compounds are unlikely to cross the blood–brain barrier (BBB), with the exception of compound **4**, which shows BBB permeability.

The number of free rotatable bonds ranges from 3 to 4; however, the Ph–S–N–N= segment exhibits limited flexibility due to p–π conjugation, imparting partial rigidity. The numbers of hydrogen bond donors and acceptors also comply with Lipinski’s criteria. Overall, all compounds satisfy the four main Lipinski’s rules for drug likeness.

#### 2.2.2. ADME Properties

The ADME properties calculated for the newly designed compounds are summarized in Table 3. Water solubility was estimated using three methods, with the results expressed as logS (logarithm of molar solubility). According to the solubility classification by Daina et al. (2017) [50], compounds with logS values between −10 and −6 are considered poorly soluble, while those between −6 and −4 are categorized as moderately soluble. Most of the compounds fall within the moderately soluble range, except for compounds **3d**, **3h**, and **4**, which are classified as poorly soluble.

**Figure 1:**
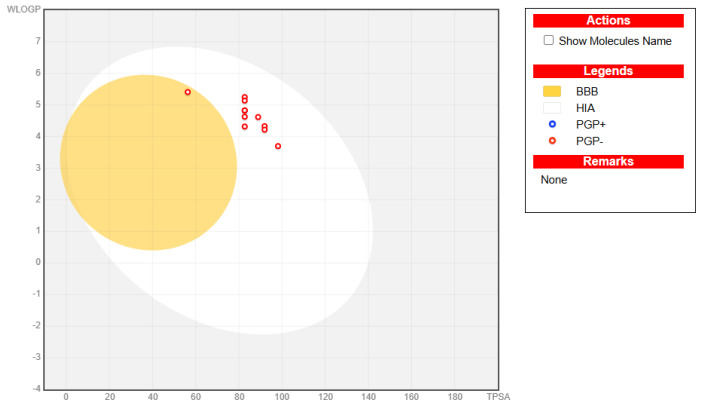
BOILED-Egg diagram for the designed indole derivatives (**3a–j**) and phenylsulfonylhydrazone fragment (-C=N-NH-SO_2_-Ar) and 3,3′-[(1*E*,2*E*)-hydrazine-1,2-diylidenedi(*E*)methylylidene]bis(5-chloro-1*H*-indole) (**4**). The presence in the white area corresponds to good oral permeability, while the presence in the yellow area corresponds to BBB permeability. The red circles indicate that the compounds are not substrates of P-gp.

The BOILED-Egg diagram [51] (Figure 1) indicates that all compounds exhibit high gastrointestinal absorption, and compound **4** is predicted to cross the blood–brain barrier (BBB). The bioavailability radar summarizes six key physicochemical parameters that define the optimal space for oral bioavailability [50]: lipophilicity (logP), molecular size (Mw), polarity (PSA), water solubility (logS), unsaturation (fraction of Csp^3^ atoms), and flexibility (number of rotatable bonds). The designed compounds meet most of these criteria but violate the unsaturation threshold (fraction of Csp^3^ atoms < 0.25), which was anticipated due to the predominance of sp^2^-hybridized carbon atoms in their structures. The bioavailability score suggests a 55% probability of achieving oral bioavailability greater than 10% in rats [52].

None of the compounds are predicted to be substrates of the P-glycoprotein (P-gp) transporter. In terms of cytochrome P450 (CYP) inhibition, each compound is predicted to inhibit between two and four of the five major CYP enzymes involved in drug metabolism (CYP1A2, CYP2C19, CYP2C9, CYP2D6, and CYP3A4). In addition to Lipinski’s rule compliance, all compounds also satisfy drug-likeness filters according to the Ghose [53], Veber [54], Egan [55], and Muegge [56].

Finally, the synthetic feasibility of the designed compounds was evaluated using the synthetic accessibility score, which ranges from 1 (very easy synthesis) to 10 (very difficult synthesis). The scores for the compounds, ranging from 2.65 to 3.07, indicate relatively easy synthesis, consistent with our previous experience [48,57].

#### 2.2.3. Pharmacokinetic (PK) Parameters

The key pharmacokinetic parameters—the fraction of the unbound to plasma proteins molecules (*fu*), total clearance (*CL*), steady-state volume of distribution (*VDss*), and half-life (t_1/2_)—for the newly designed indole derivatives (**3a–j**) with phenylsulfonylhydrazone fragment (–C=N–NH–SO_2_–Ar) and 3,3′-[(1*E*,2*E*)-hydrazine-1,2-diylidenedi(*E*)methylylidene]bis(5-chloro-1*H*- indole) (**4**) were calculated using QSPkR models for neutral molecules previously developed in our lab [57,58,59]. The predicted values are presented in Table 4.

All compounds showed extensive plasma protein binding, with *fu* values ranging from 0.002 to 0.022. This high protein binding is primarily attributed to their high lipophilicity, as logP is a major determinant. Neutral compounds are known to bind to major plasma proteins such as human serum albumin, alpha-1-acid glycoprotein, and lipoproteins [59]. The predicted *CL* values range from 0.166 to 0.504 L/h/kg, falling within the range reported in a study analyzing 754 drugs with varying ionization states. That study found that 45% of neutral drugs exhibit low *CL* (<0.24 L/h/kg), 16% show high *CL* (>0.96 L/h/kg), and 39% fall within a moderate range [60]. The compounds display moderate *VDss* values between 0.733 and 2.730 L/kg. As the *VDss* values exceed total body water volume, this suggests moderate tissue distribution without significant accumulation. The highest *VDss* value was observed for compound **4**, likely due to its slight ionization as a base. This observation is consistent with the general trend that basic compounds tend to have higher *VDss* values than acids or neutral molecules. The predicted half-lives (t_1/2_) of the compounds vary between 1.25 and 5.07 h.

### 2.3. Synthesis of the Novel Indolyl-methylidene Substituted Phenylsulfonylhydrazones **3a–j** and Bis(indolyl methylidene)diazine Derivative **4**

**Scheme 1:**
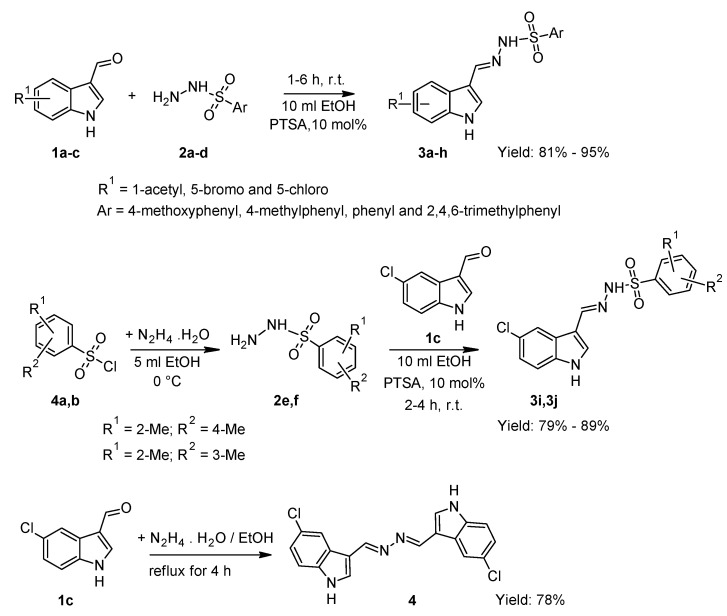
Synthesis of indole derivatives (**3a–j**) with phenylsulfonylhydrazone fragment and 1,2-bis-indole hydrazine derivative (**4**).

The designed compounds demonstrated strong drug likeness in in silico screening, leading us to proceed with their synthesis and biological evaluation. The synthetic routes for the indole derivatives (**3a–j**) containing the phenylsulfonyl hydrazone fragment (–C=N–NH–SO_2_–Ar) and the 1,2-bis-indole hydrazine derivative (**4**) are presented in Scheme 1.

The synthetic pathway for the indole derivatives is outlined in Scheme 1. Compounds **3a–h** were synthesized via a one-step reaction, while compounds **3i** and **3j** required two steps. Initially, 1- or 5-substituted indole derivatives (**3a–h**), bearing various phenylsulfonyl-hydrazone fragments at the 3-position, were prepared following the procedure reported in our previous work [48], with modifications to the reaction time and the use of *p*-toluenesulfonic acid (PTSA, 10 mol%) as a catalyst. This involved the condensation of corresponding aromatic aldehydes (**1a–c**) with benzenesulfonohydrazides (**2a–d**) in a 1:1 molar ratio using absolute ethanol as the solvent.

Compounds **3i** and **3j** were synthesized in two steps. First, substituted benzenesulfonyl chlorides (**4a,b**) were reacted with hydrazine hydrate in ethanol to obtain sulfonylhydrazides (**2e,f**). Subsequently, 5-chloro-1*H*-indole-3-carbaldehyde (**1c**) was added to the sulfonylhydrazide products **2e,f** to yield *N*′[-1*H*-indol-3-ylmethylidene] benzenesulfonohydrazide derivatives (**3i** and **3j**), following the procedure of Navakoski de Oliveira and Jose Nunes [61], with modifications.

The indole derivative **4**, 3,3′-[(1*E*,2*E*)-hydrazine-1,2-diylidenedi(*E*)methylylidene]bis(5-chloro-1*H*-indole), was synthesized via the condensation of 5-chloroindole-3-carbaldehyde (**1c**, 0.01 mol) with hydrazine (2 mL) in absolute ethanol (15 mL) under reflux for 4 h [62]. This compound represents a new example of the class in which a 1,2-dimethylenehydrazine bridge joins two 3-substituted indole ring systems [61]. Recent in vitro studies indicate that this class of compounds exhibits strong antiproliferative activity against various cancer cell lines, including human lung cancer (A549), mouse breast cancer (4T1), human breast tumors (MDA-MB-231, MCF-7), human colon tumors (HCT), and human lung tumors (A5), showing effects comparable to cisplatin but with lower cytotoxicity toward normal cells [49,61,63].

The ^1^H NMR spectra of the indole sulfonyl hydrazide derivatives (**3a–j**) show a characteristic singlet for the sulfonyl hydrazide NH group in the 11.30–11.69 ppm range. A singlet in the 9.83–11.10 ppm range corresponds to the indole NH, except for compounds **3a** and **3b**, where the indole nitrogen is not protonated. The sulfonamide NH appears as a singlet between 9.5 and 10.5 ppm. For the 1,2-bis-indole hydrazine derivative (**4**), a singlet at 12.04 ppm is attributed to the indole NH proton.

Additionally, compounds **3a–j** and **4** display a singlet between 8.05 and 8.10 ppm, assigned to the azomethine (HC=) proton. For compound **4**, this proton is observed at 8.91 ppm. Compounds **3a**, **3c**, and **3g** feature a singlet at 3.79–3.81 ppm, corresponding to the methoxy (–OCH_3_) group at the para position of the benzene ring. Compound **3e** shows a singlet at 2.36 ppm attributed to the methyl (–CH_3_) group. Compounds **3b**, **3d**, and **3h**, bearing 2,4,6-trimethylphenyl substituents, display three singlets in the 2.23–2.69 ppm range, corresponding to the methyl groups. Similarly, compounds **3i** and **3j** exhibit two singlets between 2.25 and 2.60 ppm, attributed to methyl groups.

The ^13^C NMR spectra confirm the structures of the indole-based sulfonyl hydrazone derivatives **3a–j** and the 1,2-bis-indole hydrazine derivative **4**. Key carbon signals include C=N (azomethine) carbons in the 145–155 ppm range, aromatic and indole carbons in the 100–140 ppm range, and functional groups such as methoxy (–OCH_3_) at 55–60 ppm, methyl (–CH_3_) at 15–25 ppm, and acetyl (CH_3_–C=O) at 170–175 ppm. Compounds **3g–j** and **4** exhibit chlorinated carbons (C–Cl) in the 120–130 ppm range, whereas compounds **3c–f** show brominated carbons (C–Br) in the same region.

### 2.4. X-Ray Crystallography of N′-[(E)-(5-Bromo-1H-indol-3-yl)methylidene]-4-methylbenzene-1-sulfonohydrazide, **3e**

X-ray crystallography is crucial in pharmaceutical research for newly synthesized compounds, providing absolute configuration determination and revealing hydrogen bonding, π–π stacking, and other interactions that influence stability and biological activity.

**Figure 2:**
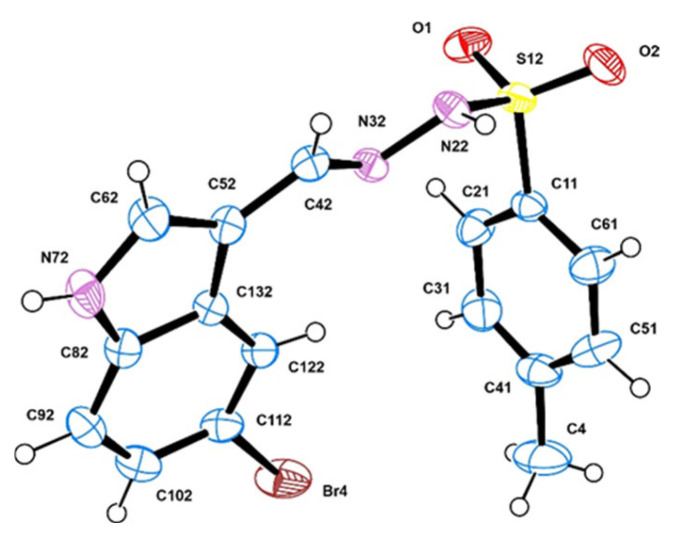
Molecular structure of compound **3e** as obtained by single-crystal X-ray structural analysis (50% ellipsoids; H atoms are shown as spheres of arbitrary radii).

Single crystals of **3e** suitable for structural analysis were successfully grown through slow evaporation from a 3:1 mixture of hexane and diethyl ether. We made several attempts to crystallize compound **3b** using different solvent systems, including ethanol, methanol, and ethyl acetate; however, it failed to produce crystals suitable for single-crystal X-ray diffraction analysis. A specimen of compound **3e** (C_16_H_15_BrN_3_O_2_S) was used for the X-ray crystallographic analysis. The most important parameters and crystal structure refinement indicators are presented in Table 5. The molecular structure of compound **3e** is shown in Figure 2.

**Figure 3:**
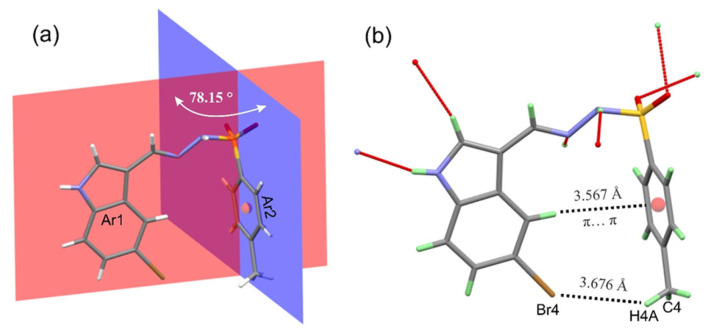
Molecular features of compound **3e**. In (**a**), the angle between the mean planes of the Ar_2_ and Ar_1_ rings is shown, providing insight into the molecular conformation. In (**b**), the focus is on the intramolecular π–π and C–H…Br interactions stabilizing the molecular conformation. Π–π interactions are depicted with black dashed lines, while intermolecular hydrogen bonds and weak C–H…O interactions are represented using red lines.

The molecular features of compound **3e** reveal that the aromatic rings (indole Ar_1_ and phenyl Ar_2_) are nearly planar, with an RMSD not exceeding 0.006 Å. The relative orientation of these two rings is characterized by a dihedral angle of 78.15° between their mean planes, indicating a non-coplanar arrangement (Figure 3a). This structural feature plays a crucial role in conformational stability and underlies the observed intramolecular interactions.

Two intramolecular interactions are illustrated in Figure 3b: a T-shaped π–π stacking interaction with a distance of 3.567 Å between the centroid and H7 and a C–H···Br halogen bond with a distance of 3.676 Å. Both interactions contribute to stabilizing the molecular conformation and may influence the overall crystal packing.

**Figure 4:**
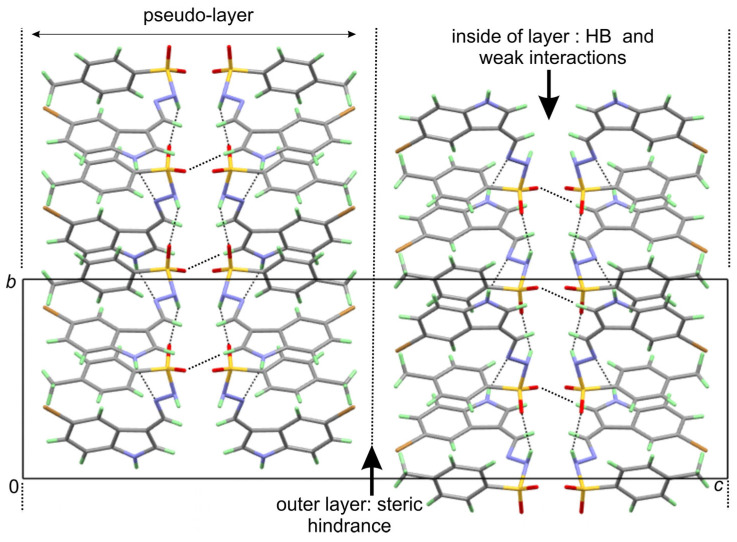
Crystal packing diagram of compound **3e**, viewed along a disclosing the formation of pseudo layer; dashed lines represent the key intermolecular interactions (NH…N, NH…O, and weak CH…O), which contribute to the stabilization of the crystal structure.

The three-dimensional crystal packing of compound **3e** (Figure 4) shows the formation of pseudo-layers, with the sulfonyl moiety positioned in the interior of these layers. The packing is governed by a network of hydrogen bonds (N–H···N and N–H···O) and weak C–H···O interactions, facilitating close packing. Meanwhile, the outer part of the layers is affected by steric hindrance from bulkier methyl groups and Br···Br repulsion. The combination of these interactions stabilizes the overall crystal growth and packing efficiency in compound **3e**.

### 2.5. Anticancer Activity of the Novel Indolyl-methylidene Substituted Phenylsulfonylhydrazones

The antiproliferative activity of the novel indolyl-methylidene-substituted phenylsulfonylhydrazones (**3a–j**) and the bis-indole hydrazine derivative (**4**) was evaluated against two breast cancer cell lines: MCF-7 and MDA-MB-231. The MCF-7 cell line, derived from human breast adenocarcinoma, expresses estrogen receptor alpha (ER-α) [64]. In contrast, the MDA-MB-231 cell line represents triple-negative breast cancer (TNBC) and lacks expression of estrogen, progesterone, and HER2 receptors [65]. Antiproliferative activity was expressed as IC_50_, the concentration (in μM) required to inhibit 50% of cell growth, and ligand efficiency (LE), calculated as LE = −log(IC_50_)/number of heavy atoms. To assess the selectivity index (SI = IC_50_ on Neuro-2a/IC_50_ on breast cancer cell line), the compounds were also tested on Neuro-2a cells, a mouse neuroblastoma cell line derived from brain tissue [66,67]. The in vitro results are summarized in Table 6.

All 11 newly designed hybrids exhibited antiproliferative activity two to seven times greater than that of cisplatin, with slightly higher potency against MCF-7 cells (avg. IC_50_ = 11.5 μM; avg. LE = 0.207) as compared to MDA-MB-231 cells (avg. IC_50_ = 20 μM; avg. LE = 0.197). Regarding selectivity, the compounds displayed higher selectivity for MCF-7 cells (avg. SI = 5) than for MDA-MB-231 cells (avg. SI = 2). For MCF-7 cells, the compounds exhibited IC_50_ values ranging from 4.0 to 29.6 μM. The most potent derivatives, compounds **3a–d** and **3f**, showed IC_50_ values below 6 μM. Ligand efficiency values ranged from 0.193 to 0.238, with compounds **3c**, **3d**, and **3f** demonstrating the highest efficiencies (LE ≥ 0.215). The selectivity index spanned from 1.176 to 21, with SI values above **3j** considered indicative of selectivity [68]. Notably, compounds **3a** and **3b** exhibited strong selectivity towards MCF-7 cells, with SI values of 13.5 and 21, respectively.

**Figure 5:**
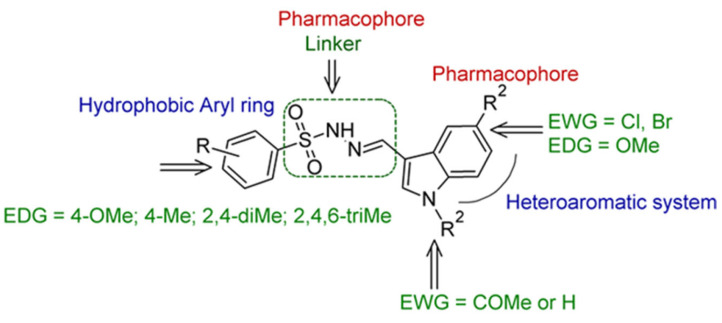
Structural features influencing activity in series **3a–j** and prior arylsulfonylhydrazone derivatives [48].

The favorable combination of steric bulk and hydrophobicity at both ends of the molecule likely contributes to its higher activity and selectivity (Figure 5).

Against MDA-MB-231 cells, IC_50_ values ranged from 4.7 to 66 μM. Compounds **3f** and **3i** were the most active, displaying IC_50_ values below 10 μM. Compound **3f** also demonstrated the highest ligand efficiency (LE = 0.242). However, none of the compounds showed significant selectivity toward MDA-MB-231 cells, as all SI values were below 10. These findings are consistent with the QSAR results, which indicated not good model predictivity for MDA-MB-231 selectivity, suggesting that TNBC’s heterogeneous nature and resistance mechanisms may require different structural features for selective targeting.

Based on our observations with sulfonylhydrazone derivatives, compounds bearing bromo or methoxy substituents exhibited neither satisfactory activity nor selectivity. Therefore, we do not expect significant improvements from introducing these groups into the bis-indole scaffold **4**. Nevertheless, the synthesis of additional analogues featuring alternative substitutions on the indole ring is planned for future studies.

### 2.6. Quantitative Structure–Activity Relationships (QSAR) of the Novel Indolyl-methylidene Substituted Phenylsulfonylhydrazones

To investigate the structure–activity relationships of the new indolyl-methylidene phenylsulfonylhydrazones, a dataset of 16 derivatives was compiled, comprising 4 compounds from the present study and 5 compounds from our previous study [48]. Compounds **1a–1e** [48] contain an indole moiety and phenyl or 4-methylphenyl substituents in the Ar position. Because these compounds provide valuable structure–activity information, they were included in the dataset for QSAR analysis performed in the present study. The molecular structures were encoded in binary format. Substituents were treated as independent variables. For each substituent, a value of 1 was assigned if it was present in the molecule and 0 if it was absent. The LEs and SIs served as dependent variables. The input matrix is given in Table S1. The data were analyzed using multiple linear regression (MLR), and the derived QSAR models were validated by cross-validation in 10 groups. The statistical measures of the derived models are presented in Table 7.

Three of the QSAR models achieved correlation coefficients R greater than 0.9 and cross-validated correlation coefficients Q above 0.65, indicating strong predictive ability. The QSAR model for SI on the MDA-MB-231 cell line yielded R of 0.666 and a negative Q, classifying it as non-predictive.

In the QSAR model for LE on MCF-7 cells, the presence of a non-substituted phenyl ring, a 4-methylphenyl group, or a 5-methoxy-indolyl-methylidene fragment positively influenced LE. Conversely, the other substituents contributed negatively to LE. For the SI model on MCF-7 cells, selectivity was affected positively by the incorporation of a non-substituted phenyl ring, 4-methylphenyl group, and 1-acetyl-indolyl-methylidene fragment in the molecules.

According to the QSAR model for LE on the MDA-MB-231 cell line, the LE was enhanced by the presence of a non-substituted phenyl ring and indolyl moieties substituted with 5-methoxy, 5-bromo, or 5-chloro groups.

These findings highlight the critical role of specific substituents in modulating both ligand efficiency and selectivity, offering valuable guidance for the future design of derivatives with improved anticancer activity.

## 3. Discussion

Guided by the structure–activity relationships (SAR) previously established for arylsulfonylhydrazones with anticancer properties [48], we designed a new series of hybrid compounds integrating both indole and phenylsulfonylhydrazone pharmacophores. The series comprised 10 indolyl-methylidene-substituted phenylsulfonylhydrazones and a bis-indole hydrazine derivative, aimed at enhancing anticancer efficacy while maintaining drug-like properties.

All compounds satisfied major drug-likeness criteria and showed promising pharmacokinetic profiles, including good oral bioavailability and absence of P-glycoprotein substrate liability—an important advantage against multidrug resistance. Nevertheless, their predicted CYP450 inhibition profiles suggest a need for caution regarding potential drug–drug interactions.

Structurally, key intramolecular interactions—such as π–π stacking and halogen bonding—contributed to the stabilization of molecular conformations, as seen in the crystal structure of compound **5**. Such interactions may impact both biological activity and physicochemical properties.

Biological evaluation showed promising anticancer activity across both tested breast cancer cell lines, with greater potency generally observed against ER-α-positive MCF-7 cells (average IC_50_ = 11.5 μM; average LE = 0.207) as compared to the triple-negative MDA-MB-231 line (average IC_50_ = 20 μM; average LE = 0.197).

The compounds also displayed higher selectivity towards MCF-7 cells (average SI = 5) relative to MDA-MB-231 cells (average SI = 2). Five compounds exhibited low micromolar potency (IC_50_ < 6 μM) against MCF-7 cells, while only two showed comparable activity against MDA-MB-231 cells. Notably, compound **3b** [*N*-[(*E*)-(1-acetyl-1*H*-indol-3-yl)methylidene]-2,4,6-trimethylbenzene-1-sulfonohydrazone] demonstrated an IC_50_ of 4 μM and an exceptional selectivity index of 21 on MCF-7 cells. In contrast, compound **3f** exhibited the highest activity on MDA-MB-231 cells (IC_50_ = 4.7 μM) but lacked selectivity. Compound **3b** emerged as the most potent and selective candidate against MCF-7 cells, combining a low micromolar IC_50_ with a high selectivity index, making it a strong lead for further development against hormone-responsive breast cancer.

When compared to our earlier series of arylsulfonylhydrazones [48], the current indole-based hybrids generally exhibited lower potency and selectivity, particularly against MCF-7 cells. Several previous derivatives, namely *N*′-[(1-acetyl-1*H*-indol-3-yl)methylidene]benzenesulfonohydrazide (1c) and *N*′-[(1-acetyl-1*H*-indol-3-yl)methylidene]-4-methylbenzenesulfonohydrazide (1d), achieved submicromolar IC_50_ values (IC_50_ = 0.9 ± 0.4 and 1.8 ± 0.6 μM, respectively) and superior selectivity indices (up to 40 and 46, respectively). While compound **3b** showed promising potency (IC_50_ = 4 μM) and a high selectivity index (SI ≈ 21), the activity against MCF-7 was slightly lower compared to our previous best analogues. This diminished activity may result from altered interactions with estrogen receptor-positive targets or reduced cellular permeability. These findings suggest that methyl substitution on the benzene ring weakens cytotoxicity compared to simpler aryl-based hydrazones and does not enhance selectivity.

The biological evaluation of compound **4** revealed moderate cytotoxic activity, particularly against the MCF-7 breast cancer cell line, with an IC_50_ of 21.4 μM. While its overall potency is lower than that of some other analogs, its structural framework remains attractive for further optimization. From a medicinal chemistry standpoint [69,70,71], bis indole derivatives holds promise as a lead structure because moderate ligand efficiency (LE) suggests that potency improvements could be achieved through rational structural modifications without significantly increasing molecular size or complexity. Additionally, the distinct selectivity toward MCF-7 cells over the triple-negative MDA-MB-231 cells highlights the potential to fine-tune its selectivity profile through targeted substitution patterns. Future directions may focus on structural optimization and introduction of substituents to enhance target interactions and cytotoxic potency, guided by computational modeling and SAR data. Although compound **4** requires further optimization, its biological profile provides a solid foundation for future drug development efforts aimed at generating more potent and selective anticancer agents within the bis-indole series.

To elucidate the individual contributions of each substituent to anticancer activity, we combined the most active compounds from the previous study with those from the current one. Their structures were binary-encoded, and a QSAR analysis was performed using MLR with 10-fold cross-validation. The resulting models clearly identified the preferred substituents on both sides of the sulfonylhydrazone linker.

For the phenyl moiety, the non-substituted (hydrogen) phenyl ring made the most significant positive contribution to LE on MCF-7 cells. Notably, the most efficient compounds, namely **1a**, **1c**, and **1e**, from the previous study [48] and compound **3f** from the current study contain a non-substituted phenyl fragment. On the indolyl-methylidene moiety, the 5-methoxy group (**1a** and **1b** from the previous study [48]) 1-acetyl substituent (as seen in compounds **1c** and **1d** from the previous study [48] and compounds **3a** and **3b** in the current study) was identified as the most favorable substituent.

In terms of selectivity towards MCF-7 cells, the non-substituted phenyl ring, the 4-methoxyphenyl group, and the 1-acetyl-indolyl-methylidene fragment were the most favorable contributors.

For activity against MDA-MB-231 cells, the combination of an unsubstituted phenyl ring (1a and 1e from the previous study [48] and **3f** in the current study) and a 5-chloro-indolyl-methylidene group showed the most positive effect. However, due to the non-predictive nature of the QSAR model for selectivity on MDA-MB-231 cells, no reliable conclusions could be drawn regarding selectivity on this cell line.

## 4. Materials and Methods

### 4.1. Materials and Reagents

The reagents necessary for the synthesis were acquired from Sigma-Aldrich (Steinheim, Germany) in analytical or chemically pure grade. The solvents used were also of analytical grade. The structure of the newly synthesized molecules was identified by ^1^H-NMR, ^13^C-NMR, and HRMS spectral data, while TLC characteristics and melting points were used to assess their purity.

The in vitro antineoplastic activity of the synthesized compounds was tested against human breast cancer cell lines, comprising the triple-negative MDA-MB-231 and the ER/PR/Her2-positive MCF-7. Neuroblastoma mouse cells Neuro-2a were used as a non-breast cancer cell line reference. All cell lines were obtained from the German Collection of Microorganisms and Cell Cultures (DSMZ, Braunschweig, Germany) and cultivated and maintained in accordance with the supplier’s protocols. The cells were cultured in RPMI 1640 growth medium (Gibco, Thermo Fisher Scientific, Waltham, MA, USA), supplemented with 10% fetal bovine serum (FBS; Gibco, Thermo Fisher Scientific, Waltham, MA, USA) and 5% L-glutamine (Gibco, Thermo Fisher Scientific, Waltham, MA, USA), and incubated at standard conditions of 37 °C in a humidified environment with 5% CO_2_.

### 4.2. In Silico Screening for Drug Likeness, ADME Properties, and Pharmacokinetic Parameters

The physicochemical properties of the designed compounds were calculated using ACD/LogD tool v. 9.08 (ACD/Labs, Toronto, ON, Canada), and using the SwissADME tool (Swiss Institute of Bioinformatics; Lausanne, Switzerland; http://www.swissadme.ch/, accessed on 15 March 2025), their ADME properties were obtained. With the use of previously constructed QSPkR models, pharmacokinetic (PK) parameters were estimated. As the fraction of ionized molecules among the majority of the designed phenylsulfonylhydrazones was less than 7%, the PK parameters were calculated using the QSPkR models for neutral molecules [56,57,58].

### 4.3. Synthesis

#### 4.3.1. General Information

NMR analyses were conducted on a Bruker Avance 400 MHz spectrometer (Bruker, Billerica, MA, USA) at 20 °C, using deuterated dimethyl sulfoxide (DMSO-*d*_6_) as the solvent and tetramethylsilane (TMS) as the internal reference. The precise assignment of ^1^H and ^13^C NMR spectra was achieved through two-dimensional (2D) homonuclear correlation (COSY), DEPT-135, and 2D inverse-detected heteronuclear (C–H) correlation techniques, including heteronuclear single-quantum correlation (HMQC) and heteronuclear multiple bond correlation (HMBC) spectroscopy. Mass spectrometry was performed using a Q Exactive Plus mass spectrometer (Thermo Fisher Scientific, Waltham, MA, USA) equipped with a Heated Electrospray Ionization (HESI-II) probe (Thermo Fisher Scientific, Bremen, Germany). Melting points were determined using a Büchi 535 apparatus (Büchi Labortechnik AG, Flawil, Switzerland) and an M5000 melting point meter (Krüss Optronic GmbH, Hamburg, Germany). The nomenclature of the synthesized compounds adheres to IUPAC conventions.

#### 4.3.2. General Procedure for the Synthesis of the Compounds **3a–h**

A 10 mL ethanolic solution of the respective carbonyl compound (0.002 mol), 1-acetyl-1*H*-indole-3-carbaldehyde (**1a**), 5-bromo-1*H*-indole-3-carbaldehyde (**1b**), or 5-chloro-1*H*-indole-3-carbaldehyde (**1c**) was added to a hot solution (60 °C) of 0.002 mol of either 4-methoxybenzenesulfonohydrazide (**2a**), 2,4,6-trimethylbenzenesulfonohydrazide (**2b**), 4-methylbenzenesulfonohydrazide (**2c**), or benzenesulfonohydrazide (**2d**) in 10 mL of absolute ethanol. The reaction mixture was stirred for 1–6 h in the presence of *p*-toluenesulfonic acid (PTSA, 10 mol%) as a catalyst. The progress of the reaction was monitored by thin-layer chromatography (TLC) on precoated silica gel 60 F254 aluminum plates (Merck KGaA, Darmstadt, Germany) using hexane/ethyl acetate (8:2, *v*/*v*) as the eluent, and spots were visualized under UV light at 254 nm. After cooling, the resulting crystalline precipitates were filtered, washed with an ethanol–ether mixture, recrystallized from ethanol, and dried. The newly synthesized compounds appear as light-yellow solids stable under normal conditions and soluble in methanol, acetonitrile, and DMSO while also exhibiting low solubility in water and ethanol. For the numbering of the compounds and spectra, see the Suppmlentary Materials (Figures S1–S74).

##### N′-[(E)-(1-Acetyl-1H-indol-3-yl)methylidene]-4-methoxybenzene-1-sulfonohydrazide, **3a**

Light-yellow solid. Yield: 92%; m.p. 188–190 °C.

^1^H-NMR (DMSO-*d*_6_) δ 2.63 (s, 3H, CH_3_), 3.79 (s, 3H, CH_3_), 7.12 (d, *J* = 9.0 Hz, 2H, H-3′ and H-5′), 7.34–7.41 (m, 2H, H-5 and H-6), 7.85 (d, *J* = 9.0 Hz, 2H, H-2′ and H-6′), 8.05–8.07 (m, 1H, H-7), 8.08 (s, 1H, =CH), 8.25 (s, 1H, H-2), 8.31-8.33 (n, 1H, H-4), 11.30 (bs, 1H, NH indole). ^13^C-NMR (101 MHz, DMSO-*d*_6_) δ 23.79 (CH_3_), 55.65 (CH_3_), 114.35 (C-3′ and C-5′), 115.87 (C-4), 116.28 (C-3), 122.07 (C-7), 124.22 (C-6), 125.68 (C-5), 126.42 (C-4a), 129.44 (C-2′ and C-6′), 130.53 (C-1′), 130.92 (C-2), 135.70 (C-7a), 142.27 (=CH), 162.61 (C-4′), 169.57 (C=O). HRMS *m/z* [M + H]+ exp/[M + H]+ calcd: 372.1006/372.101252.

##### N′-[(E)-(1-Acetyl-1H-indol-3-yl)methylidene]-2,4,6-trimethylbenzene-1-sulfonohydrazide, **3b**

Light-yellow solid. Yield: 81%; m.p. 214–216 °C.

^1^H-NMR (400 MHz, DMSO-*d*_6_) δ 2.23 (s, 3H, CH_3_), 2.63 (s, 3H, CH_3_), 2.67 (s, 6H, CH_3_), 7.07 (bs, 2H, H-3′ and H-5′), 7.25 (t, *J* = 7.4 Hz, 1H, H-6), 7.35 (t, *J* = 7.8 Hz, 1H, H-5), 7.88 (d, *J* = 7.8 Hz, 1H, H-7), 8.10 (s, 1H, =CH), 8.23 (s, 1H, H-2), 8.31 (d, *J* = 8.2 Hz, 1H, H-4), 11.54 (bs, 1H, NH indole). ^13^C-NMR (101 MHz, DMSO-*d*_6_) δ 20.41 (CH_3_), 22.65 (CH_3_), 23.80 (CH_3_), 115.91 (C-4), 116.34 (C-3), 121.83 (C-7), 123.84 (C-6), 125.66 (C-5), 126.36 (C-4a), 130.70 (C-2), 131.63 (C-3′ and C-5′), 133.13 (C-4′), 135.69 (C-7a), 139.22 (C-2′ and C-6′), 140.72 (=CH), 142.30 (C-1′), 169.58 (C=O). HRMS *m/z* [M + H]+exp/[M + H]+ calcd: 384.1368/384.137638.

##### N′-[(E)-(5-Bromo-1H-indol-3-yl)methylidene]-4-methoxybenzene-1-sulfonohydrazide, **3c**

Light-yellow solid. Yield: 91%; m.p. 190–192 °C.

^1^H-NMR (400 MHz, DMSO-*d*_6_) δ 3.81 (s, 3H, CH_3_), 7.14 (d, *J* = 9.0 Hz, 2H, H-3′ and H-5′), 7.28 (dd, *J* = 2.0, 8.6 Hz, 1H, H-6), 7.37 (d, *J* = 8.6 Hz, 1H, H-7), 7.77 (bs, 1H, H-2), 7.84 (d, *J* = 9.0 Hz, 2H, H-2′ and H-6′), 8.05 (s, 1H, =CH), 8.06 (bs, 1H, H-4), 10.80 (bs, 1H, NH), 11.68 (bs, 1H, NH indole). ^13^C-NMR (101 MHz, DMSO-*d*_6_) δ 55.64 (CH_3_), 110.67 (C-3), 113.03 (C-5), 113.84 (C-7), 114.23 (C-3′ and C-5′), 123.84 (C-4), 125.00 (C-6), 125.68 (C-4a), 129.53 (C-2′ and C-6′), 130.62 (C-1′), 131.49 (C-3), 135.56 (C-7a), 144.45 (=CH), 162.48 (C-4′). HRMS *m/z* [M + H]+exp/[M + H]+ calcd: 408.0008 (99.49), 409.9983 (100.00)/408.001193.

##### N′-[(E)-(5-Bromo-1H-indol-3-yl)methylidene]-2,4,6-trimethylbenzene-1-sulfonohydrazide, **3d**

Light-yellow solid. Yield: 91%; m.p. 211–213 °C.

^1^H-NMR (400 MHz, DMSO-*d*_6_) δ 2.24 (s, 3H, CH_3_), 2.69 (s, 6H, CH_3_), 7.07 (bs, 2H, H-3′ and H-5′), 7.25 (dd, *J* = 2.0, 8.6 Hz, 1H, H-6), 7.35 (d, *J* = 8.6 Hz, 1H, H-7), 7.75 (bs, 1H, H-2), 7.93 (d, *J* = 1.8 Hz, 1H, H-4), 8.07 (s, 1H, =CH), 11.00 (bs, 1H, NH), 11.67 (bs, 1H, NH indole). ^13^C-NMR (101 MHz, DMSO-*d*_6_) δ 20.44 (CH_3_), 22.81 (CH_3_), 110.73 (C-3), 112.95 (C-5), 113.79 (C-7), 123.58 (C-4), 124.99 (C-6), 125.50 (C-4a), 131.37 (C-2), 131.66 (C-3′ and C-5′), 133.18 (C-4′), 135.56 (C-7a), 139.20 (C-2′ and C-6′), 141.99 (C-1′), 142.62 (=CH). HRMS *m/z* [M + H]+ exp/[M + H]+ calcd: 420.0370 (98.84), 422.0344 (100.00)/420.037578.

##### N′-[(E)-(5-Bromo-1H-indol-3-yl)methylidene]-4-methylbenzene-1-sulfonohydrazide, **3e**

Light-yellow solid. Yield: 87%; m.p. 223–225 °C.

^1^H-NMR (400 MHz, DMSO-*d*_6_) δ 2.36 (s, 3H, CH_3_), 7.29 (dd, *J* = 2.0, 8.6 Hz, 1H, H-6), 7.38 (d, *J* = 8.6 Hz, 1H, H-7), 7.43 (d, *J* = 8.0 Hz, 2H, H-3′ and H-5′), 7.77 (bs, 1H, H-2), 7.80 (d, *J* = 8.3 Hz, 2H, H-2′ and H-6′), 8.06 (s, 1H, =CH), 8.07 (d, *J* = 1.9 Hz, 1H, H-4), 10.92 (bs, 1H, NH), 11.69 (bs, 1H, NH indole). ^13^C-NMR (101 MHz, DMSO-*d*_6_) δ 21.00 (CH_3_), 110.63 (C-3), 113.04 (C-5), 113.85 (C-7), 123.84 (C-4), 125.01 (C-6), 125.67 (C-4a), 127.39 (C-2′ and C-6′), 129.51 (C-3′ and C-5′), 131.56 (C-2), 135.56 (C-7a), 136.12 (C-4′), 143.27 (C-1′), 144.53 (=CH). HRMS *m/z* [M + H]+Exp/[M + H]+calcd: 392.0057 (98.20), 394.0034 (100.00)/392.006278.

##### N′-[(E)-(5-Bromo-1H-indol-3-yl)methylidene]benzene-1-sulfonohydrazide, **3f**

Light-yellow solid. Yield: 90%; m.p. 221–223 °C.

^1^H-NMR (400 MHz, DMSO-*d*_6_) δ 7.28 (dd, *J* = 2.0, 8.6 Hz, 1H, H-6), 7.37 (dd, *J* = 0.4, 8.6 Hz, 1H, H-7), 7.62–7.67 (m, 3H, H-3′, H-4′ and H-5′), 7.78 (d, *J* =2.7 Hz, 1H, H-2), 7.91–7.93 (m, 2H, H-2′ and H-6′), 8.07 (d, *J* = 2.0 Hz, 1H, H-4), 8.08 (s, 1H, =CH), 11.03 (bs, 1H, NH), 11.69 (bs, 1H, NH indole). ^13^C-NMR (101 MHz, DMSO-*d*_6_) δ 110.55 (C-3), 113.07 (C-5), 113.87 (C-7), 123.81 (C-4), 125.03 (C-6), 125.65 (C-4a), 127.35 (C-2′ and C-6′), 129.09 (C-3′ and C-5′), 131.69 (C-2), 132.97 (C-4′), 135.57 (C-7a), 138.93 (C-1′), 144.84 (=CH). HRMS *m/z* [M+H]+ exp/[M + H]+ calcd: 377.9901 (97.81), 379.9876 (100.00)/377.990628.

##### N′-[(E)-(5-Chloro-1H-indol-3-yl)methylidene]-4-methoxybenzene-1-sulfonohydrazide, **3g**

Light-yellow solid. Yield: 87%; m.p. 214–215 °C.

^1^H-NMR (400 MHz, DMSO-*d*_6_) δ 3.80 (s, 3H, CH_3_), 7.13 (d, *J* = 9.0 Hz, 2H, H-3′ and H-5′), 7.17 (dd, *J* = 2.2, 8.6 Hz, 1H, H-6), 7.41 (d, *J* = 8.6 Hz, 1H, H-7), 7.79 (d, *J* = 2.8 Hz, 1H, H-2), 7.83 (d, *J* = 8.9 Hz, 2H, H-2′ and H-6′), 7.89 (d, *J* =2.1 Hz, 1H, H-4), 8.06 (s, 1H, = CH), 10.85 (bs, 1H, NH), 11.67 (bs, 1H, NH indole). ^13^C-NMR (101 MHz, DMSO-*d*_6_) δ 55.63 (CH_3_), 110.75 (C-3), 113.40 (C-7), 114.24 (C-3′ and C-5′), 120.72 (C-4), 122.50 (C-6), 125.03 (C-5), 129.51 (C-2′ and C-6′), 130.59 (C-1′), 131.72 (C-2), 135.32 (C-7a), 144.54 (=CH), 162.51 (C-4′). HRMS *m/z* [M + H]+ exp/[M + H]+ calcd: 364.0509 (100.00), 366.0480 (37.42)/364.051715.

##### N′-[(E)-(5-Chloro-1H-indol-3-yl)methylidene]-2,4,6-trimethylbenzene-1-sulfonohydrazide, **3h**

Light-yellow solid. Yield: 95%; m.p. 210–211 °C.

^1^H-NMR (400 MHz, DMSO-*d*_6_) δ 2.23 (s, 3H, CH_3_), 2.69 (s, 6H, CH_3_), 7.06 (bs, 2H, H-3′ and H-5′), 7.14 (dd, *J* = 2.2, 8.6 Hz, 1H, H-6), 7.39 (d, *J* = 8.6 Hz, 1H, H-7), 7.77 (d, *J* = 2.8 Hz, 1H, H-2), 7.78 (d, *J* = 2.1 Hz, 1H, H-4), 8.07 (s, 1H, =CH), 11.07 (bs, 1H, NH), 11.64 (bs, 1H, NH indole). ^13^C-NMR (101 MHz, DMSO-*d*_6_) δ 20.44 (CH_3_), 22.79 (CH_3_), 110.83 (C-3), 113.33 (C-7), 120.57 (C-4), 122.46 (C-6), 124.88 (C-5), 124.98 (C-4a), 131.54 (C-2), 131.66 (C-3′ and C-5′), 133.17 (C-1′), 135.30 (C-7a), 139.22 (C-2′ and C-6′), 142.05 (C-4′), 142.67 (=CH). HRMS *m/z* [M + H]+ exp/[M + H]+ calcd: 376.0873 (100.00)/376.088101.

#### 4.3.3. General Procedure for the Synthesis of the Compounds **3i,3g**

A solution of 0.01 mol of either 2,4-dimethylbenzenesulfonyl chloride (**4a**) or 3,4-dimethylbenzenesulfonyl chloride (**4b**) in 5 mL of absolute ethanol was stirred, while hydrazine hydrate (0.01 mol, 80%) was added dropwise, maintaining the temperature at 0 °C. The mixture was then refluxed for six hours, allowed to cool to room temperature, and poured into crushed ice with continuous stirring. The resulting products **2e** and **2f** were filtered, dried, and recrystallized from ethanol, yielding a pale-yellow solid powder. Then, in a 50 mL round-bottom flask, 2,4-dimethylbenzenesulfonylhydrazide **2e** (1.0 mmol) or 3,4-dimethylbenzenesulfonylhydrazide **2f** (1.0 mmol) and 5-chloro-1*H*-indole-3-carbaldehyde **1c** (1.0 mmol) were dissolved in 10 mL of ethanol. To catalyze the reaction, p-toluenesulfonic acid (PTSA, 10 mol%) was added. The reaction mixture was stirred at room temperature for 2–4 h. Reaction progress was monitored by thin-layer chromatography (TLC) using ethyl acetate/hexane (3:1) as the mobile phase. The resulting product was filtered, dried, and recrystallized from ethanol, yielding a light-yellow solid.

##### N′-[(E)-(5-Chloro-1H-indol-3-yl)methylidene]-2,4-dimethylbenzene-1-sulfonohydrazide, **3i**

Light-yellow solid. Yield: 89%; m.p. 166–167 °C.

^1^H-NMR (400 MHz, DMSO-*d*_6_): δ 2.30 (s, 3H, CH_3_), 2.60 (s, 3H, CH_3_), 7.14 (dd, *J* = 2.2, 8.6 Hz, 1H, H-6), 7.20 (d, *J* = 0.5 Hz, 1H, H-3′), 7.24 (dd, *J* = 1.7, 8.2 Hz, 1H, H-5′), 7.39 (dd, *J* = 0.4, 8.6 Hz, 1H, H-7), 7.73 (d, *J* = 2.2 Hz, 1H, H-4), 7.77 (d, *J* =2.8 Hz, 1H, H-2), 7.88 (d, *J* = 8.1 Hz, 1H, H-6′), 8.08 (s, 1H, =CH), 11.10 (s, 1H, NH), 11.64 (d, *J* = 1.8 Hz, 1H, NH indole). ^13^C-NMR (101 MHz, DMSO-*d*_6_) δ 20.07 (CH_3_), 20.76 (CH_3_), 110.79 (C-3), 113.32 (C-7), 120.73 (C-4), 122.46 (C-6), 124.93 (C-5), 124.95 (C-4a), 126.76 (C-5′), 130.07 (C-6′), 131.50 (C-2), 132.93 (C-3′), 134.41 (C-1′), 135.30 (C-7a), 136.93 (C-2′), 143.14 (C-4′), 143.19 (=CH). HRMS *m/z* [M + H] exp/[M + H]+ calcd: 362.0712 (100.00)/362.072451.

##### N′-[(E)-(5-Chloro-1H-indol-3-yl)methylidene]-3,4-dimethylbenzene-1-sulfonohydrazide, **3j**

Light-yellow solid. Yield: 79%; m.p. 210–212 °C.

^1^H-NMR (400 MHz, DMSO-*d*_6_) δ 2.25 (s, 3H, CH_3_), 2.29 (s, 3H, CH_3_), 7.18 (dd, *J* = 2.2, 8.6 Hz, 1H, H-6), 7.36 (d, *J* = 8.0 Hz, 1H, H-5′), 7.42 (dd, *J* = 0.3, 8.6 Hz, 1H, H-7), 7.62 (dd, *J* = 1.9, 7.9 Hz, 1H, H-6′), 7.71 (d, *J* = 1.4 Hz, 1H, H-2′), 7.79 (d, *J* = 2.8 Hz, 1H, H-2), 7.94 (d, *J* = 2.2 Hz, 1H, H-4), 8.05 (s, 1H, =CH), 10.88 (s, 1H, NH), 11.67 (d, *J* =1.3 Hz, 1H, NH indole). ^13^C-NMR (101 MHz, DMSO-*d*_6_) δ 19.42 (CH_3_), 110.75 (C-3), 113.42 (C-7), 120.74 (C-4), 122.52 (C-6), 124.78 (C-6′), 125.00 (C-5), 125.01 (C-4a), 128.17 (C-2′), 129.91 (C-5′), 131.80 (C-2), 135.35 (C-1′), 136.21 (C-7a), 137.29 (C-3′), 142.09 (C-4′), 144.45 (=CH). HRMS *m/z* [M+H]+ exp/[M + H]+ calcd: 362.0712 (100.00)/362.072451.

#### 4.3.4. General Procedure for the Synthesis of the 5-Chloro-3-[(E)-[(2E)-[(5-chloro-1H-indol-3-yl)methylidene]hydrazinylidene]methyl]-1H-indole, Alternative Name is 3,3′-[(1E,2E)-Hydrazinediylidenedi-(E)-methanylylidene]bis(5-chloro-1H-indole) **4**

The appropriate amount of 5-chloroindole-3-carbaldehyde, **1c** (0.4 g, 0.01 mol) (2 equivalents), was dissolved in ethanol under continuous stirring. The hydrazine hydrate (2 mL) (1 equivalent) was slowly added dropwise while constantly stirring on reflux at 60 °C for 8 h. The progress of the reaction was monitored using thin-layer chromatography (TLC). After cooling the reaction mixture, the resulting solid was collected by filtration. The crude product was washed thoroughly with cool ethanol and purified using recrystallization to obtain the final bis(indolyl methylidene)diazine product (**4**) as white crystals (78% yield); m.p. 266–268 °C. ^1^H-NMR (400 MHz, DMSO-*d*_6_) δ 7.23 (dd, *J* = 2.1, 8.6 Hz, 2H, H-6), 7.51 (d, *J* = 8.6 Hz, 2H, H-7), 8.00 (d, *J* = 2.7 Hz, 2H, H-2), 8.33 (d, *J* = 2.0 Hz, 2H, H-4), 8.91 (s, 2H, =CH), 12.04 (bs, 2H, NH indole). ^13^C-NMR (101 MHz, DMSO-*d*_6_) δ 111.61 (2C-3), 113.62 (2C-7), 121.15 (2C-4), 122.57 (2C-6), 125.18 (2C-5), 125.74 (2C-4a), 133.49 (2C-2), 135.69 (2C-7a), 155.24 (2C, =CH). HRMS *m/z* [M + H]+ exp/[M + H]+ calcd: 355.0504/355.05118.

#### 4.3.5. Single-Crystal X-Ray Analysis of *N*′-[(*E*)-(5-Bromo-1*H*-indol-3-yl)methylidene]-4-methylbenzene-1-sulfonohydrazide, **3e**

Single-crystal X-ray diffraction data were collected on a Bruker D8 Venture diffractometer equipped with a PhotonII CMOS detector (Bruker, Billerica, MA, USA), using mirror-monochromatized Mo Kα radiation (λ = 0.71073 Å) from a micro-focus source. The total exposure time was 1.40 h. The frames were integrated with the Bruker SAINT ver. 8.40B software package. The integration of the data using an orthorhombic unit cell yielded a total of 63,430 reflections to a maximum θ angle of 29.30° (0.73 Å resolution). The final cell constants of a = 8.9209(13) Å, b = 10.1438(16) Å, c = 35.817(5) Å, α = 90°, β = 90°, γ = 90°, and volume = 3241.2(14) Å3 are based upon the refinement of the XYZ-centroids of 9255 reflections above 20 σ(I) with 4.549° < 2θ < 58.20°. Data were corrected for absorption effects using the multi-scan method (SADABS) (Bruker, A. Saint and SADABS) [72]. The ratio of minimum to maximum apparent transmission was 0.504. The structure was solved, and the space group Pbca (number 61) determined by the ShelXT 2018/2 [73] structure solution program using intrinsic methods and refined by full matrix least squares minimization on F2 using version 2019/1 of XL [74]. All non-hydrogen atoms were refined anisotropically. Hydrogen atom positions were positioned from the difference Fourier map and refined isotopically. The key data collection of parameters and crystal structure refinement indicators is summarized in Table 5, while Figure 2 illustrates the asymmetric unit of compound **3e**.

The structural analysis crystallographic data (excluding structure factors) were deposited with the Cambridge Crystallographic Data Centre, CCDC No. 2425493. A copy of this information may be obtained free of charge from The Director, CCDC, 12 Union Road, Cambridge CB2 1EZ, UK. 6 January 2024 Fax: +44-1223-336-033, e-mail: deposit@ccdc.cam.ac.uk, or www.ccdc.cam.ac.uk, accessed on 20 February 2025.

### 4.4. In Vitro Anticancer Activity

The cytostatic activity of the tested compounds was evaluated using the Mosmann MTT assay—a well-established colorimetric method for assessing cell viability [75]. This assay measures mitochondrial enzyme activity by reducing the yellow dye MTT (3-(4,5-dimethylthiazol-2-yl)-2,5-diphenyltetrazolium bromide) into violet formazan crystals. The exponentially growing cells were harvested and seeded into 96-well plates at a density of 1.5 × 10^5^ cells per well in 100 μL of medium and incubated for 24 h. The cell cultures were then treated with various concentrations (200–6.25 μM) of the tested compounds and incubated for 72 h. Cell viability was quantified as a percentage (%) relative to the untreated control, which was set at 100% viability.

The experimental data were analyzed using nonlinear regression in GraphPad Prism® software, version 9.0. Semi-logarithmic dose–response curves were generated, and the half-maximal inhibitory concentrations (IC_50_) of the tested compounds were calculated for each tumor cell line.

### 4.5. Quantitative Structure-Activity Relationship (QSAR) Protocol

The structures of the newly synthesized compounds were binary-encoded. Substituents were treated as independent variables, while ligand efficiencies (LE = pIC_50_/number of heavy atoms) and similarity indices (SI = IC_50_ on the reference cell line/IC_50_ on the test cell line) were considered dependent variables. If a particular substituent was present in the molecule, its corresponding variable was assigned a value of 1; otherwise, it was assigned a value of 0. The data were analyzed using multiple linear regression (MLR), implemented in the software tool Weka (htpps://weka.io, accessed on 20 April 2025), and the resulting QSAR models were validated by internal cross-validation in 10 groups. Only models with a correlation coefficient (R) greater than 0.7 and a cross-validated correlation coefficient (Q) above 0.6 were considered to possess predictive ability.

## 5. Conclusions

This study demonstrates the therapeutic potential of indolyl-methylidene phenylsulfonylhydrazones (compounds **3a–j**) as promising candidates for breast cancer treatment. Through computational design, synthesis, and biological evaluation, we identified key structural features driving selective cytotoxicity toward ER-α-positive breast cancer cells. X-ray crystallography of compound **3e** and QSAR analyses highlighted the critical roles of indolyl substitutions and phenyl ring modifications in modulating activity. Among the tested compounds, compound **3b** (IC_50_ = 4.0 ± 0.9 μM; SI = 20.975) emerged as the most potent and selective against ER/PR/Her2-positive MCF-7 cells, while compound **3f** (IC_50_ = 4.7 ± 1.4 μM) was the most potent against MDA-MB-231 cells but lacked selectivity. Notably, compound **4** (IC_50_ = 21.4 ± 4.9 μM) showed moderate activity yet favorable ligand efficiency, making it a valuable scaffold for further optimization. Future efforts will focus on structural modifications, mechanistic studies, and structure-guided design to enhance potency, selectivity, and drug-like properties. To further substantiate the in vitro findings, in vivo studies, including preliminary toxicity assessments of compound **3b**, are currently underway. Although specific molecular targets have not been definitively identified, we intend to perform in vitro enzymatic assays and target validation studies in future work to explore possible mechanisms of action. These efforts will focus particularly on tubulin polymerization and topoisomerase II inhibition, guided by structural and functional insights. Overall, this study provides a solid foundation for advancing indole-based phenylsulfonylhydrazones as anticancer agents, particularly for hormone-dependent breast cancer.

## Figures and Tables

**Table 1:** Newly designed indolyl-methylidene substituted phenylsulfonylhydrazones (**3a–j**) and 3,3′-[(1*E*,2*E*)-hydrazine-1,2-diylidenedi(*E*)methylylidene]bis(5-chloro-1*H*-indole) (**4**).

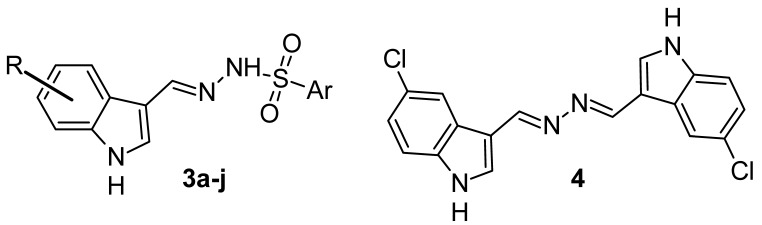		
ID	R	Ar
**3a**	1-acetyl	4-methoxyphenyl
**3b**	1-acetyl	2,4,6-trimethylphenyl
**3c**	5-bromo	4-methoxyphenyl
**3d**	5-bromo	2,4,6-trimethylphenyl
**3e**	5-bromo	4-methylphenyl
**3f**	5-bromo	phenyl
**3g**	5-chloro	4-methoxyphenyl
**3h**	5-chloro	2,4,6-trimethyphenyl
**3i**	5-chloro	2,4-dimethylphenyl
**3j**	5-chloro	3,4-dimethylphenyl
**4**	5-chloro	1-(5-chloro-1*H*-indol-3-yl)methanimine

**Table 2:** Physicochemical properties of the newly designed substituted indole derivatives (**3a–j**) with phenylsulfonyl hydrazone fragment (–C=N–NH–SO_2_–Ar) and 1,2-bis-indole hydrazine derivative (**4**).

ID	*Mw*	*pK_a_*(*A*)	*pK_a_*(*B*)	*f_A_*	*f_B_*	*logP*	*logD*_7.4_	*PSA*	*FRB*	*HBD*	*HBA*
**3a**	371.4	8.92		0.03		3.37	3.36	98.1	4	1	7
**3b**	383.5	8.87		0.03		4.23	4.22	88.9	3	1	6
**3c**	408.3	9.29		0.01		4.19	4.19	91.9	4	2	6
**3d**	420.3	9.24		0.01		5.05	5.05	82.7	3	2	5
**3e**	392.3	9.20		0.02		4.13	4.13	82.7	3	2	5
**3f**	378.2	8.96		0.03		3.67	3.67	82.7	3	2	5
**3g**	363.8	9.32		0.01		4.12	4.12	91.9	4	2	6
**3h**	375.9	9.26		0.01		4.98	4.98	82.7	3	2	5
**3i**	361.9	9.24		0.01		4.52	4.52	82.7	3	2	5
**3j**	361.9	9.24		0.01		4.52	4.52	82.7	3	2	5
**4**	355.2		6.28		0.07	5.43	5.40	56.3	3	3	4

*Mw*—molecular weight; *pK_a_*(*A*); *pK_a_*(*B*); *f_A_* and *f_B_*—fractions of the ionized molecules; *logP*; *logD*_7.4_—distribution coefficient at pH 7.4; *PSA*—polar surface area; *FRB*—free rotatable bonds; *HBD*—hydrogen bond donors; *HBA*—hydrogen bond acceptors.

**Table 3:** ADME properties of the newly designed substituted indole derivatives (**3a−j**) with phenylsulfonylhydrazone fragment (–C=N–NH–SO_2_–Ar) and 1,2-bis-indole hydrazine derivative (**4**): *water solubility*; *GI absorption*—gastrointestinal absorption; *oral BA*—oral bioavailability; *BA score*—bioavailability score; *BBB permeability*—blood–brain barrier permeability; *CYP inhibition*—inhibition of CYP enzymes; *P-gp substrate*—substrate of P-gp; *drug likeness*; *synthetic access*—synthetic accessibility.

ID	*Water* *Solubility*	*GI* *Absorption*	*Oral BA*	*BA Score*	*BBB Permeability*	*CYP* *Inhibition*	*P-gp* *Substrate*	*Drug* *Likeness*	*Synthetic Access*
**3a**	moderate	high	INSATU	0.55	no	2/5	no	yes	2.78
**3b**	moderate	high	INSATU	0.55	no	3/5	no	yes	3.07
**3c**	moderate	high	INSATU	0.55	no	4/5	no	yes	2.67
**3d**	poor	high	INSATU	0.55	no	4/5	no	yes	2.96
**3e**	moderate	high	INSATU	0.55	no	4/5	no	yes	2.67
**3f**	moderate	high	INSATU	0.55	no	4/5	no	yes	2.57
**3g**	moderate	high	INSATU	0.55	no	4/5	no	yes	2.65
**3h**	poor	high	INSATU	0.55	no	4/5	no	yes	2.95
**3i**	moderate	high	INSATU	0.55	no	4/5	no	yes	2.84
**3j**	moderate	high	INSATU	0.55	no	4/5	no	yes	2.84
**4**	poor	high	INSATU	0.55	yes	4/5	no	yes	2.40

**Table 4:** PK parameters of the newly designed substituted indole derivatives (**3a–j**) with phenylsulfonylhydrazone fragment (–C=N–NH–SO_2_–Ar) and 3,3′-[(1*E*,2*E*)-hydrazine-1,2- diylidenedi(*E*)methylylidene]bis(5-chloro-1*H*-indole) (**4**): *fu—*fraction of the compound unbound to plasma proteins; *CL*—total clearance in L/h/kg; *VDss*—steady-state volume of distribution in L/kg; *t*_1/2_—half-life in h.

ID	*fu*	*CL* L/h/kg	*VDss* L/kg	*t*_1/2_ h
**3a**	0.016	0.407	0.733	1.25
**3b**	0.010	0.445	1.330	2.07
**3c**	0.018	0.483	1.238	1.78
**3d**	0.006	0.277	1.776	4.44
**3e**	0.014	0.504	1.253	1.72
**3f**	0.022	0.166	1.059	4.43
**3g**	0.012	0.328	1.042	2.20
**3h**	0.005	0.237	1.623	4.74
**3i**	0.007	0.293	1.356	3.21
**3j**	0.007	0.290	1.362	3.26
**4**	0.002	0.373	2.730	5.07

**Table 5:** The most important parameters and crystal structure refinement indicators for compound **3e**.

Compound	3e
Empirical formula	C_16_H_14_BrN_3_O_2_S
Formula weight (MW)	392.27
Temperature/K	286.00
Crystal system	Orthorhombic
Space group	*Pbca*
*a*/Å	8.9209(13)
*b*/Å	10.1438(16)
*c*/Å	35.817(5)
*α*/°	90
*β*/°	90
*γ*/°	90
Volume/Å^3^	3241.2(8)
*Z*	8
ρ_calc_g/cm^3^	1.608
μ/mm^−1^	2.678
F_(000)_	1584.0
Crystal size/mm^3^	0.2 × 0.15 × 0.14
Radiation	MoKα (λ = 0.71073)
*2Θ* range for data collection/°	4.548 to 51.362
Index ranges	−10 ≤ *h* ≤ 9, −11 ≤ *k* ≤ 12, −43 ≤ *l* ≤ 43
Reflections collected/reflections	33,099/307
Data/restraints/parameters	3075/0/264
Goodness-of-fit on F^2^	1.151
	*R_int_* = 0.0407, R_sigma_ = 0.0224
Final *R* indexes [*I* ≥ 2*σ* (*I*)]	*R*_1_ = 0.0403, *wR*_2_ = 0.0876
Final *R* indexes [all data]	*R*_1_ = 0.0477, *wR*_2_ = 0.0906
Largest diff. peak/hole/e Å^−3^	0.33/−0.50
CCDC number	2425493

**Table 6:** Cytotoxicity of the newly designed substituted indole derivatives (**3a–j**) with phenylsulfonyl hydrazone fragment (–C=N–NH–SO_2_–Ar) and 1,2-bis-indole hydrazine derivative (**4**) on *MCF*-7 (ER-positive breast adenocarcinoma), *MDA-MB*-231 (triple-negative breast cancer), and N2a (mouse neuroblastoma) cell lines. *LE*—ligand efficiency; *SI*—selectivity index.

ID	*MCF-*7*^a^*			*MDA-MB-*231*^b^*			*Neuro-*2*a^c^*	
	*IC*_50_ μM	*LE*	*SI*	*IC*_50_ μM	*LE*	*SI*	*IC*_50_ μM	*LE*
**3a**	4.3 ± 1.3	0.206	13.488	20.1 ± 3.4	0.181	2.886	58 ± 8.2	0.163
**3b**	4.0 ± 0.9	0.200	20.975	16.3 ± 4.1	0.177	5.147	83.9 ± 9.4	0.151
**3c**	5.2 ± 1.0	0.220	3.577	17.7 ± 2.3	0.198	1.051	18.6 ± 3.9	0.197
**3d**	4.2 ± 0.7	0.215	5.000	19.9 ± 1.6	0.188	1.055	21.0 ± 2.1	0.187
**3e**	29.6 ± 5.1	0.197	1.176	18.7 ± 2.7	0.206	1.861	34.8 ± 5.7	0.194
**3f**	5.9 ± 1.4	0.238	1.847	4.7 ± 1.4	0.242	2.319	10.9 ± 1.8	0.226
**3g**	12.7 ± 2.8	0.204	2.063	14.7 ± 1.8	0.201	1.782	26.2 ± 3.5	0.191
**3h**	15.2 ± 2.1	0.193	1.579	18.1 ± 2.5	0.190	1.326	24.0 ± 1.9	0.185
**3i**	12.4 ± 3.2	0.204	1.282	9.5 ± 1.5	0.209	1.674	15.9 ± 1.6	0.200
**3j**	11.5 ± 2.3	0.206	3.087	13.3 ± 2.2	0.203	2.669	35.5 ± 5.3	0.185
**4**	21.4 ± 4.9	0.195	2.509	66.0 ± 7.4	0.174	0.814	53.7 ± 3.1	0.178
Cisplatin	50.3 ± 6.5			63.4 ± 7.2			-	

*^a^* hormone-responsive breast adenocarcinoma; *^b^* triple-negative breast cancer; *^c^* mouse neuroblastoma.

**Table 7:** QSAR models of the newly designed substituted indole derivatives (**3a–j**) with phenylsulfonylhydrazone fragment (–C=N–NH–SO_2_–Ar) and 3,3′-[(1*E*,2*E*)-hydrazine-1,2- diylidenedi(*E*)methylylidene]bis(5-chloro-1*H*-indole) (**4**). *R*—correlation coefficient; *RMSE*—root mean squared error; *Q*—correlation coefficient cross-validated in 10 groups.

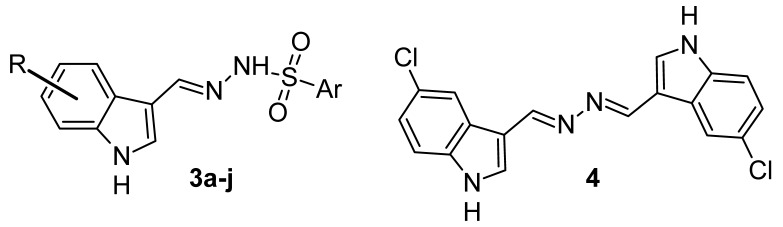					
Substituents in Ar	Model	LE MCF-7	SI MCF-7	LE MDA-MB-231	SI MDA-MB-231
	***Coefficients***				
	***b*** **_0_**	0.222	10.362	0.202	2.037
phenyl	***b*** **_1_**	0.037	6.401	0.037	1.464
4-methylphenyl	***b*** **_2_**	0.002	6.901	−0.002	−0.216
4-methoxyphenyl	***b*** **_3_**	−0.008	−6.274	−0.006	−0.359
2,4,6-trimethylphenyl	***b*** **_4_**	−0.016	−3.465	−0.014	0.244
2,4-dimethylphenyl	***b*** **_5_**	−0.019	−5.378	−0.007	−1.320
3,4-dimethylphenyl	***b*** **_6_**	−0.017	−3.573	−0.013	−0.325
5-chlorophenyl	***b*** **_7_**	−0.028	−4.151	−0.042	−2.180
Substituents in R					
1-acetyl	***b*** **_8_**	−0.004	18.918	−0.025	0.478
5-methoxy	***b*** **_9_**	0.020	−10.180	0.006	−1.587
5-bromo	***b*** **_10_**	−0.009	−8.353	0.003	−0.748
5-chloro	***b*** **_11_**	0.001	−3.702	0.015	0.957
	***R***	0.904	0.919	0.938	0.666
	***RMSE***	0.012	4.052	0.009	1.247
	***Q***	0.666	0.678	0.655	−0.272

## Data Availability

The original contributions presented in this study are included in the article/Supplementary Materials. Further inquiries can be directed to the corresponding author.

## Supplementary Materials

The following supporting information can be downloaded at https://www.mdpi.com/article/10.3390/ph18081231/s1. Figures S1–S74: Analytical data for the synthesized compounds; Table S1. Binary coded structures used an input for the QSAR analysis.

## Abbreviations

The following abbreviations are used in this manuscript:

ADME	Absorption, distribution, metabolism, and excretion
BA	Bioavailability
BBB	Blood–brain barier
BC	Breast cancer
CYP	Cytochrome P450 enzymes
ER+	Estrogen receptor-positive breast cancer
LE	Ligand efficiency
MDR	Multidrug resistance
MLR	Multiple linear regression
QSAR	Quantitative structure–activity relationship
QSPkR	Quantitative structure–pharmacokinetics relationship
SI	Similarity index
TNBC	Triple-negative breast cancer
