# Supplementary material for: Novel Indole-Based Sulfonylhydrazones as Potential Anti-Breast Cancer Agents: Synthesis, In Vitro Evaluation, ADME, and QSAR Studies
Source: Pharmaceuticals (Basel). 2025 Aug 20;18(8):1231. doi: 10.3390/ph18081231 (PMC12389196; doi:10.3390/ph18081231)
Supplement: Supplementary file 1 [file pharmaceuticals-18-01231-s001.zip › pharmaceuticals-3779167-supplementary.pdf]

Supplementary Materials

Novel Indole-Based Sulfonylhydrazones as Potential Anti-Breast Cancer Agents: Synthesis, In Vitro Evaluation, ADME, and QSAR Studies

**Violina T. Angelova [1,*], Rositsa Mihaylova [1], Zvetanka Zhivkova [1], Nikolay Vassilev [2], Boris Shivachev [3]
and Irini Doytchinova [1]**

1 Faculty of Pharmacy, Medical University of Sofia, 1000 Sofia, Bulgaria; rmihaylova@pharmfac.mu-sofia.bg (R.M.); zzhikova@pharmfac.mu-sofia.bg (Z.Z.); idoytchinova@pharmfac.mu-sofia.bg (I.D.)
2 Laboratory "Nuclear Magnetic Resonance", Institute of Organic Chemistry with Centre of Phytochemistry, Bulgarian Academy of Sciences, 1113 Sofia, Bulgaria; nikolay.vassilev@orgchm.bas.bg
3 Institute of Mineralogy and Crystallography "Acad. Ivan Kostov", Bulgarian Academy of Sciences,
1113 Sofia, Bulgaria; blshivachev@gmail.com
* Correspondence: v.stoyanova@pharmfac.mu-sofia.bg

^{1}H NMR, ^{13}C NMR, DEPT-135 NMR, 2D COSY NMR, 2D HSQC NMR, 2D HMBC NMR and HRMS spectra (Figures S1-S72)

Binary coded structures of the indolyl-methylidene substituted phenylsulfonylhydrazones used in the QSAR study - Table S1.

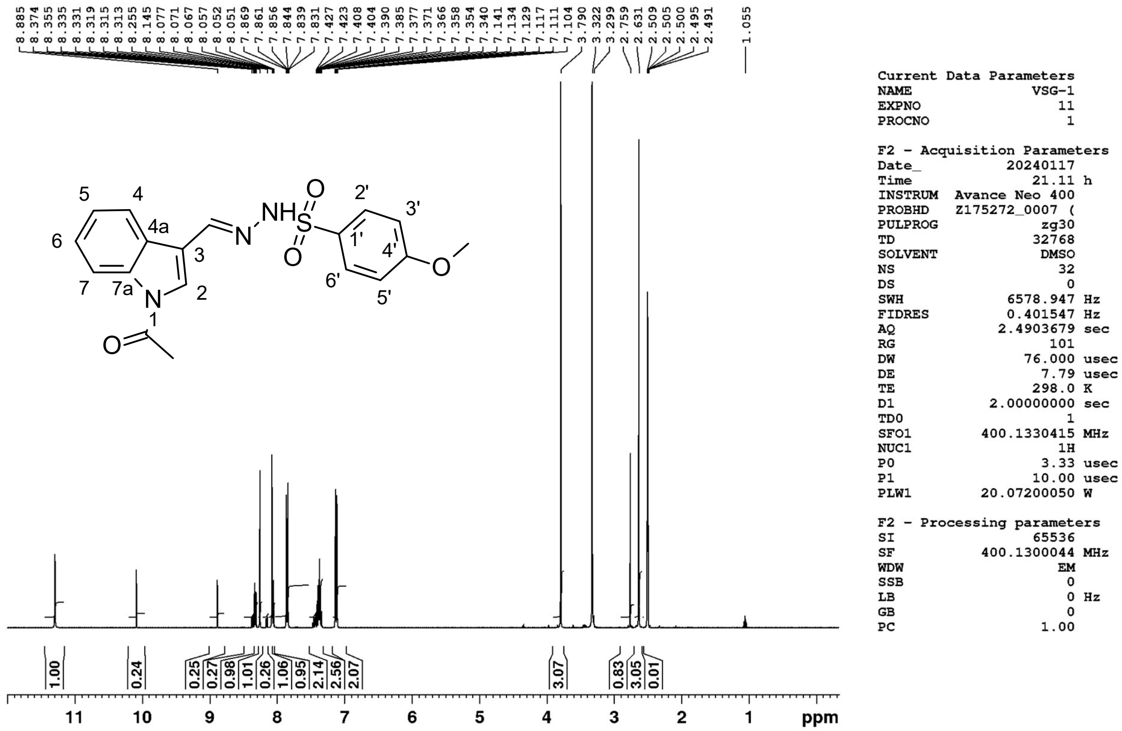

Figure 1S. ^{1}H NMR spectrum of *N'*-[(*E*)-(1-acetyl-1*H*-indol-3-yl)methylidene]-4-methoxybenzene-1-sulfonohydrazide, **1** in DMSO-d_6

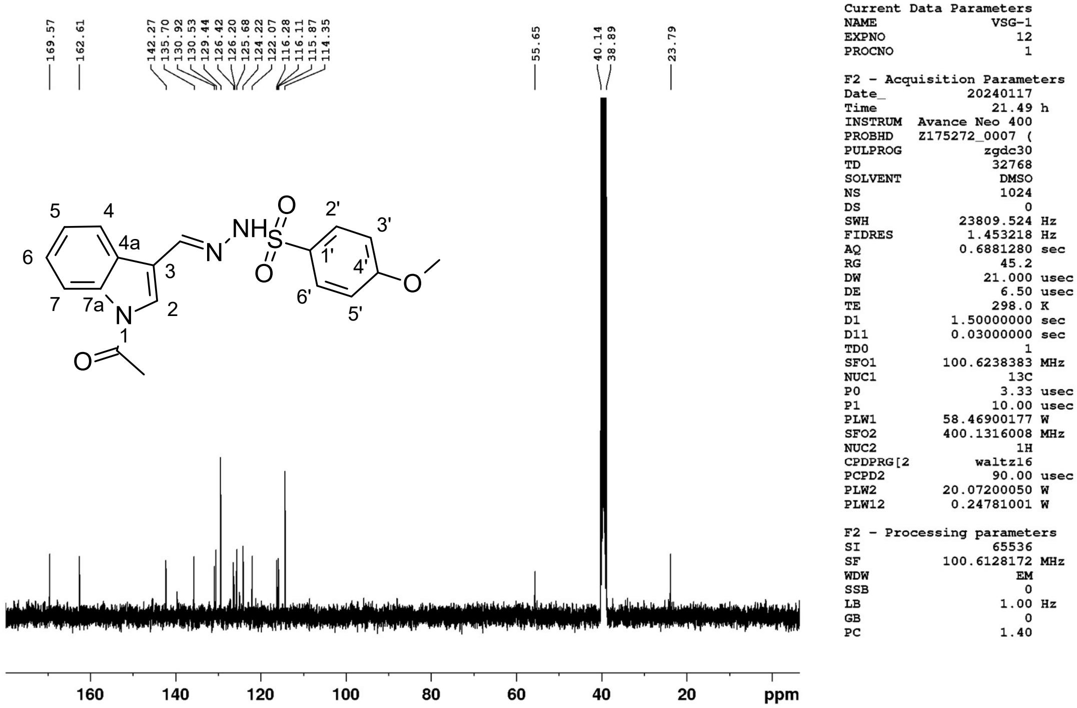

Figure 2S. ^{13}C NMR spectrum of *N'*-[(*E*)-(1-acetyl-1*H*-indol-3-yl)methylidene]-4-methoxybenzene-1-sulfonohydrazide, **1** in DMSO-d_6

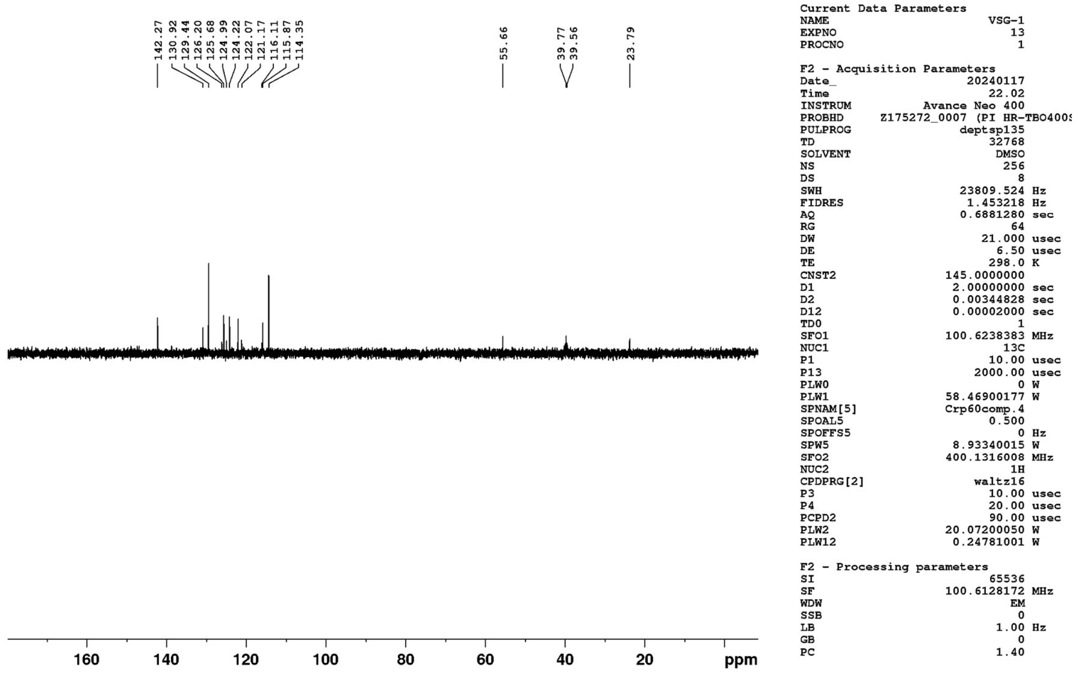

Figure 3S. DEPT-135 NMR spectrum of *N'*-[(*E*)-(1-acetyl-1*H*-indol-3-yl)methylidene]-4-methoxybenzene-1-sulfonohydrazide, **1** in DMSO-d_6

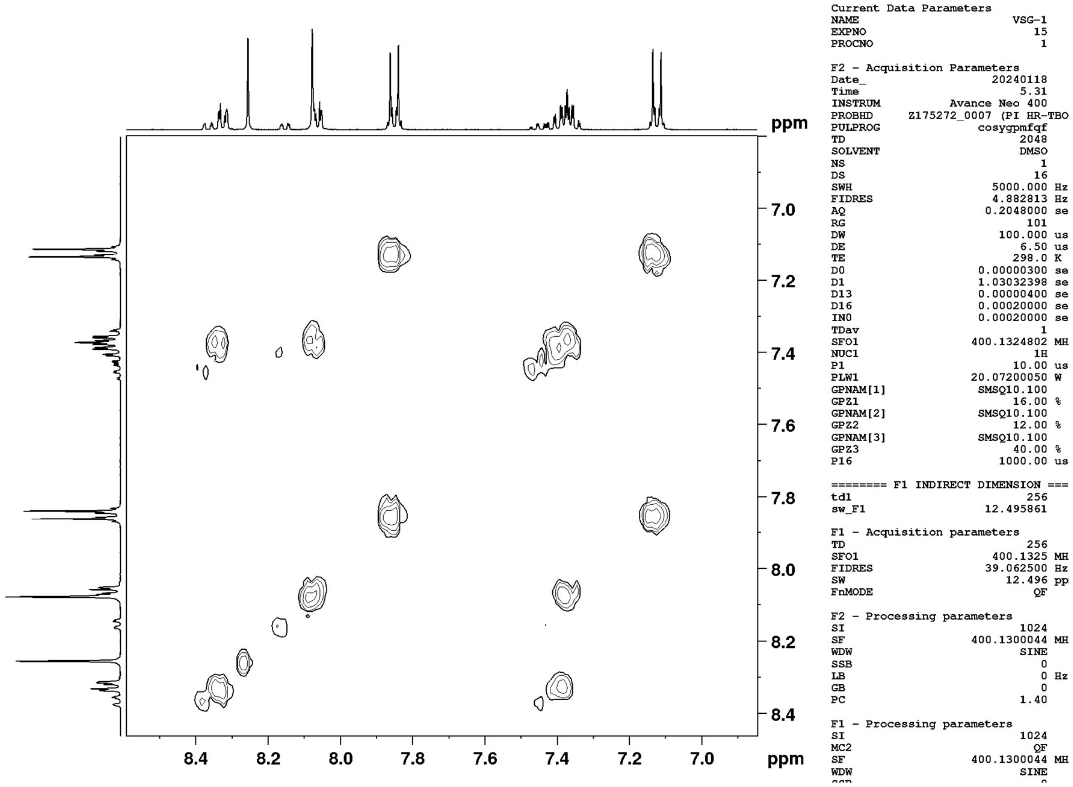

Figure 4S. 2D COSY NMR spectrum of *N'*-[(*E*)-(1-acetyl-1*H*-indol-3-yl)methylidene]-4-methoxybenzene-1-sulfonohydrazide, **1** in DMSO-d_6

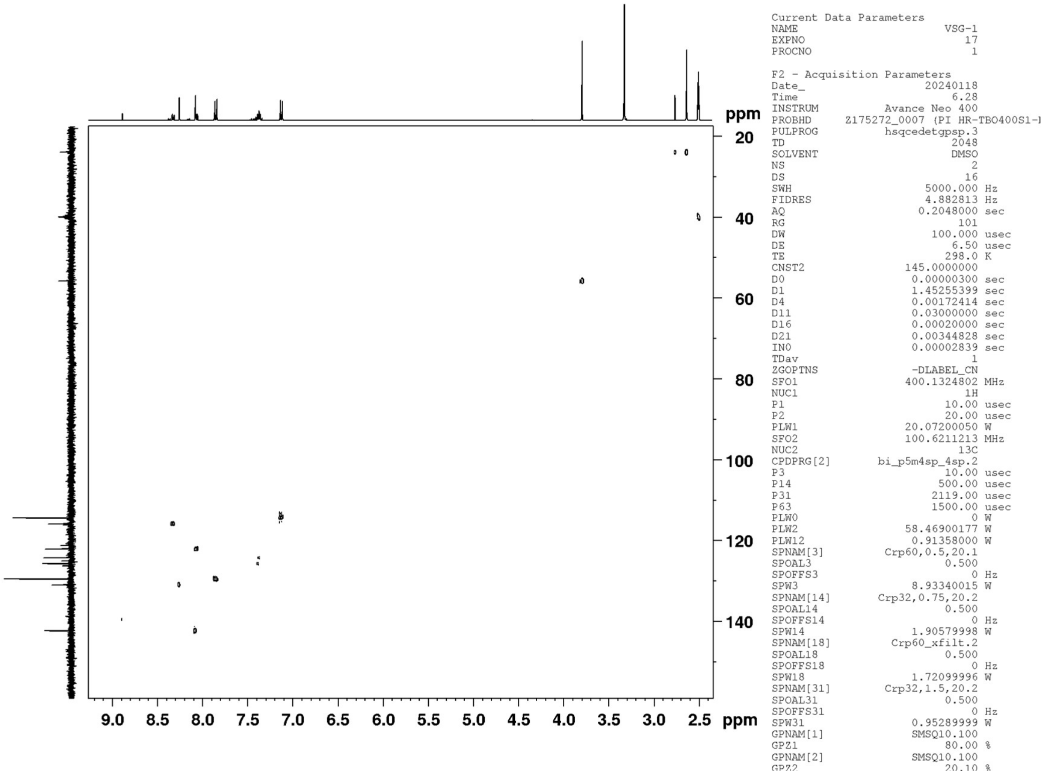

Figure 5S. 2D HSQC NMR spectrum of *N*'-[(*E*)-(1-acetyl-1*H*-indol-3-yl)methylidene]-4-methoxybenzene-1-sulfonohydrazide, **1** in DMSO-d_6

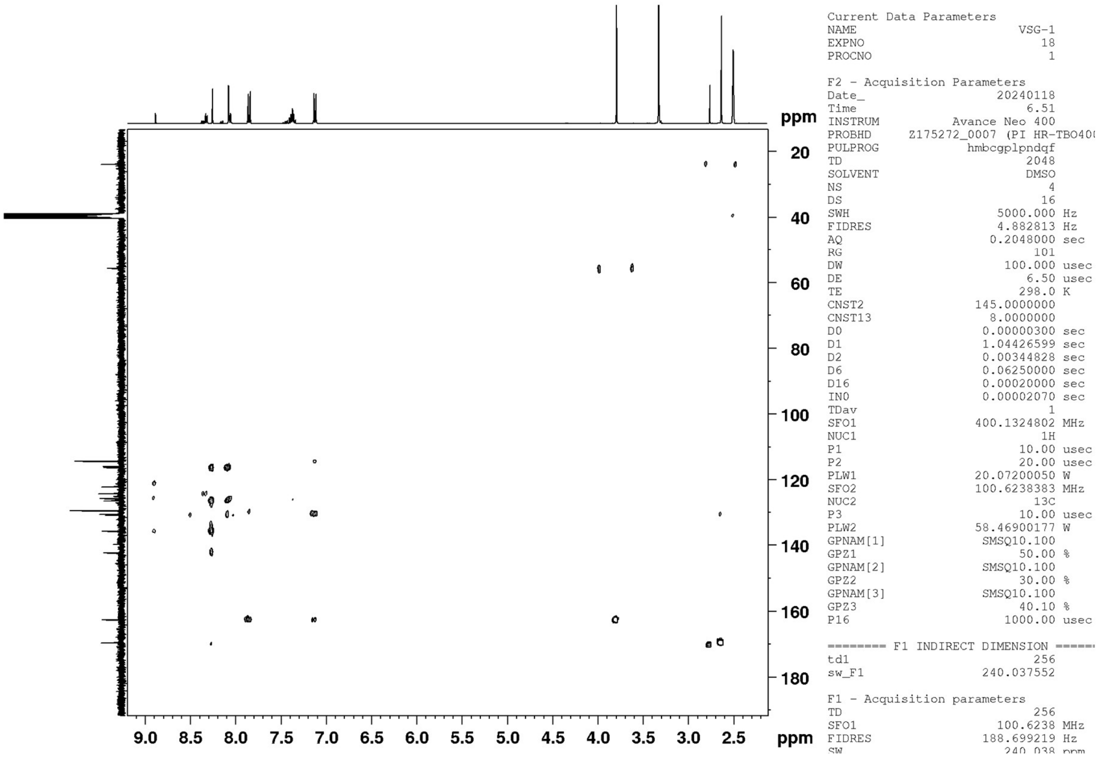

Figure 6S. 2D HMBC NMR spectrum of *N*'-[(*E*)-(1-acetyl-1*H*-indol-3-yl)methylidene]-4-methoxybenzene-1-sulfonohydrazide, **1** in DMSO-d_6

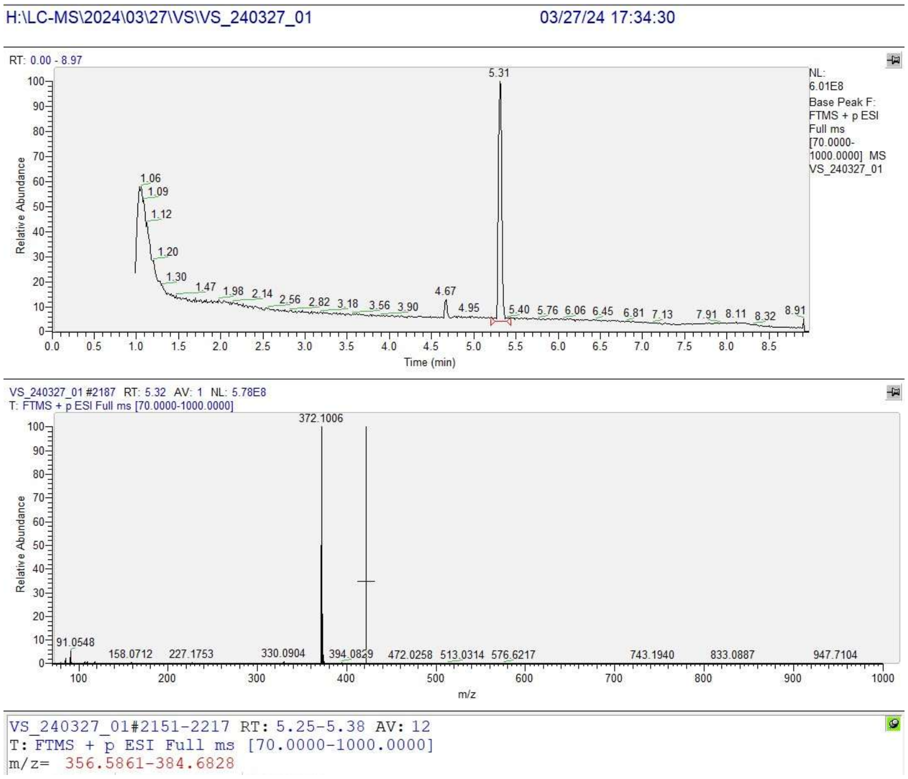

Figure 7S. HRMS of *N*'-[(*E*)-(1-acetyl-1*H*-indol-3-yl)methylidene]-4-methoxybenzene-1-sulfonohydrazide, **1**

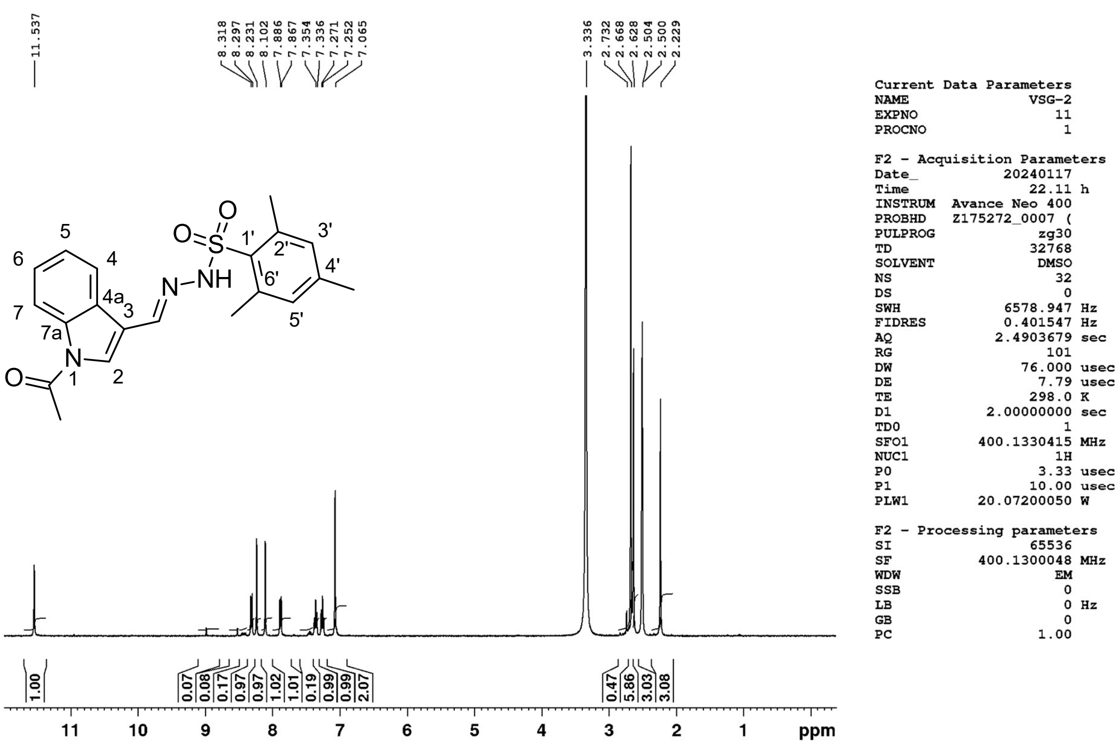

Figure 8S. ^{1}H NMR spectrum of *N*'-[(*E*)-(1-acetyl-1*H*-indol-3-yl)methylidene]-2,4,6-trimethylbenzene-1-sulfonohydrazide, **2** in DMSO-d_6

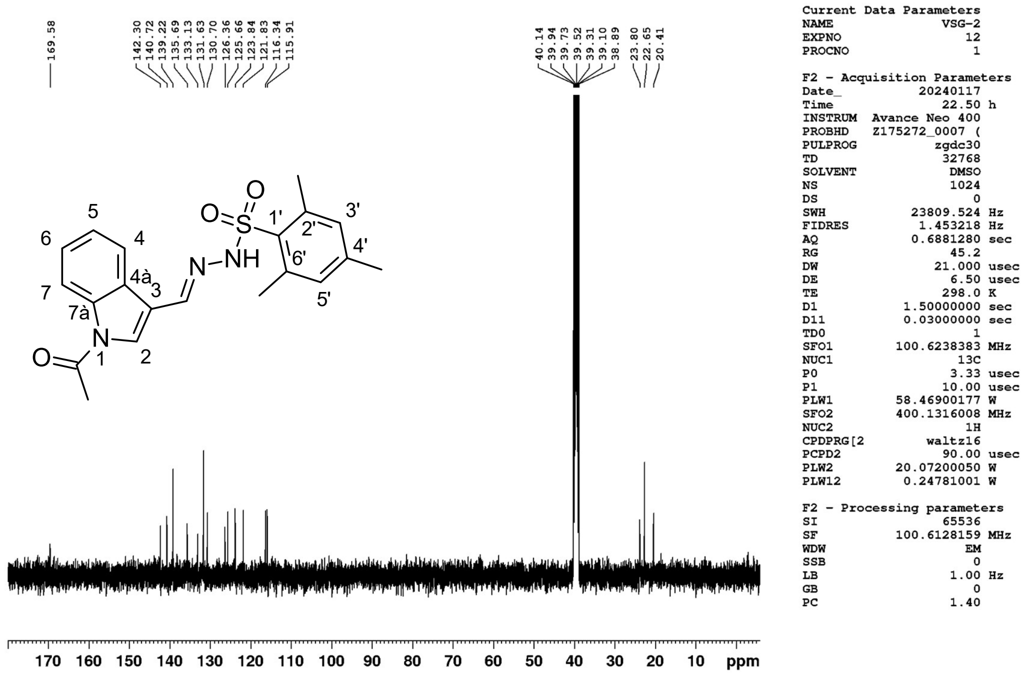

Figure 9S. ^{13}C NMR spectrum of *N'*-[(*E*)-(1-acetyl-1*H*-indol-3-yl)methylidene]-2,4,6-trimethylbenzene-1-sulfonohydrazide, **2** in DMSO-d_6

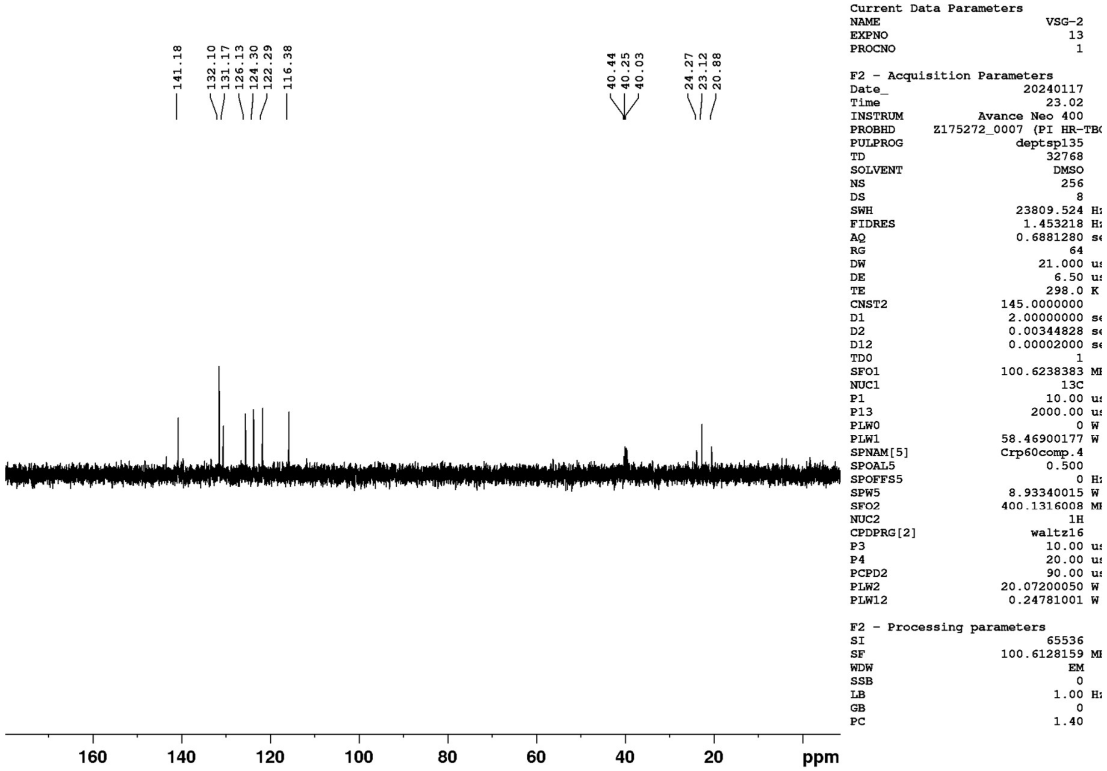

Figure 10S. DEPT-135 NMR spectrum of *N'*-[(*E*)-(1-acetyl-1*H*-indol-3-yl)methylidene]-2,4,6-trimethylbenzene-1-sulfonohydrazide, **2** in DMSO-d_6

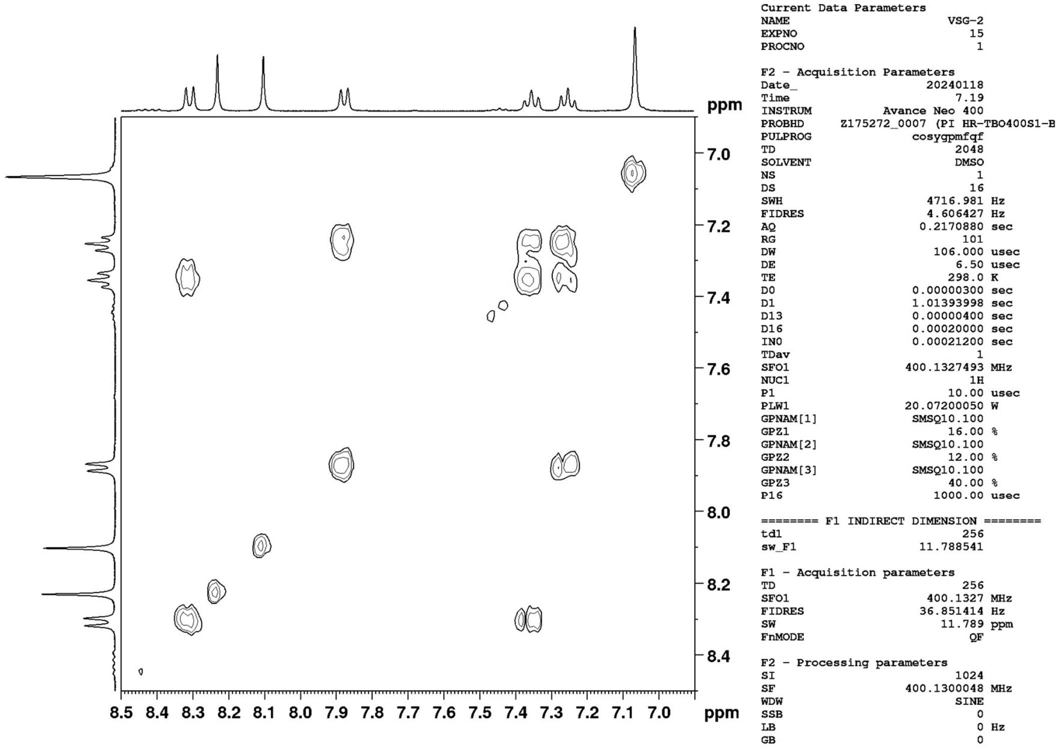

Figure 11S. 2D COSY NMR spectrum of *N'*-[(*E*)-(1-acetyl-1*H*-indol-3-yl)methylidene]-2,4,6-trimethylbenzene-1-sulfonohydrazide, **2** in DMSO-d_6

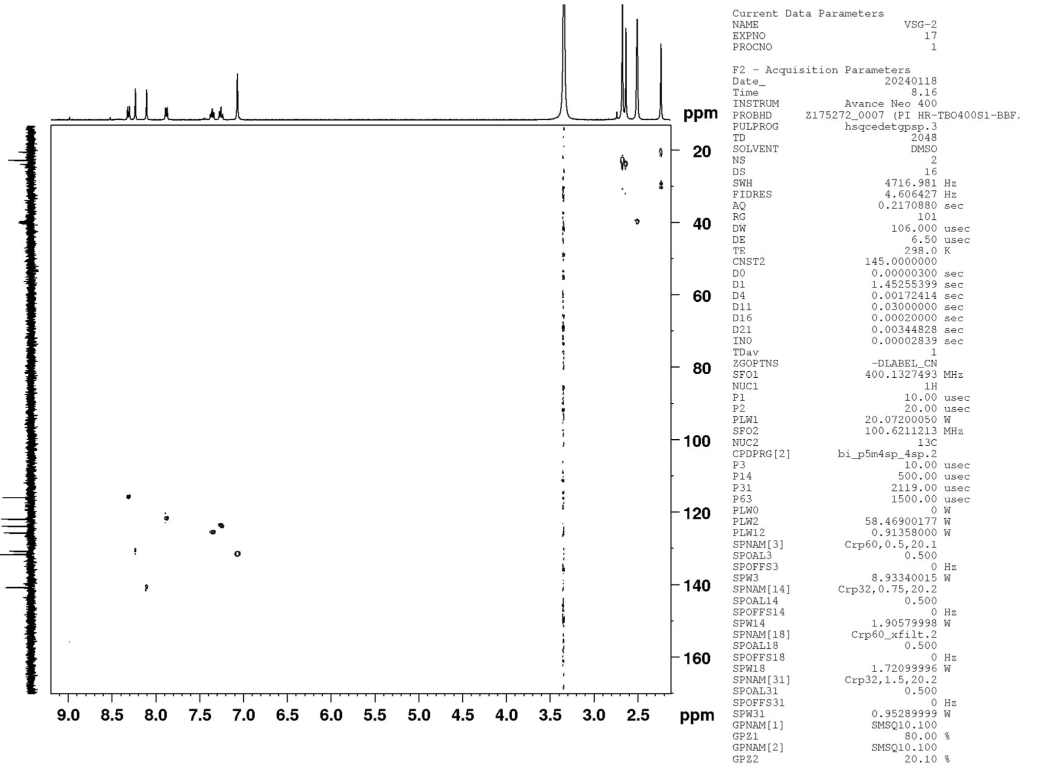

Figure 12S. 2D HSQC NMR spectrum of *N'*-[(*E*)-(1-acetyl-1*H*-indol-3-yl)methylidene]-2,4,6-trimethylbenzene-1-sulfonohydrazide, **2** in DMSO-d_6

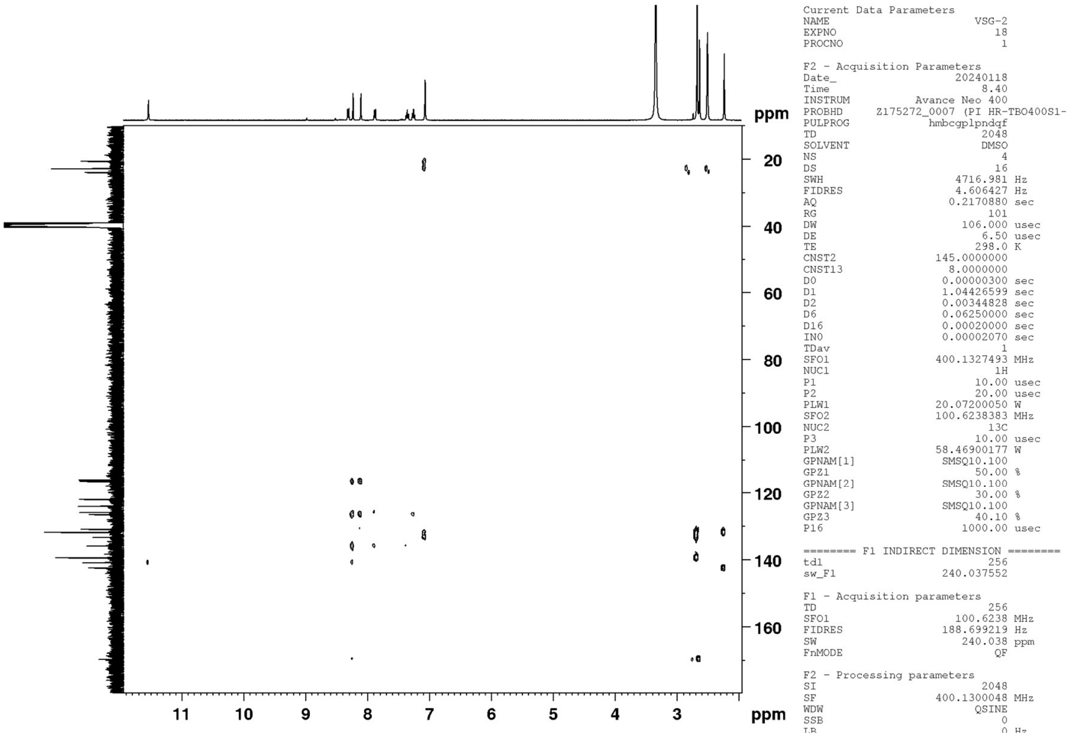

Figure 13S. 2D HMBC NMR spectrum of *N'*-[(*E*)-(1-acetyl-1*H*-indol-3-yl)methylidene]-2,4,6-trimethylbenzene-1-sulfonohydrazide, **2** in DMSO-d_6

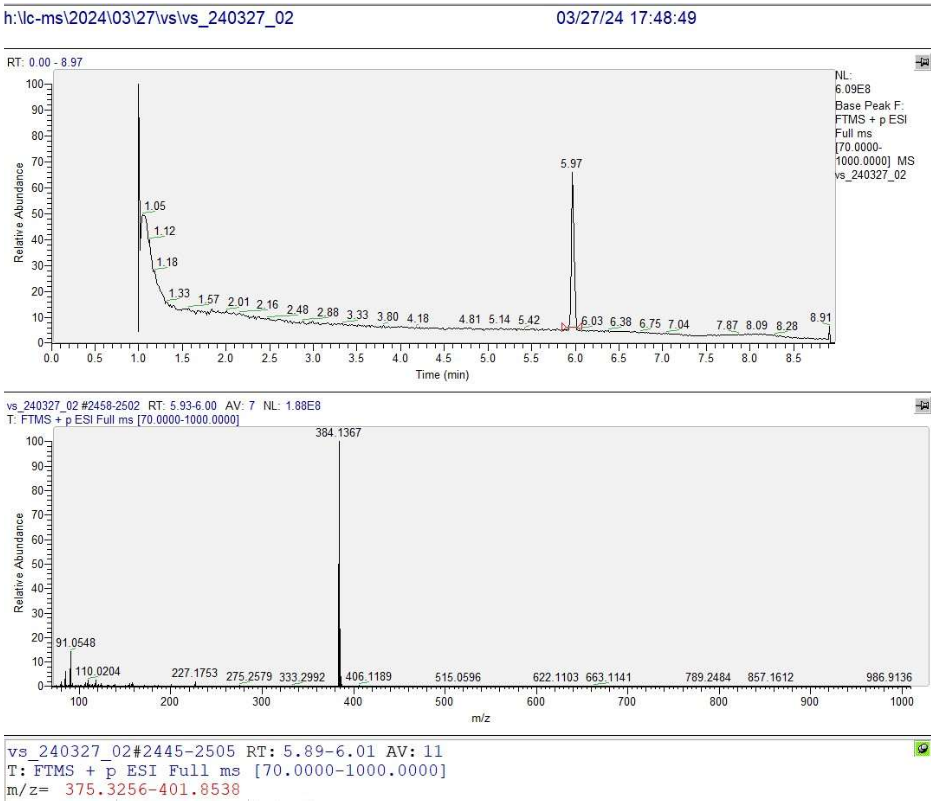

Figure 14S. HRMS of *N'*-[(*E*)-(1-acetyl-1*H*-indol-3-yl)methylidene]-2,4,6-trimethylbenzene-1-sulfonohydrazide, **2**

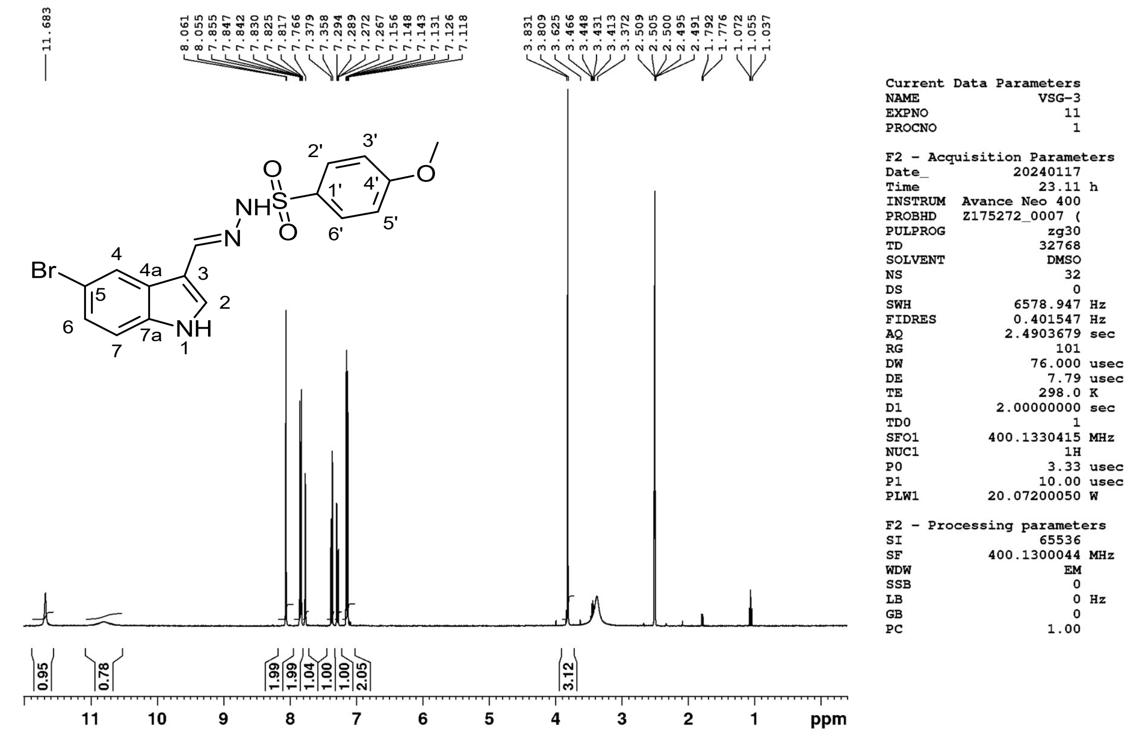

Figure 15S. ^{1}H NMR spectrum of *N'*-[(*E*)-(5-bromo-1*H*-indol-3-yl)methylidene]-4-methoxybenzene-1-sulfonohydrazide, **3** in DMSO-*d*$_6$

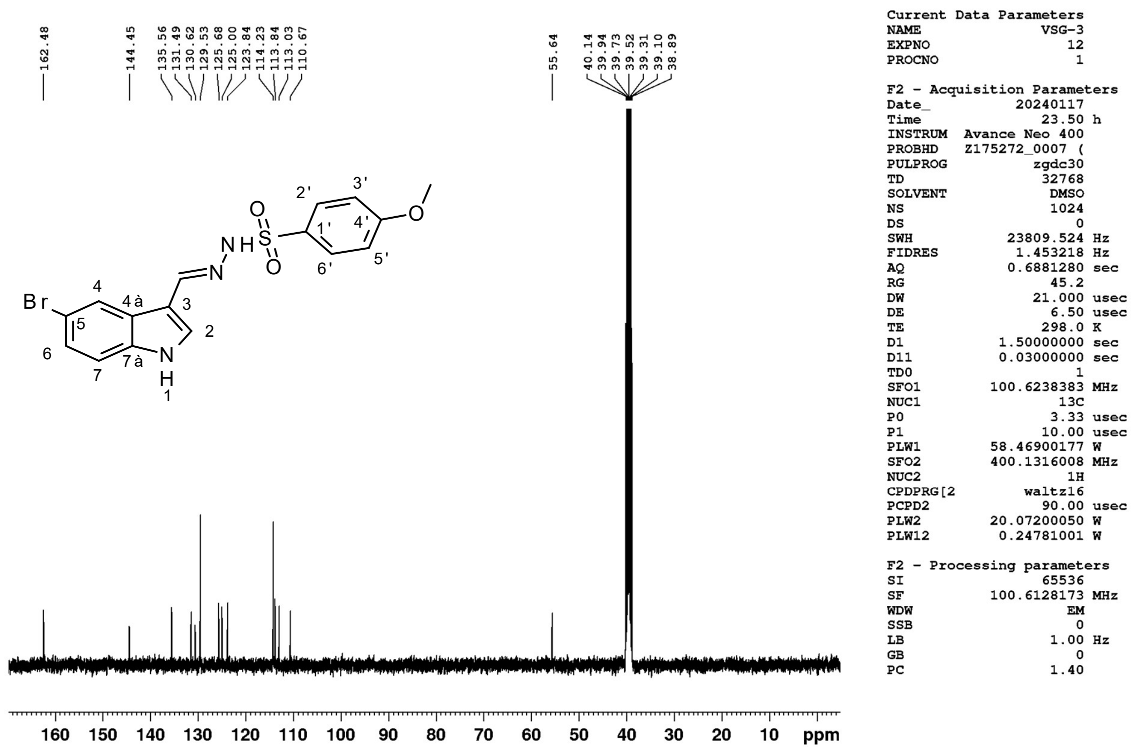

Figure 16S. ^{13}C NMR spectrum of *N'*-[(*E*)-(5-bromo-1*H*-indol-3-yl)methylidene]-4-methoxybenzene-1-sulfonohydrazide, **3** in DMSO-*d*$_6$

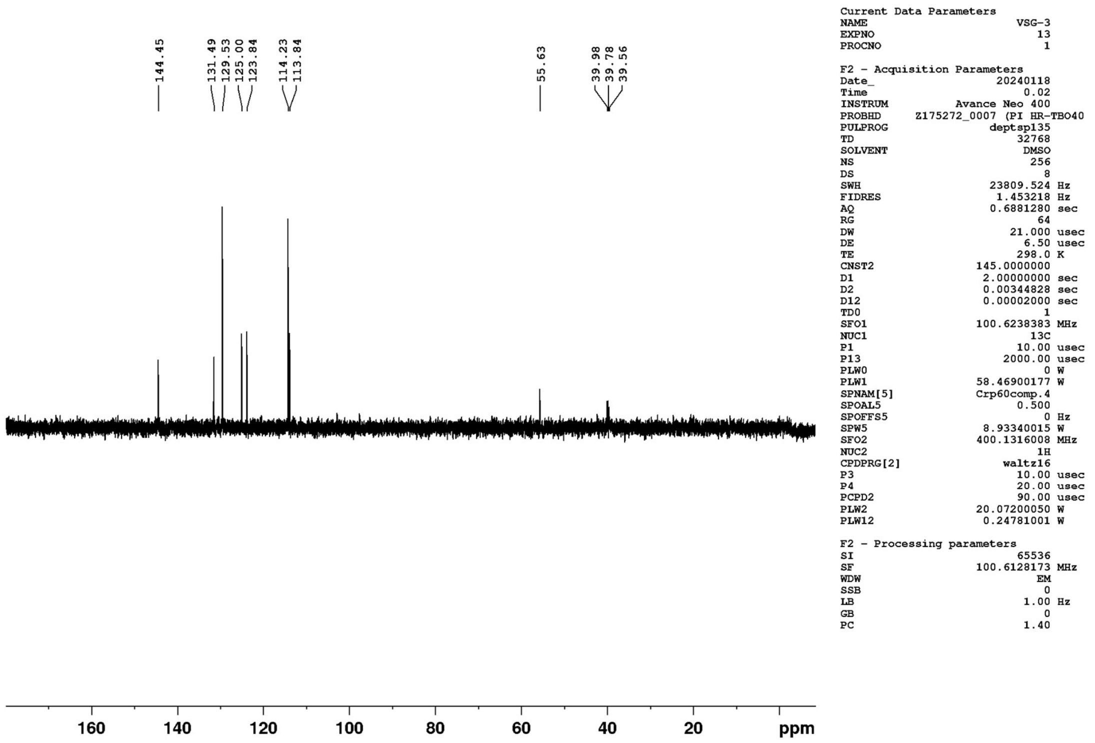

Figure 17S. DEPT-135 NMR spectrum of *N'*-[(*E*)-(5-bromo-1*H*-indol-3-yl)methylidene]-4-methoxybenzene-1-sulfonohydrazide, **3** in DMSO-d_6

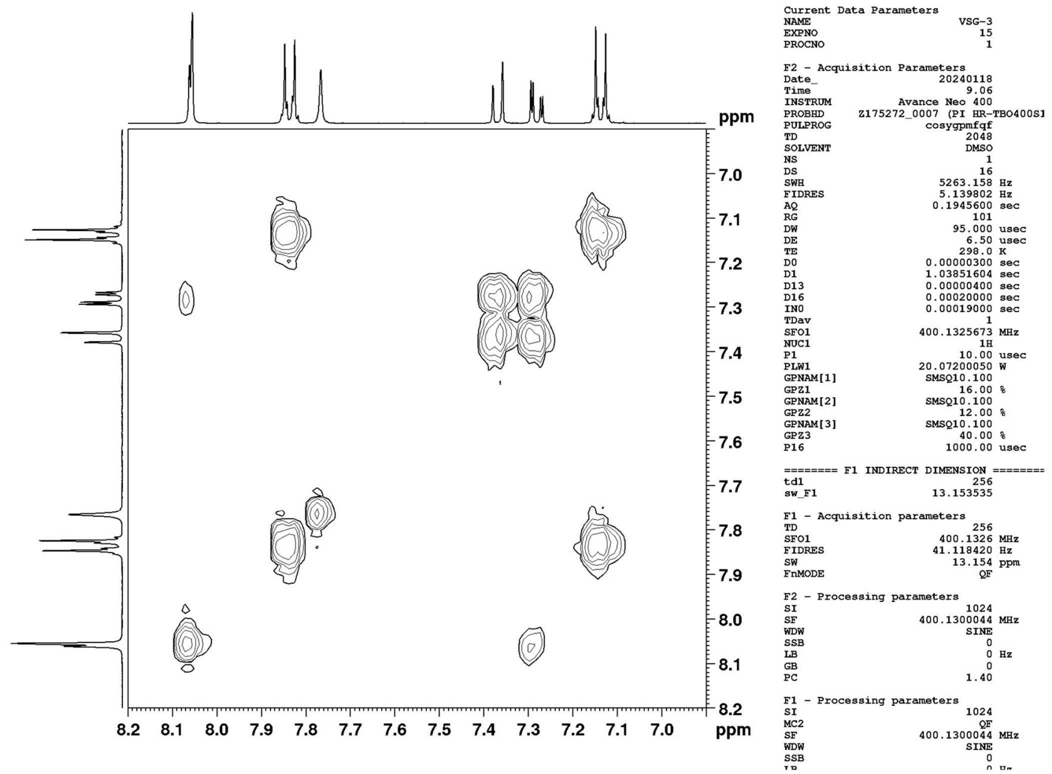

Figure 18S. 2D COSY NMR spectrum of *N'*-[(*E*)-(5-bromo-1*H*-indol-3-yl)methylidene]-4-methoxybenzene-1-sulfonohydrazide, **3** in DMSO-d_6

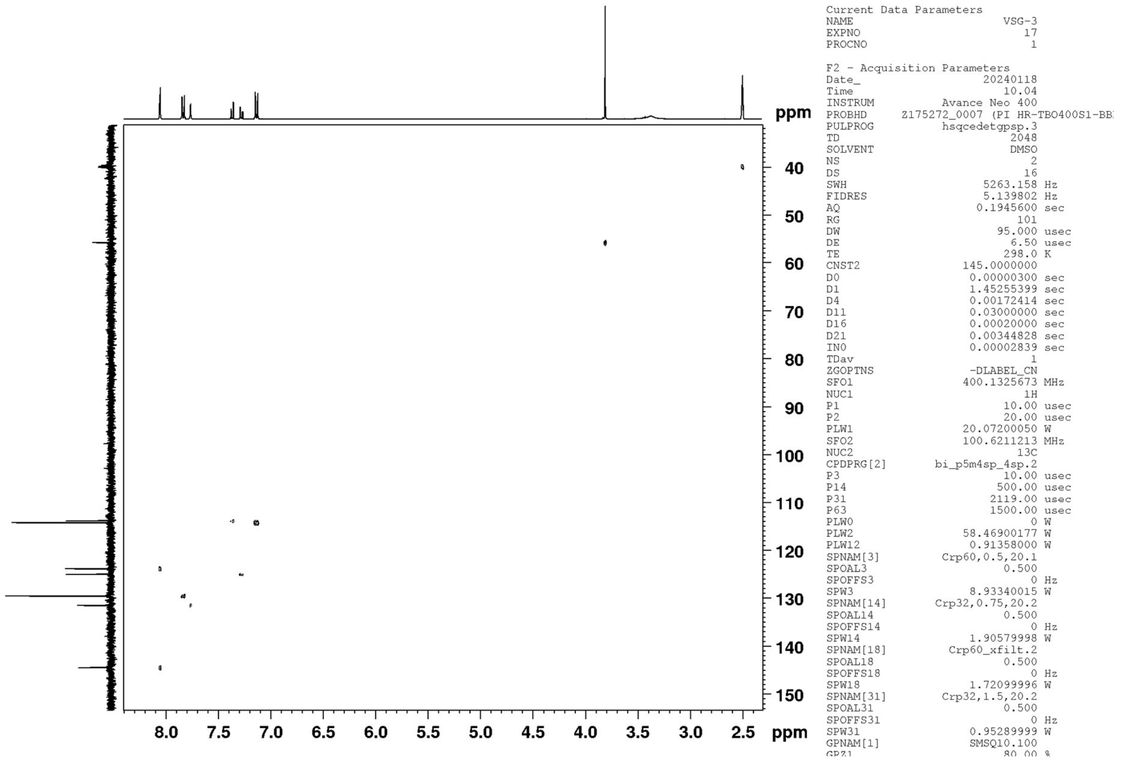

Figure 19S. 2D HSQC NMR spectrum of *N'*-[(*E*)-(5-bromo-1*H*-indol-3-yl)methylidene]-4-methoxybenzene-1-sulfonohydrazide, **3** in DMSO-d_6

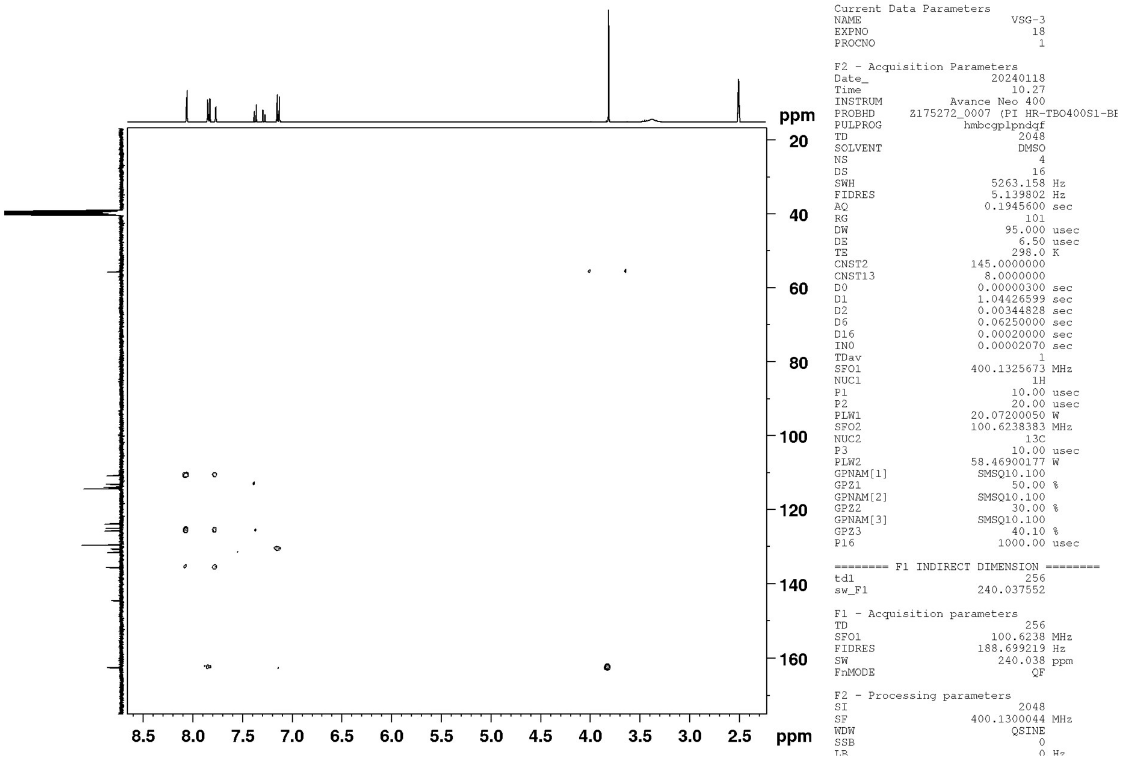

Figure 20S. 2D HMBC NMR spectrum of *N'*-[(*E*)-(5-bromo-1*H*-indol-3-yl)methylidene]-4-methoxybenzene-1-sulfonohydrazide, **3** in DMSO-d_6

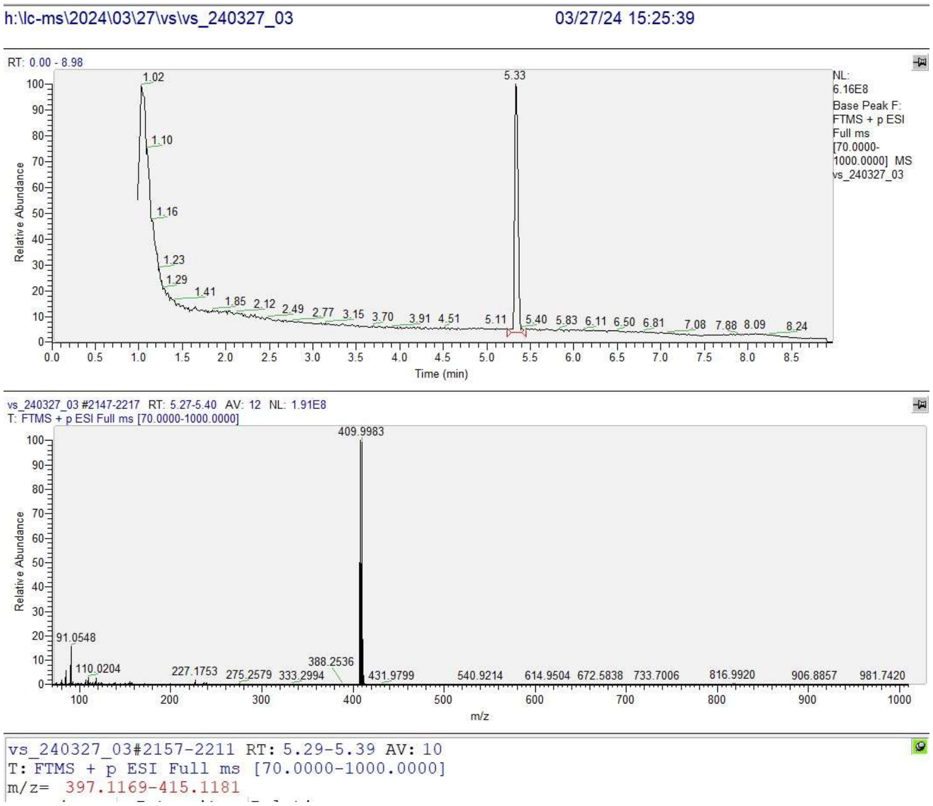

Figure 21S. HRMS of *N'*-[(*E*)-(5-bromo-1*H*-indol-3-yl)methylidene]-4-methoxybenzene-1-sulfonohydrazide, **3**

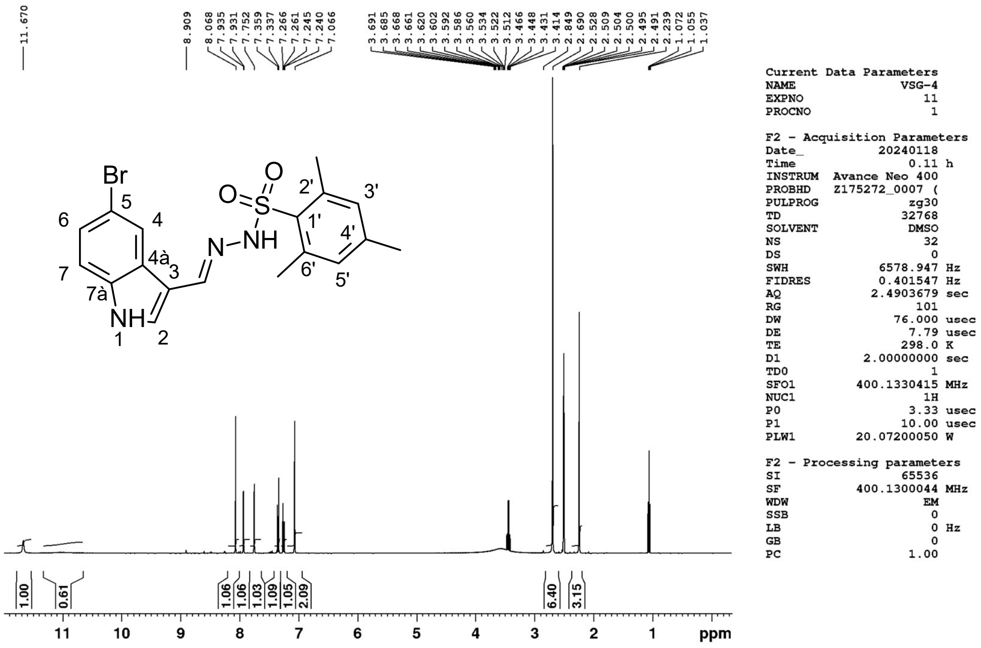

Figure 22S. ^{1}H NMR spectrum of *N'*-[(*E*)-(5-bromo-1*H*-indol-3-yl)methylidene]-2,4,6-trimethylbenzene-1-sulfonohydrazide, **4** in DMSO-d_6

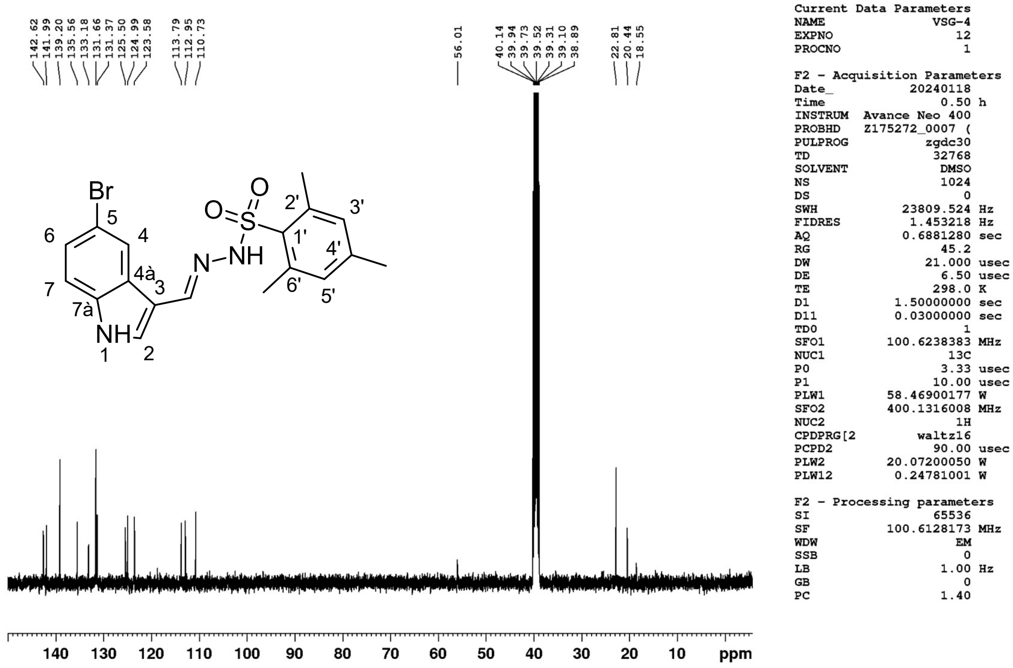

Figure 23S. ^{13}C NMR spectrum of *N*'-[(*E*)-(5-bromo-1*H*-indol-3-yl)methylidene]-2,4,6-trimethylbenzene-1-sulfonohydrazide, **4** in DMSO-d_6

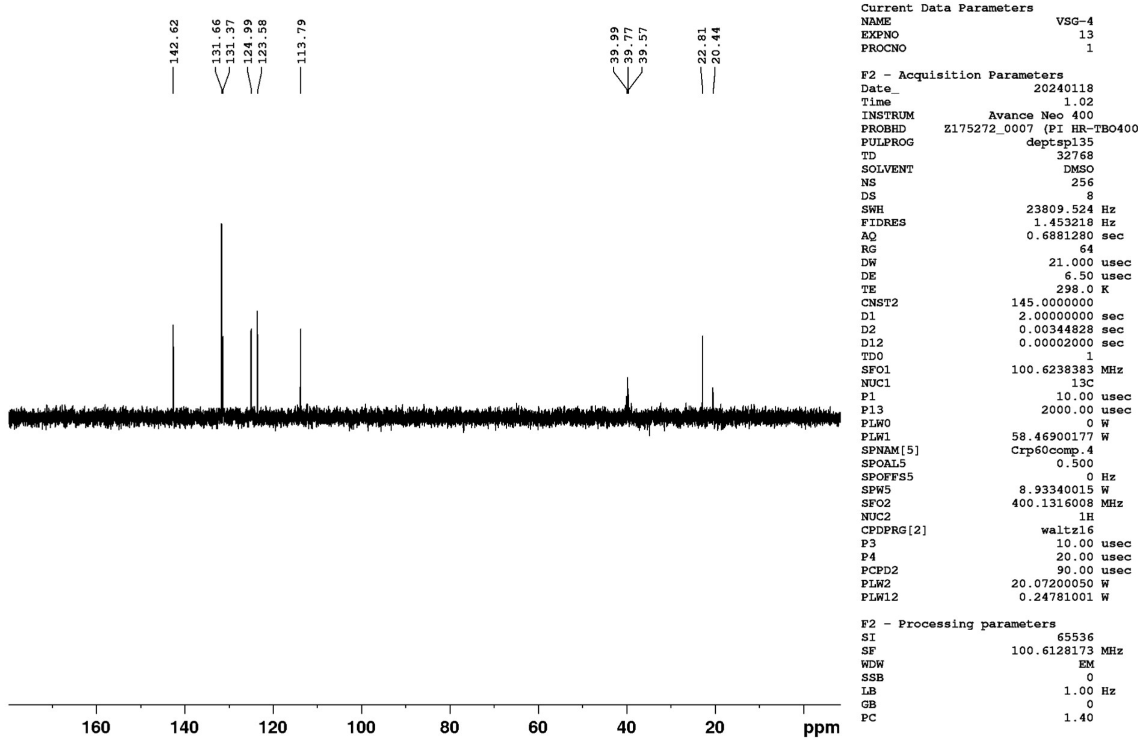

Figure 24S. DEPT-135 NMR spectrum of *N*'-[(*E*)-(5-bromo-1*H*-indol-3-yl)methylidene]-2,4,6-trimethylbenzene-1-sulfonohydrazide, **4** in DMSO-d_6

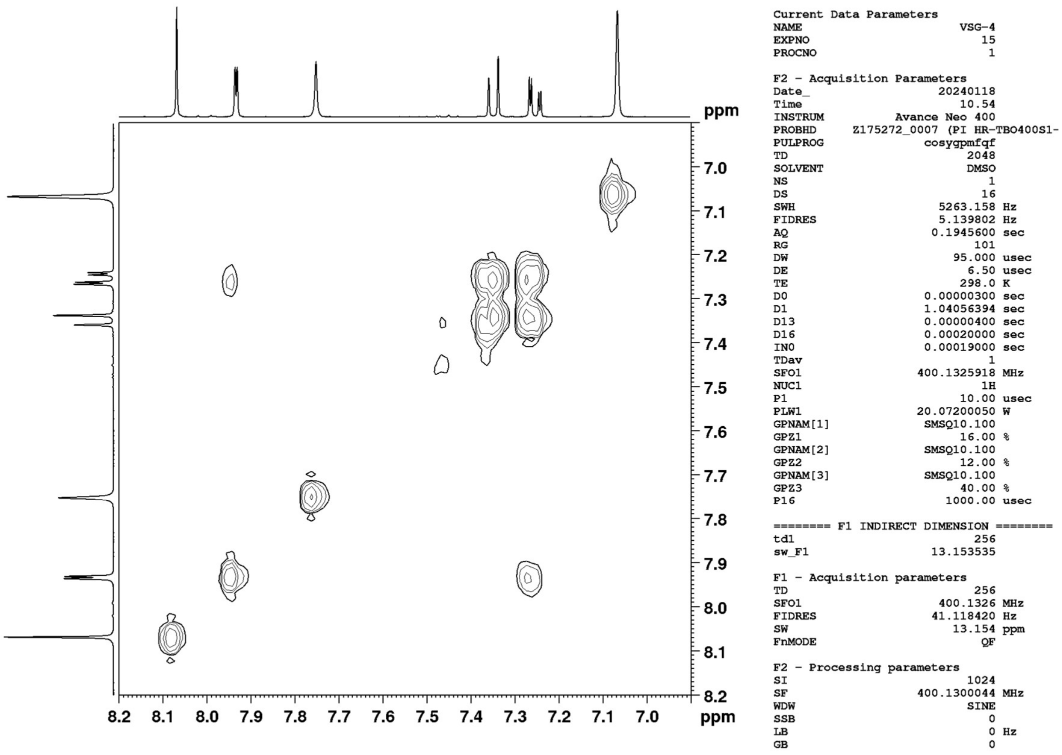

Figure 25S. 2D COSY NMR spectrum of *N'*-[(*E*)-(5-bromo-1*H*-indol-3-yl)methylidene]-2,4,6-trimethylbenzene-1-sulfonohydrazide, **4** in DMSO-d_6

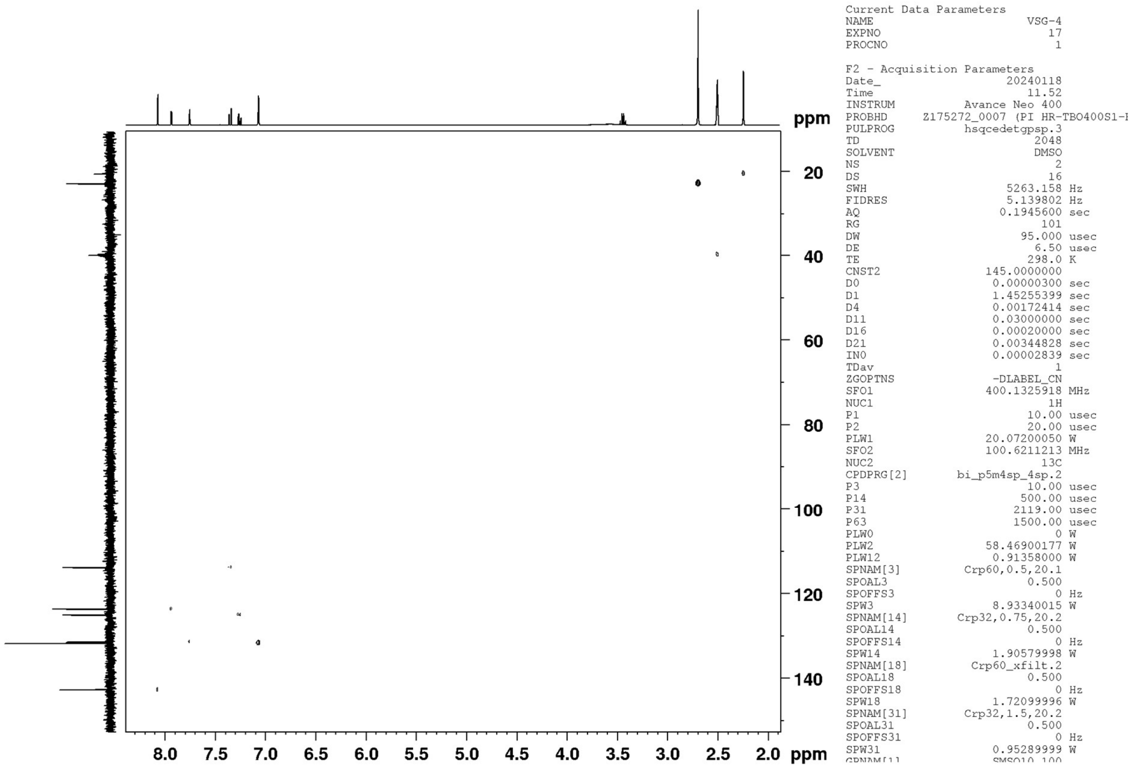

Figure 26S. 2D HSQC NMR spectrum of *N'*-[(*E*)-(5-bromo-1*H*-indol-3-yl)methylidene]-2,4,6-trimethylbenzene-1-sulfonohydrazide, **4** in DMSO-d_6

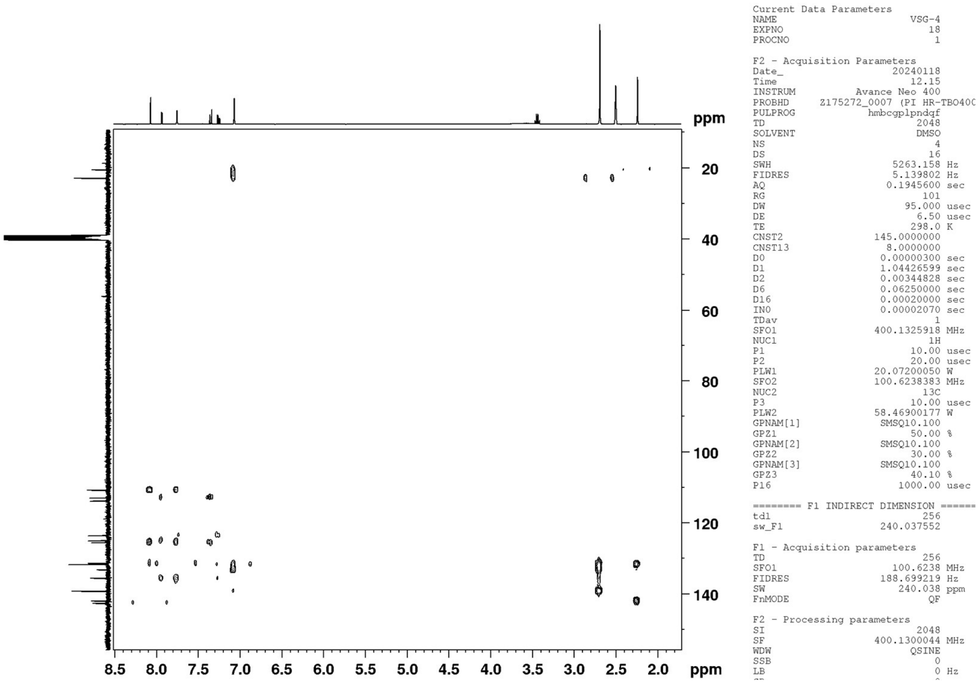

Figure 27S. 2D HMBC NMR spectrum of N'-[(E)-(5-bromo-1H-indol-3-yl)methylidene]-2,4,6-trimethylbenzene-1-sulfonohydrazide, **4** in DMSO-d_6

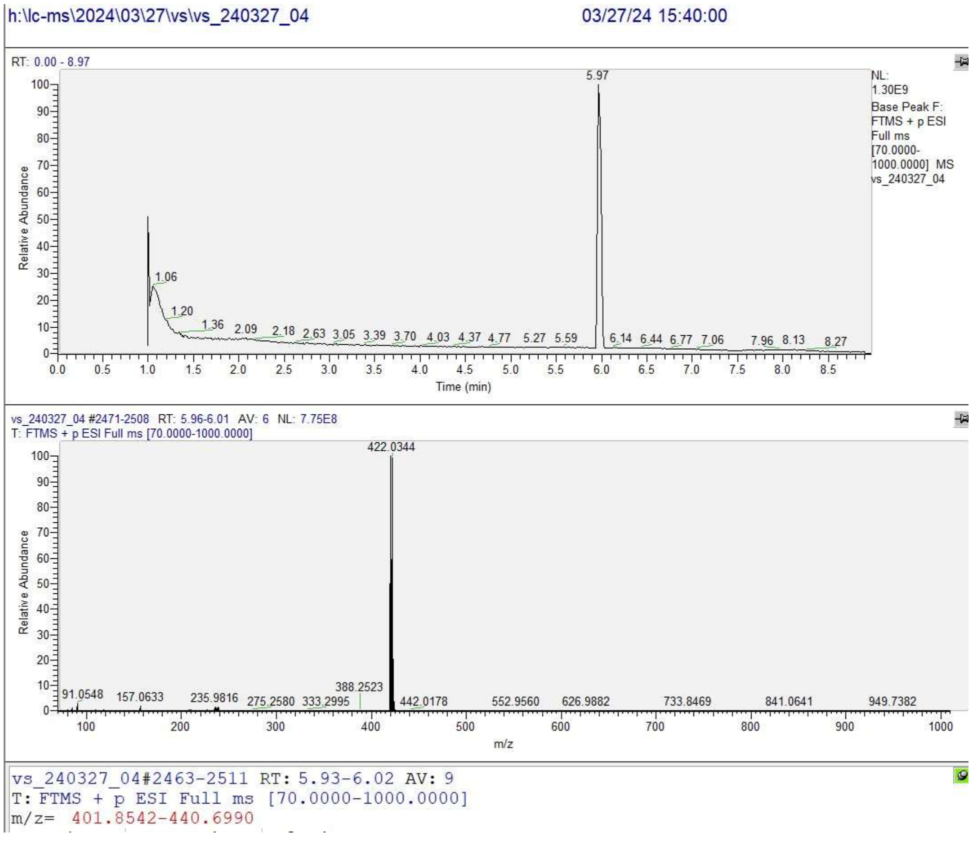

Figure 28S. HRMS of N'-[(E)-(5-bromo-1H-indol-3-yl)methylidene]-2,4,6-trimethylbenzene-1-sulfonohydrazide, **4**

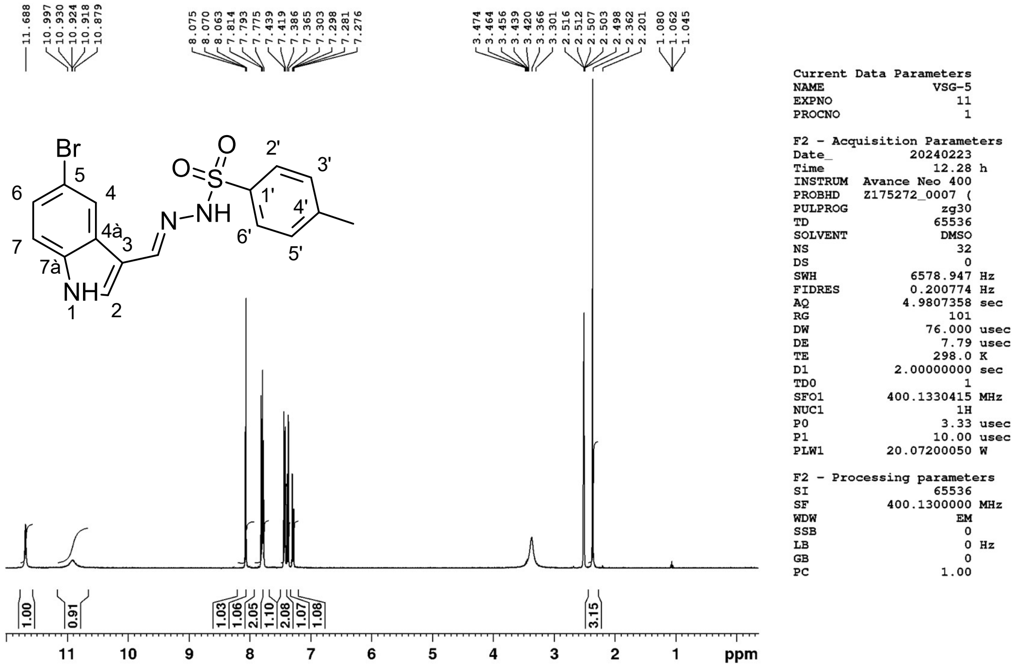

Figure 29S. ^{1}H NMR spectrum of *N*'-[(*E*)-(5-bromo-1*H*-indol-3-yl)methylidene]-4-methylbenzene-1-sulfonohydrazide, **5** in DMSO-d_6

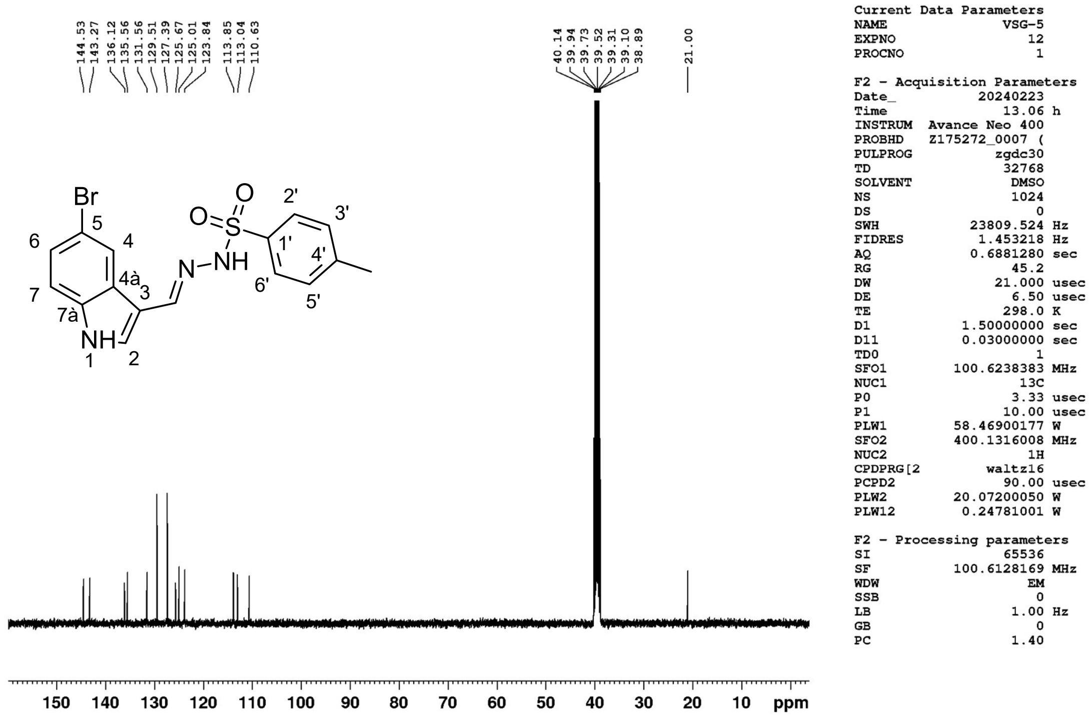

Figure 30S. ^{13}C NMR spectrum of *N*'-[(*E*)-(5-bromo-1*H*-indol-3-yl)methylidene]-4-methylbenzene-1-sulfonohydrazide, **1e** in DMSO-d_6

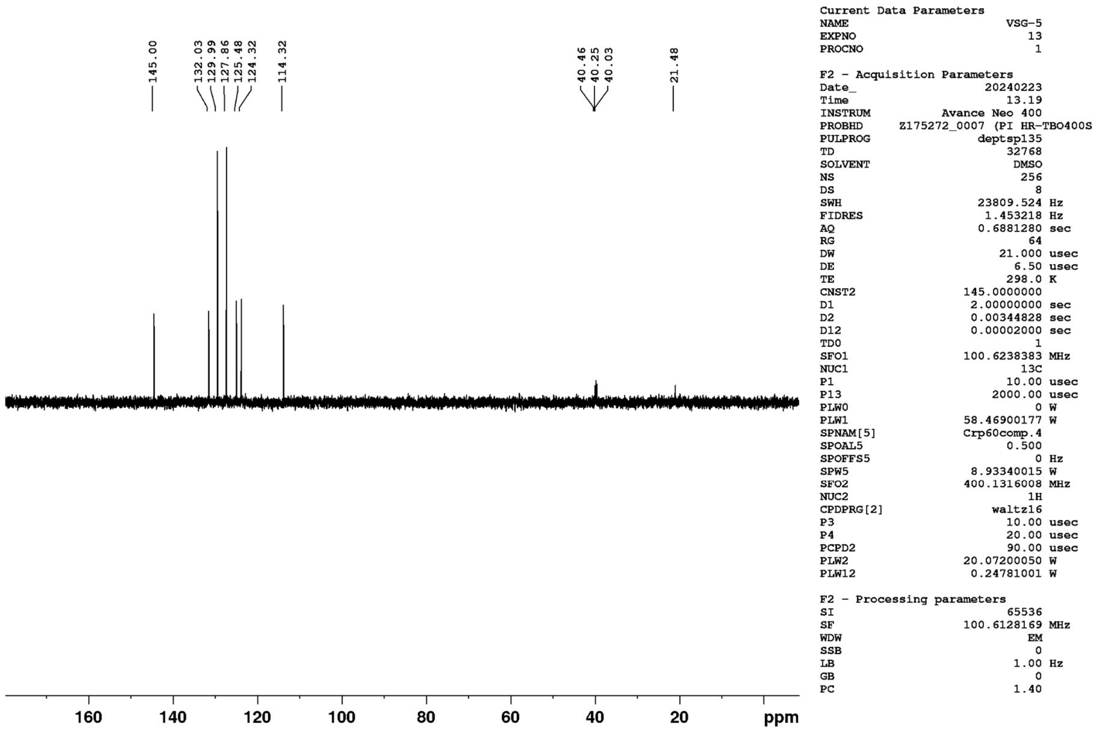

Figure 31S. DEPT-135 NMR spectrum of *N'*-[(*E*)-(5-bromo-1*H*-indol-3-yl)methylidene]-4-methylbenzene-1-sulfonohydrazide, **1e** in DMSO-d_6

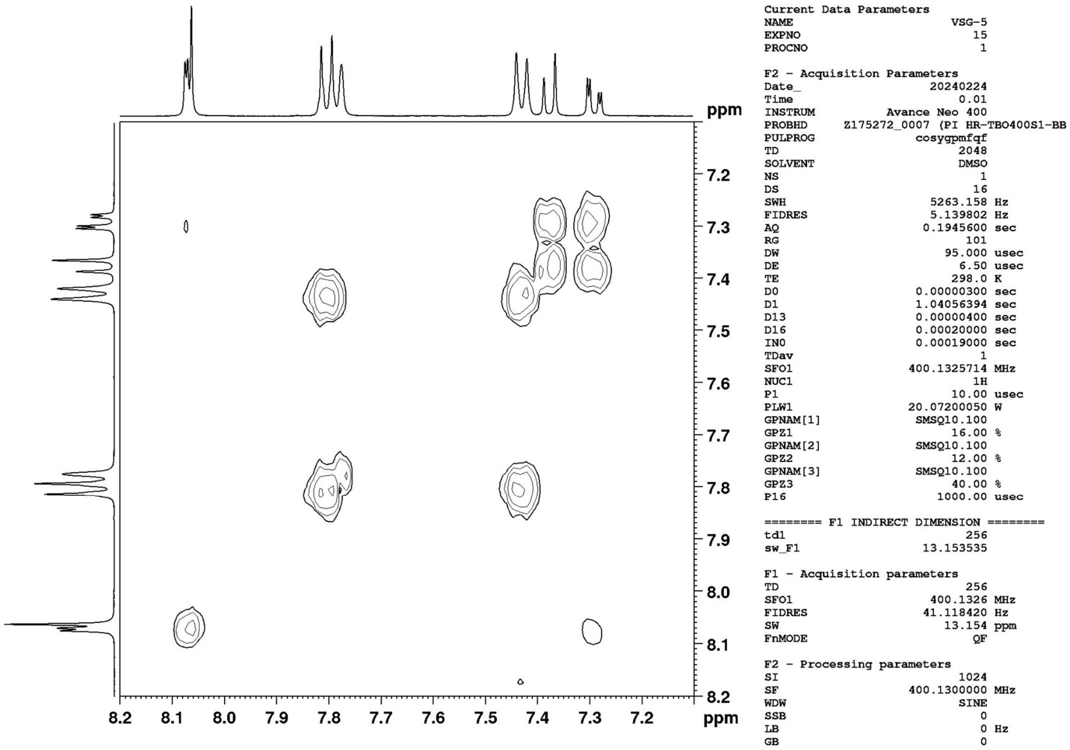

Figure 32S. 2D COSY NMR spectrum of *N'*-[(*E*)-(5-bromo-1*H*-indol-3-yl)methylidene]-4-methylbenzene-1-sulfonohydrazide, **1e** in DMSO-d_6

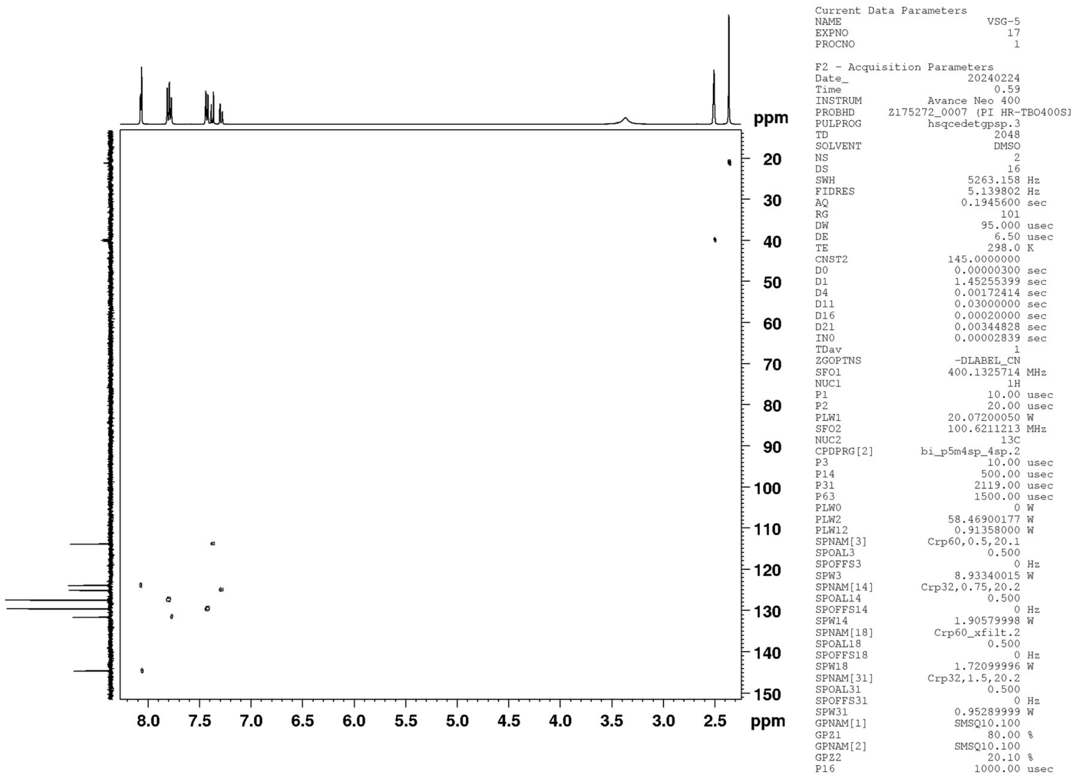

Figure 33S. 2D HSQC NMR spectrum of *N'*-[(*E*)-(5-bromo-1*H*-indol-3-yl)methylidene]-4-methylbenzene-1-sulfonohydrazide, **1e** in DMSO-d_6

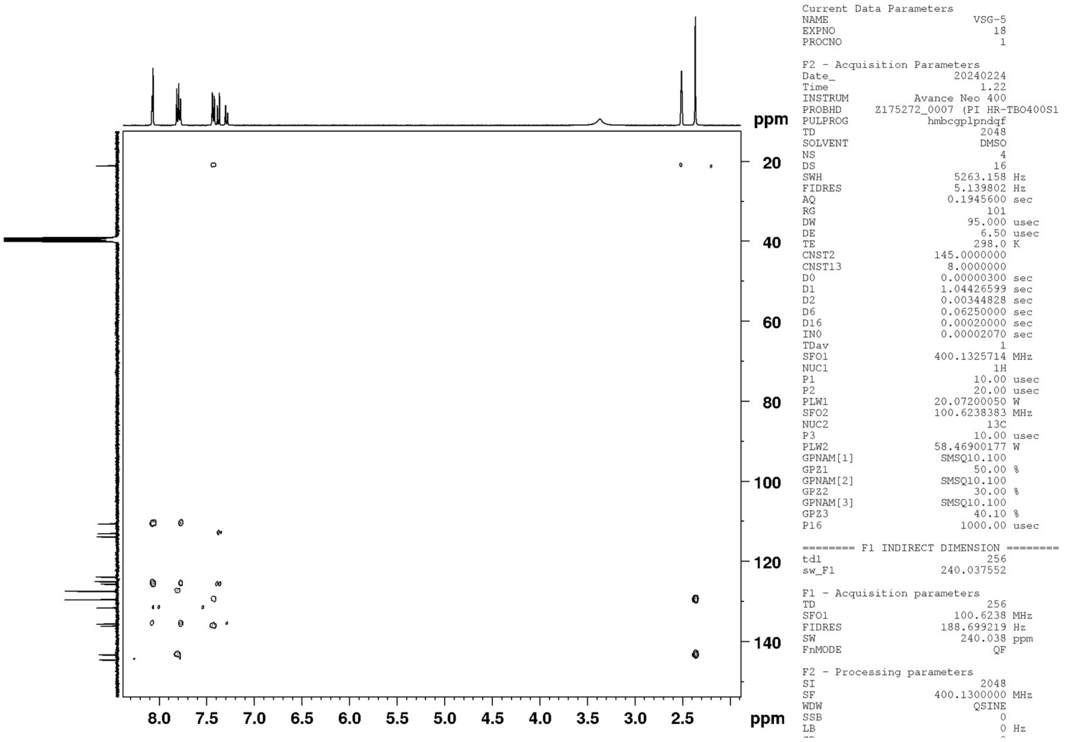

Figure 34S. 2D HMBC NMR spectrum of *N'*-[(*E*)-(5-bromo-1*H*-indol-3-yl)methylidene]-4-methylbenzene-1-sulfonohydrazide, **1e** in DMSO-d_6

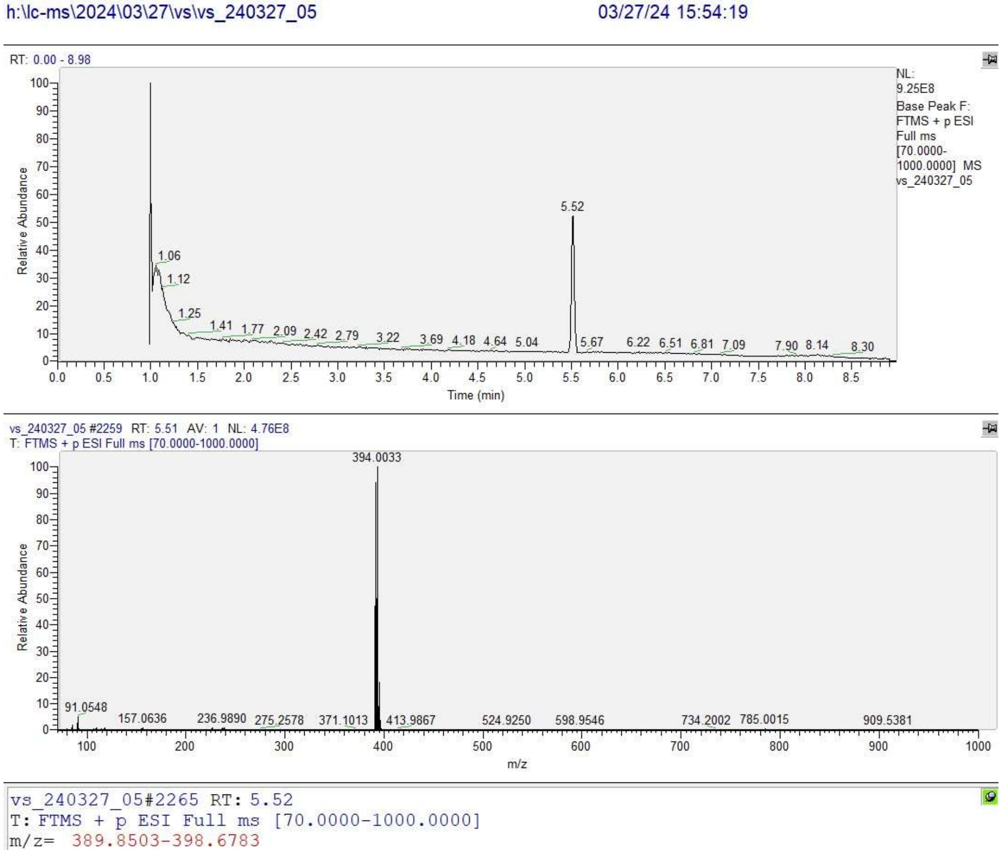

Figure 35S. HRMS of *N*'-[(*E*)-(5-bromo-1*H*-indol-3-yl)methylidene]-4-methylbenzene-1-sulfonohydrazide, **5**

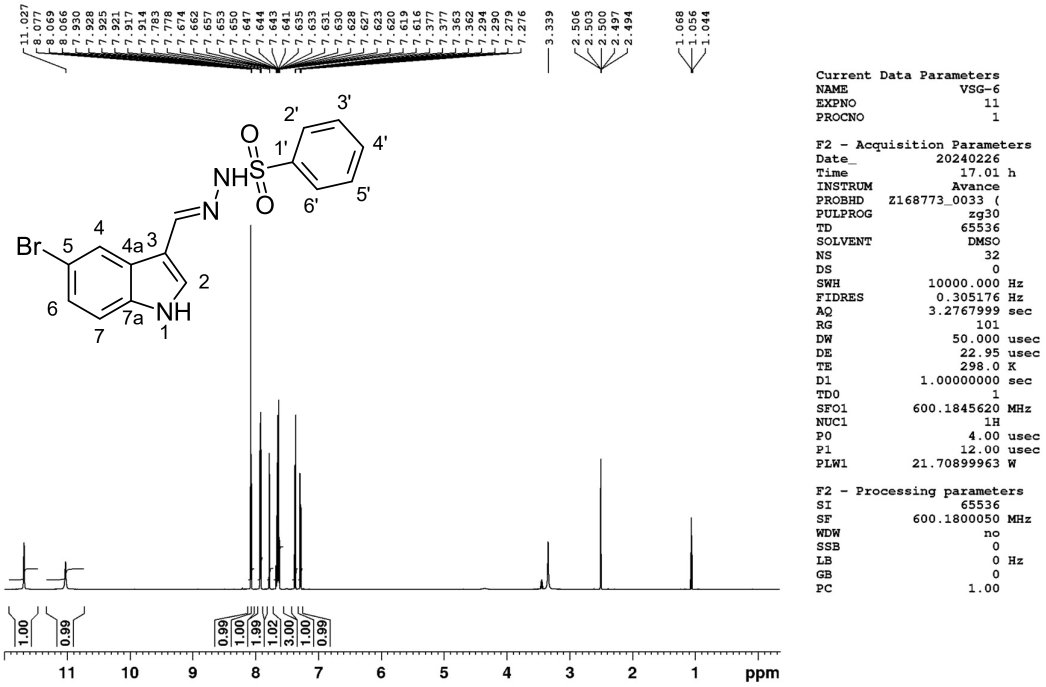

Figure 36S. ^{1}H NMR spectrum of *N*'-[(*E*)-(5-bromo-1H-indol-3-yl)methylidene]-4-methylbenzene-1-sulfonohydrazide, **6** in DMSO-d_6

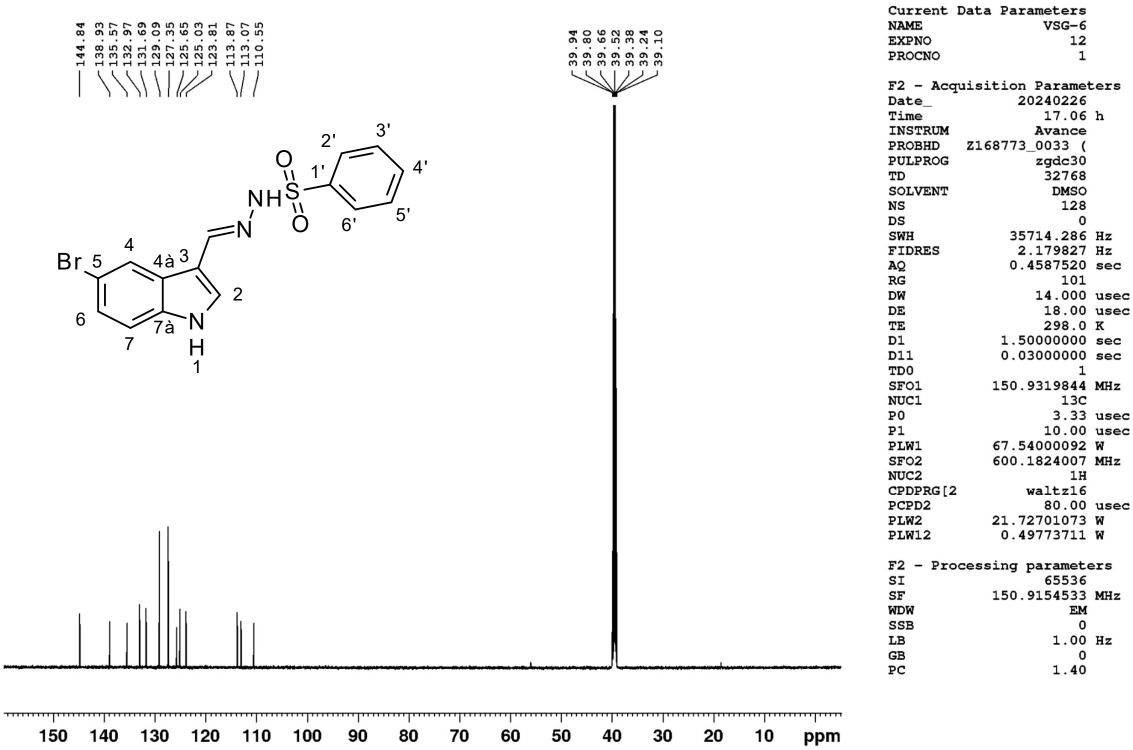

Figure 37S. ^{13}C NMR spectrum of *N'*-[(*E*)-(5-bromo-1H-indol-3-yl)methylidene]-4-methylbenzene-1-sulfonohydrazide, **6** in DMSO-d_6

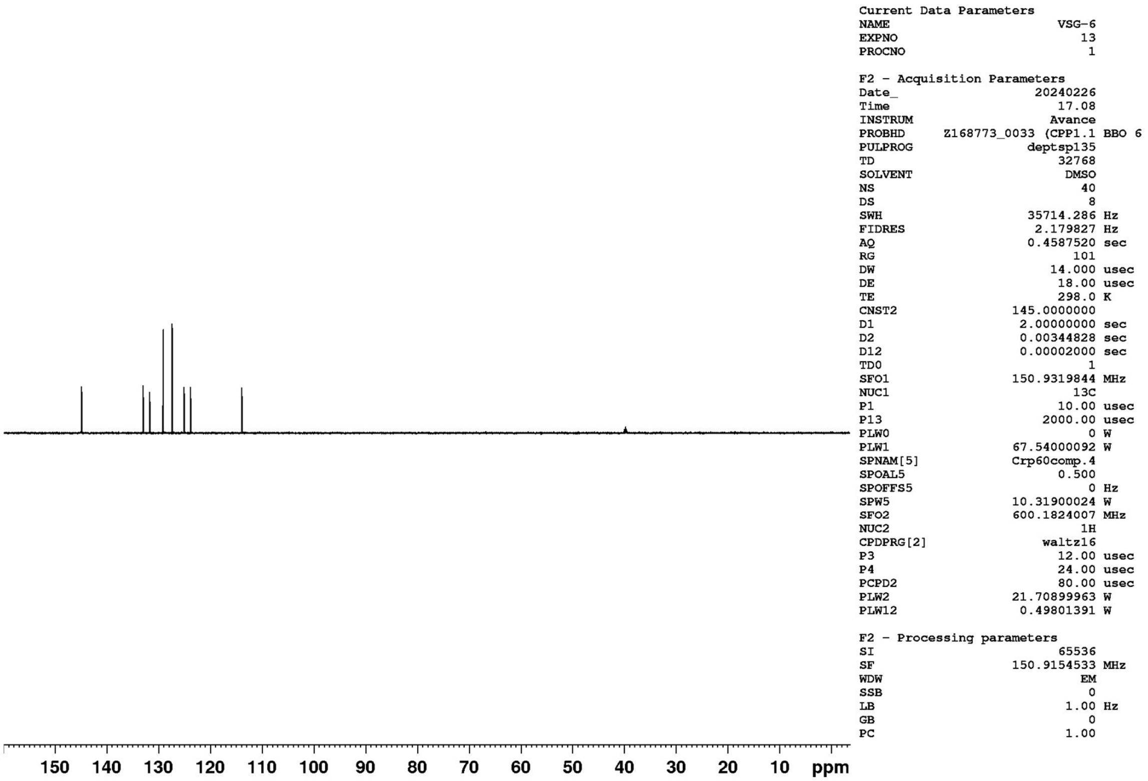

Figure 38S. DEPT-135 NMR spectrum of *N'*-[(*E*)-(5-bromo-1H-indol-3-yl)methylidene]-4-methylbenzene-1-sulfonohydrazide, **6** in DMSO-d_6

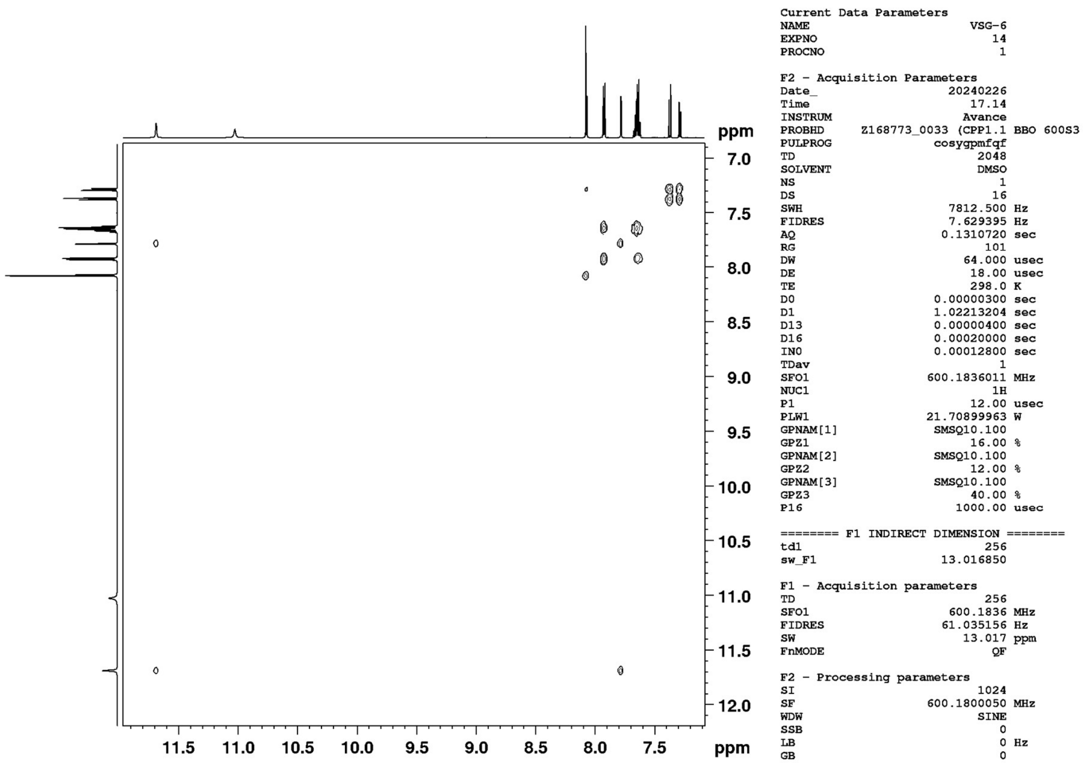

Figure 37S. 2D COSY NMR spectrum of *N*'-[(*E*)-(5-bromo-1H-indol-3-yl)methylidene]-4-methylbenzene-1-sulfonohydrazide, **6** in DMSO-d_6

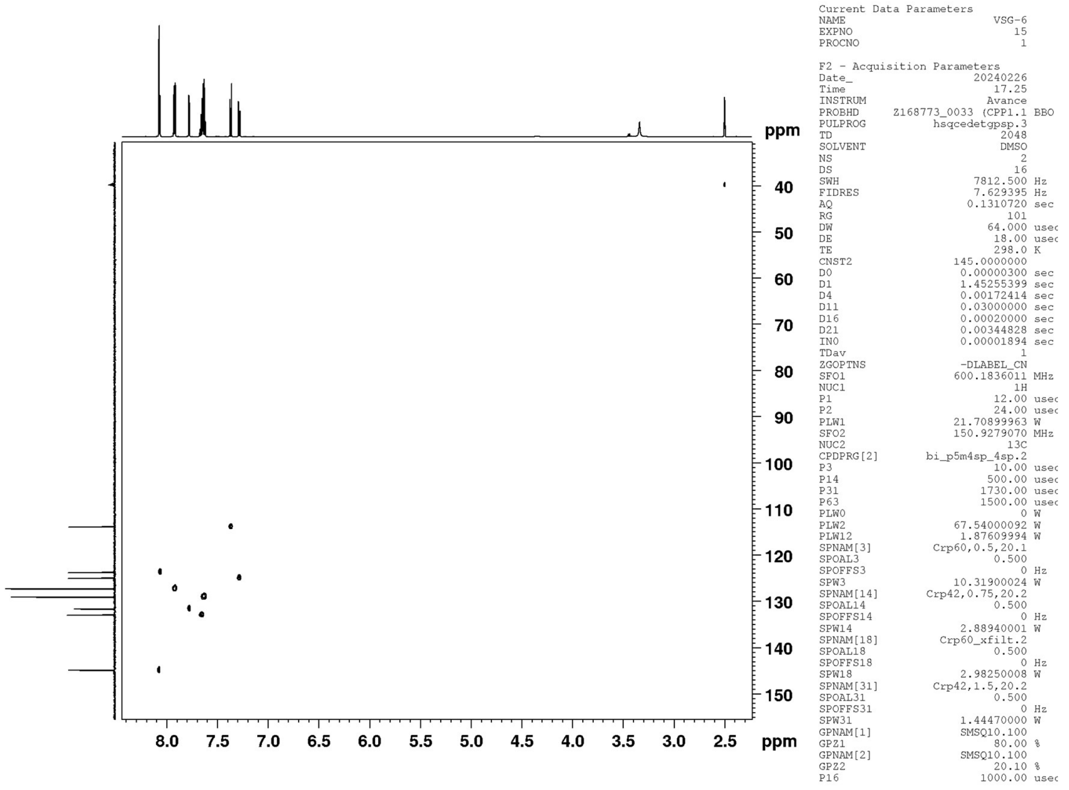

Figure 38S. 2D HSQC NMR spectrum of *N*'-[(*E*)-(5-bromo-1H-indol-3-yl)methylidene]-4-methylbenzene-1-sulfonohydrazide, **6** in DMSO-d_6

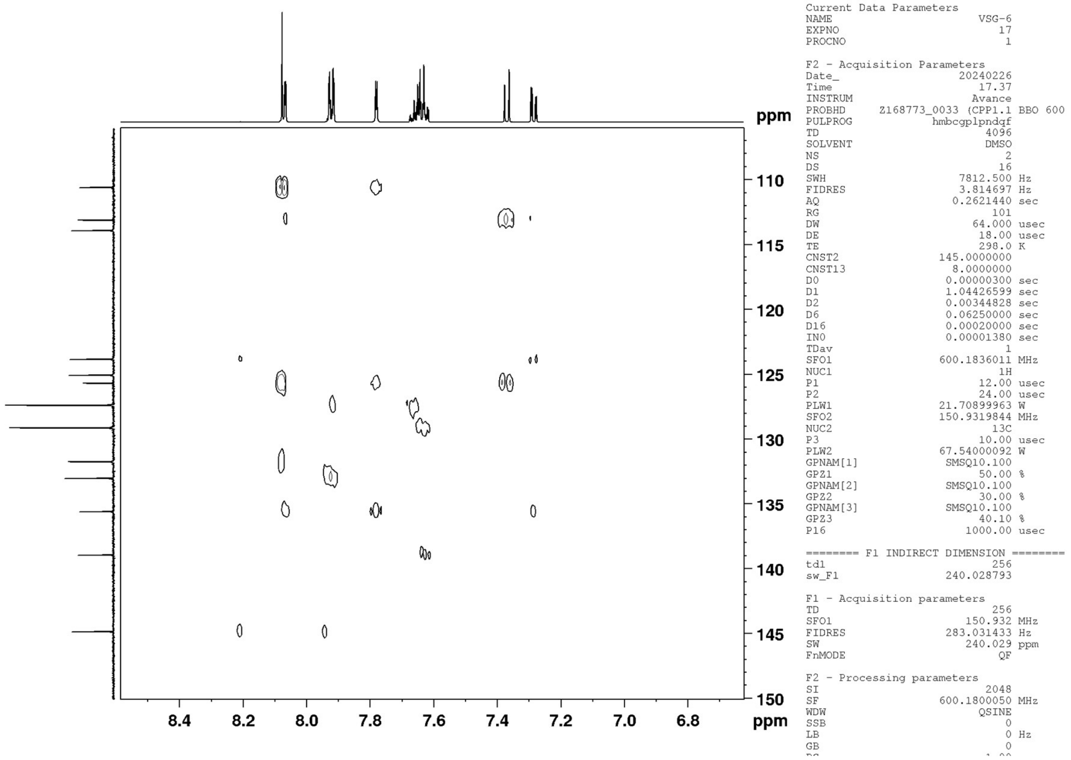

Figure 39S. 2D HMBC NMR spectrum of *N*'-[(*E*)-(5-bromo-1H-indol-3-yl)methylidene]-4-methylbenzene-1-sulfonohydrazide, **6** in DMSO-d_6

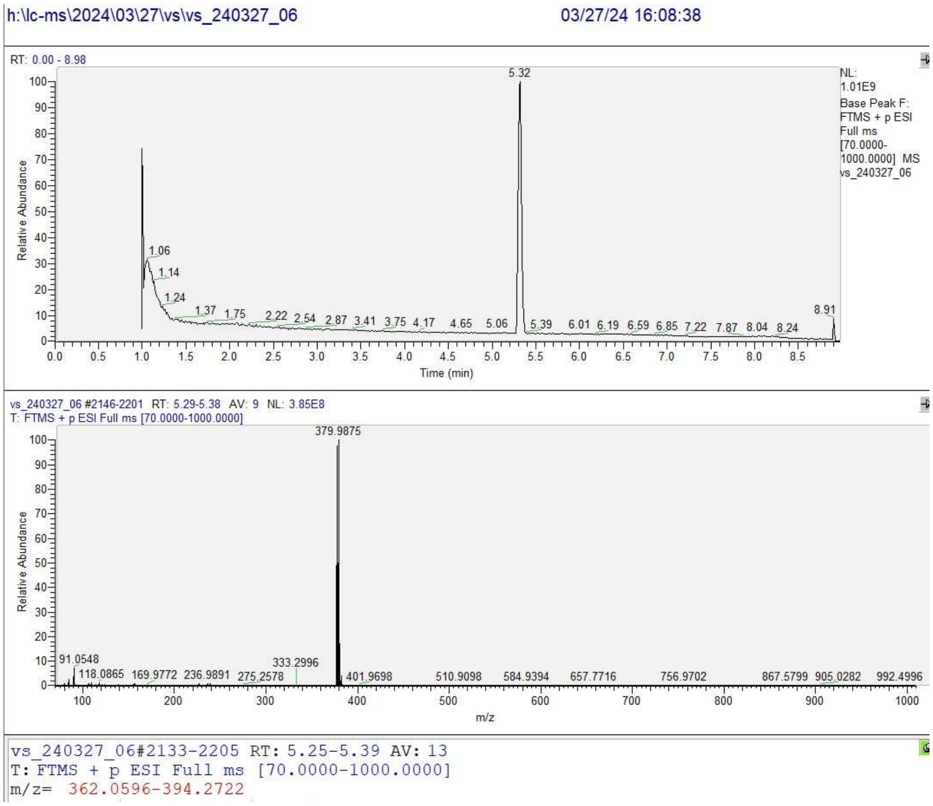

Figure 40S. HRMS of *N*'-[(*E*)-(5-bromo-1H-indol-3-yl)methylidene]-4-methylbenzene-1-sulfonohydrazide, **6**

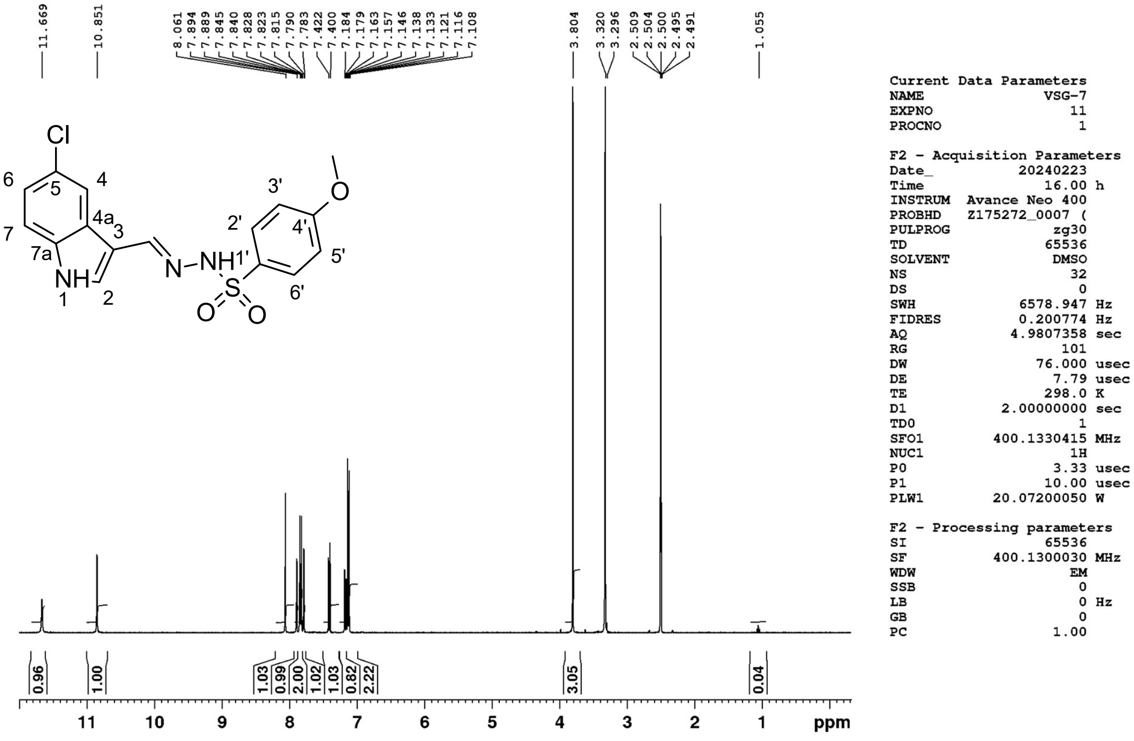

Figure 41S. ^{1}H NMR spectrum of *N*'-[(*E*)-(5-chloro-1*H*-indol-3-yl)methylidene]-4-methoxybenzene-1-sulfonohydrazide, **7** in DMSO-d_6

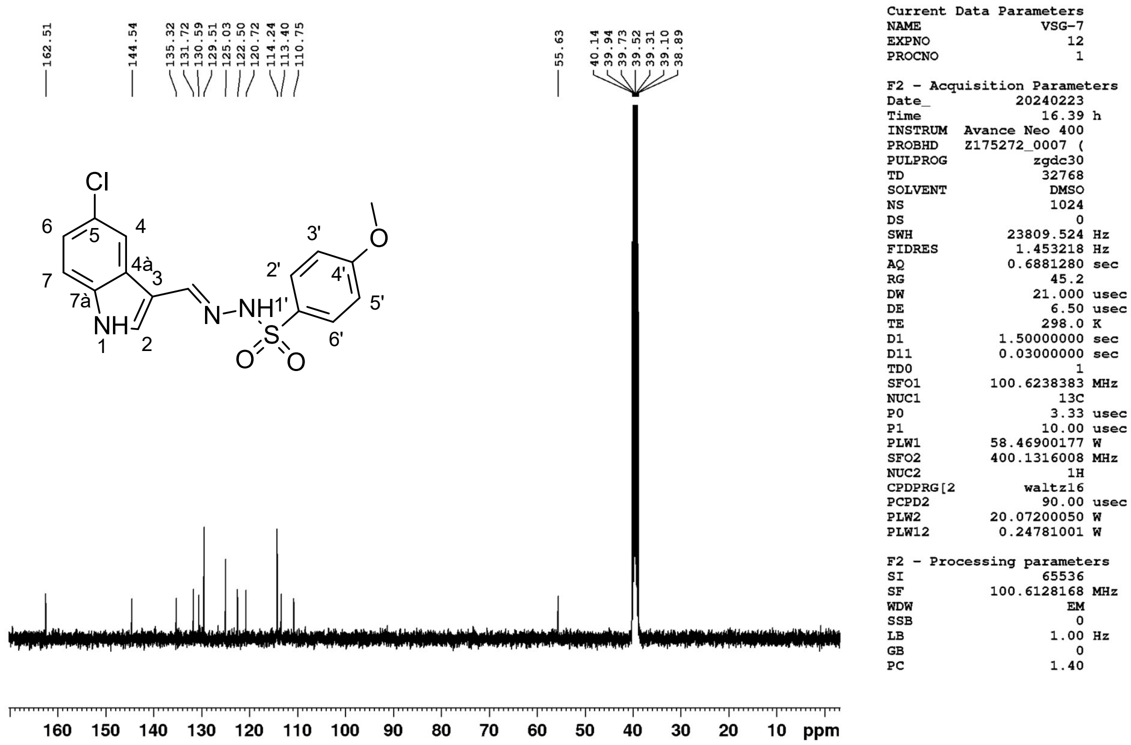

Figure 42S. ^{13}C NMR spectrum of *N*'-[(*E*)-(5-chloro-1*H*-indol-3-yl)methylidene]-4-methoxybenzene-1-sulfonohydrazide, **7** in DMSO-d_6

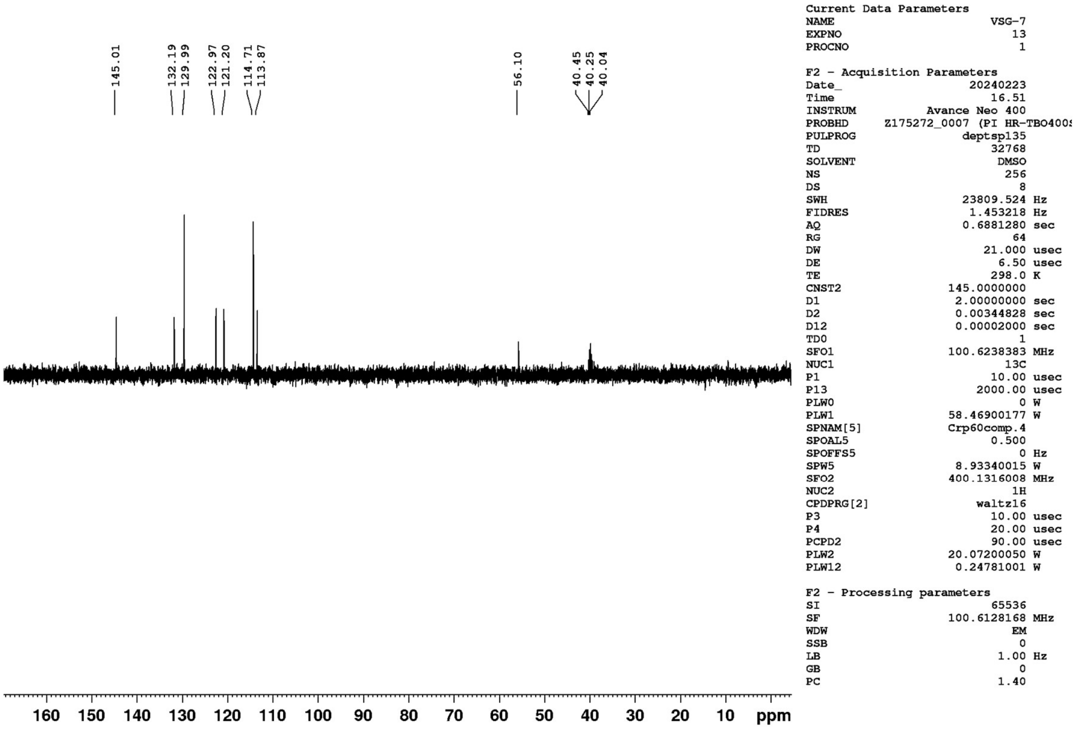

Figure 43S. DEPT-135 NMR spectrum of *N'*-[(*E*)-(5-chloro-1*H*-indol-3-yl)methylidene]-4-methoxybenzene-1-sulfonohydrazide, **7** in DMSO-d_6

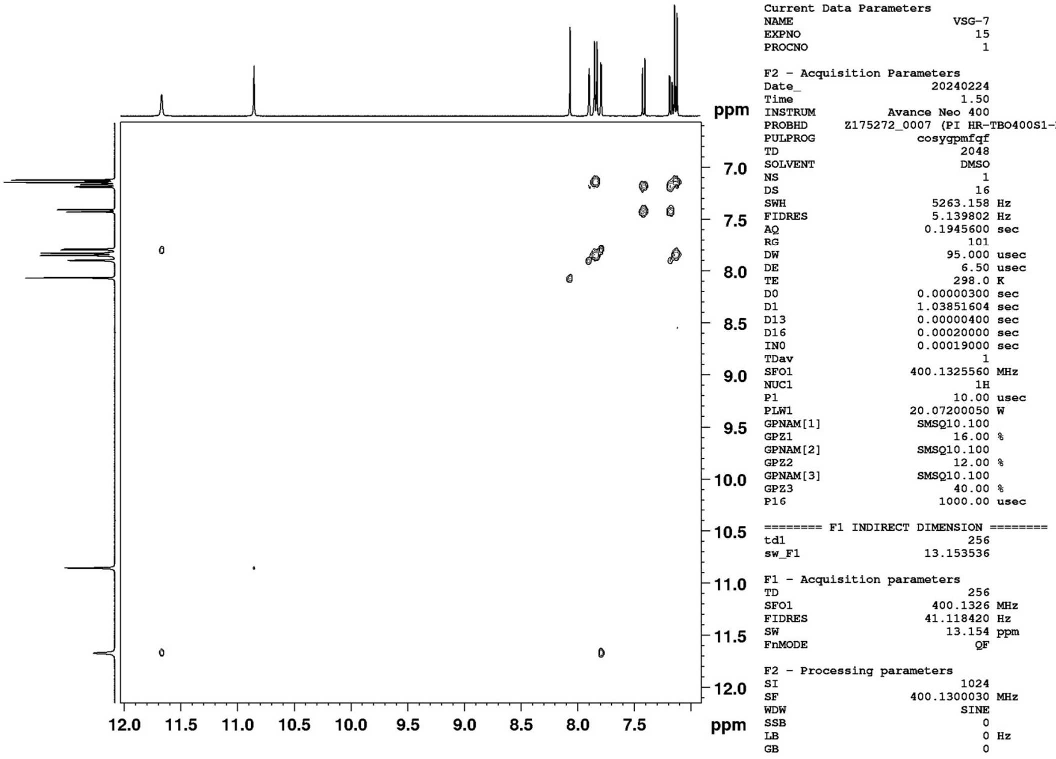

Figure 44S. 2D COSY NMR spectrum of *N'*-[(*E*)-(5-chloro-1*H*-indol-3-yl)methylidene]-4-methoxybenzene-1-sulfonohydrazide, **7** in DMSO-d_6

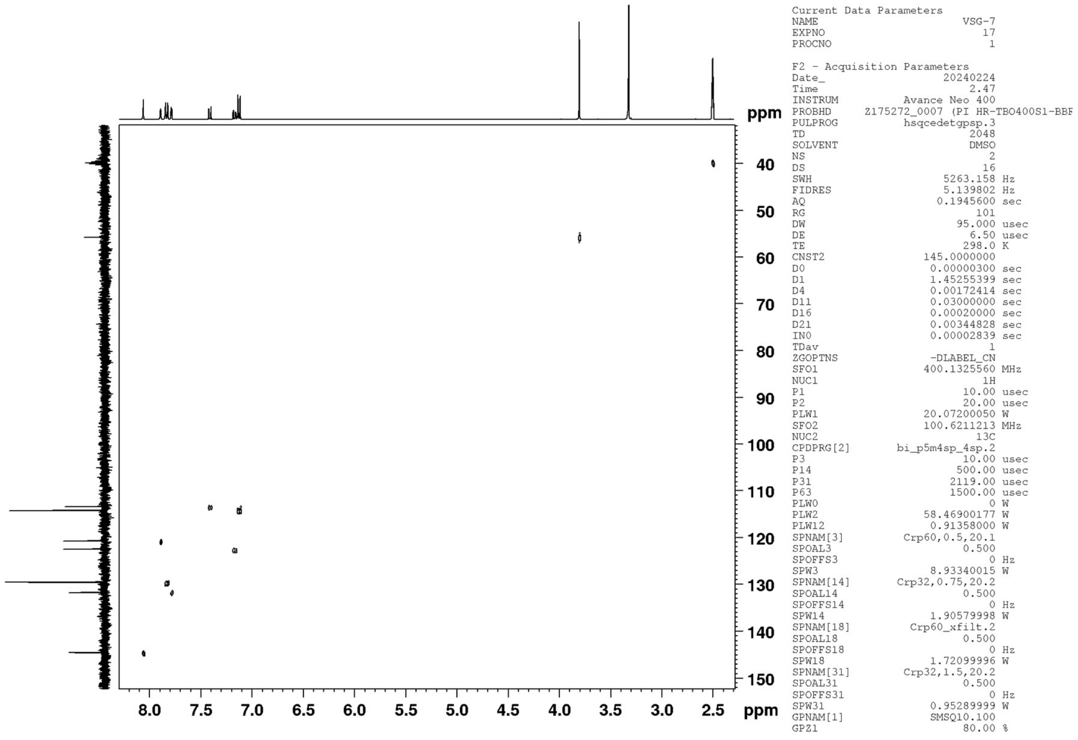

Figure 45S. 2D HSQC NMR spectrum of *N*'-[(*E*)-(5-chloro-1*H*-indol-3-yl)methylidene]-4-methoxybenzene-1-sulfonohydrazide, **7** in DMSO-d_6

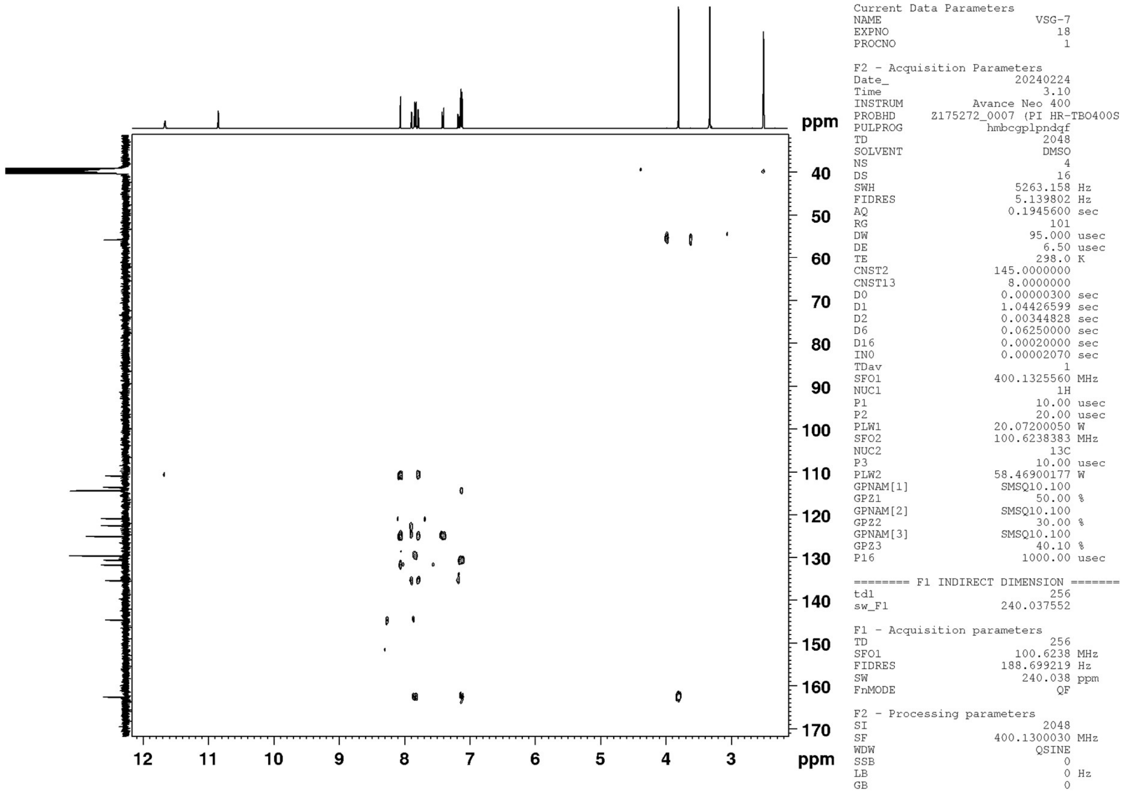

Figure 46S. 2D HMBC NMR spectrum of *N*'-[(*E*)-(5-chloro-1*H*-indol-3-yl)methylidene]-4-methoxybenzene-1-sulfonohydrazide, **7** in DMSO-d_6

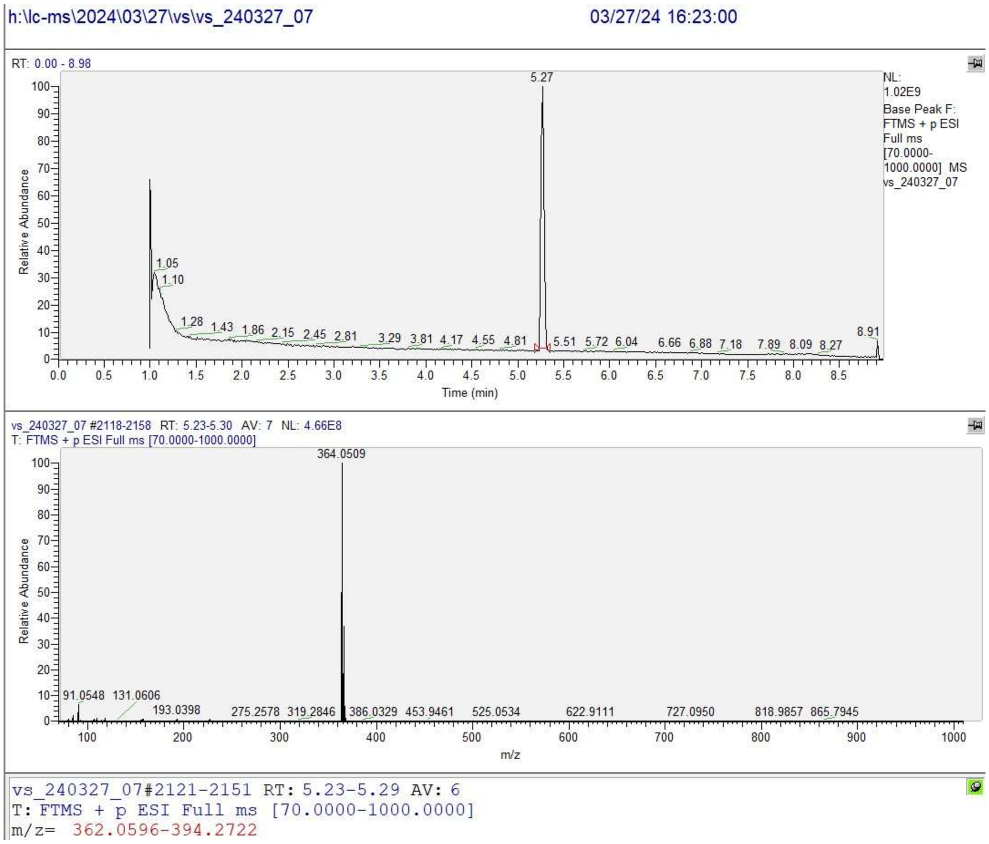

Figure 47S. HRMS of *N'*-[(*E*)-(5-chloro-1*H*-indol-3-yl)methylidene]-4-methoxybenzene-1-sulfonohydrazide, **7**

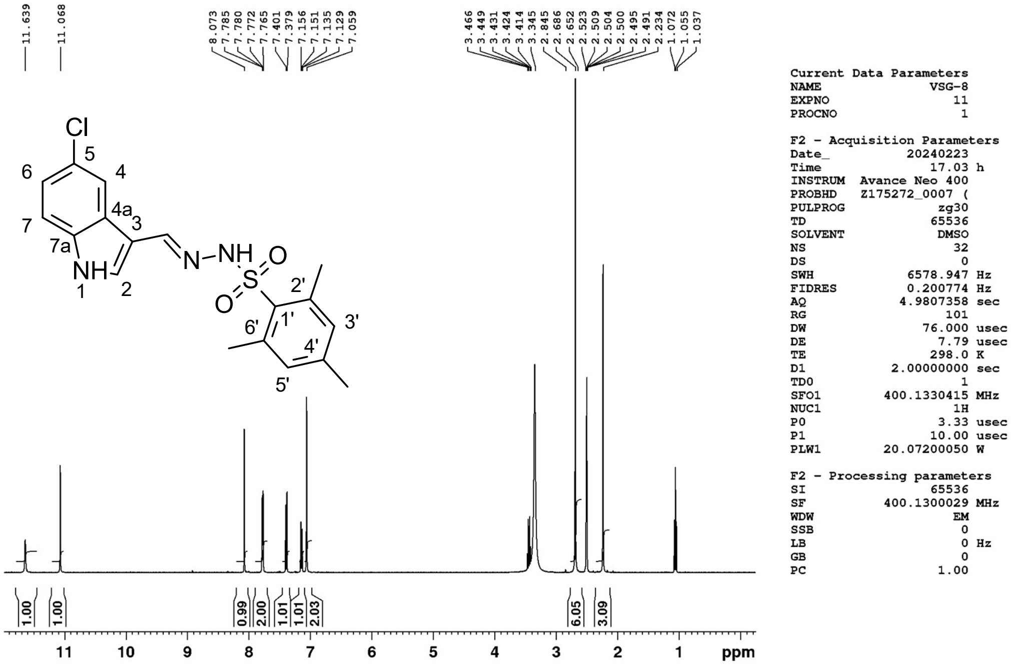

Figure 48S. ^{1}H NMR spectrum of *N'*-[(*E*)-(5-chloro-1*H*-indol-3-yl)methylidene]-2,4,6-trimethylbenzene-1-sulfonohydrazide, **8** in DMSO-d_6

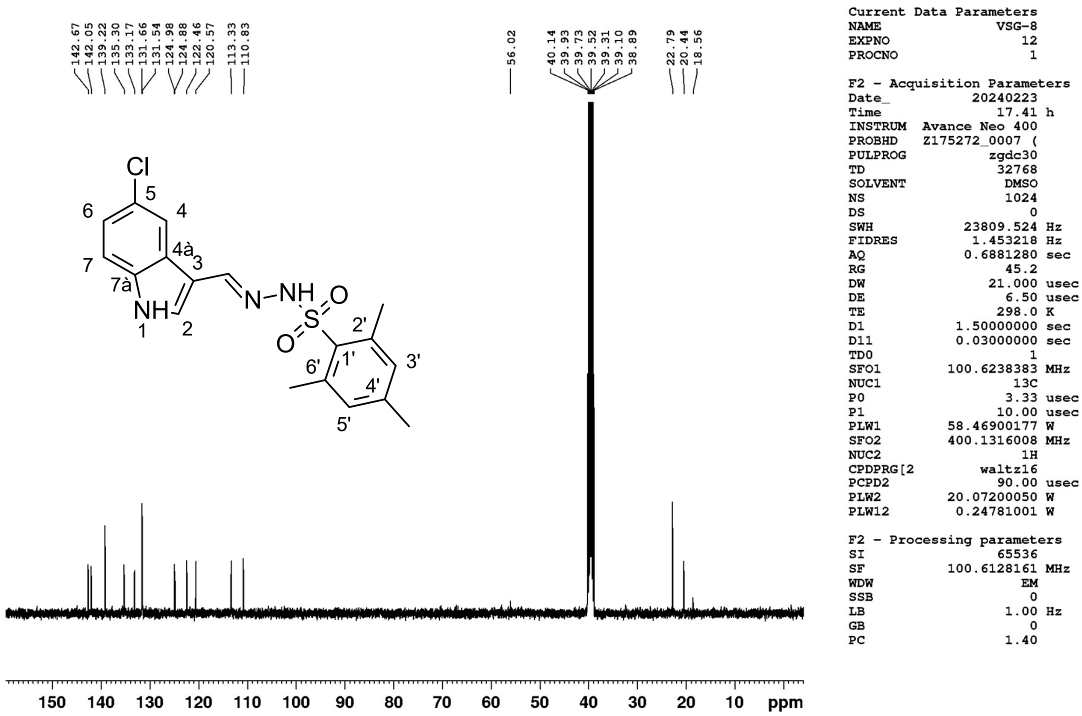

Figure 49S. ^{13}C NMR spectrum of *N'*-[(*E*)-(5-chloro-1*H*-indol-3-yl)methylidene]-2,4,6-trimethylbenzene-1-sulfonohydrazide, **8** in DMSO-d_6

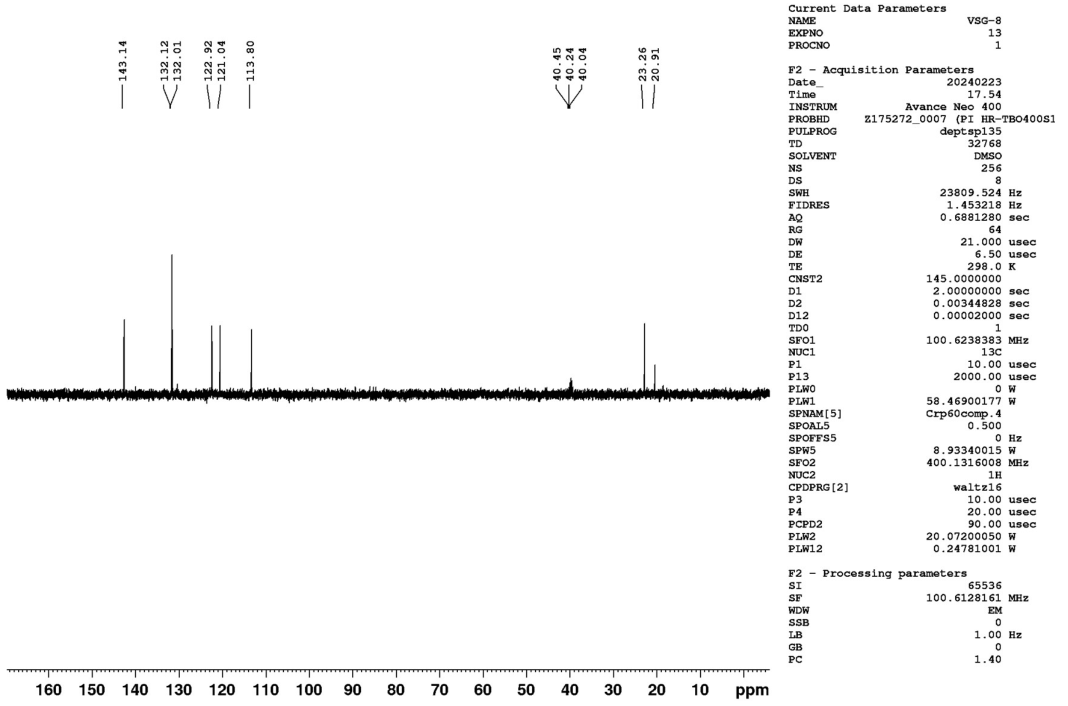

Figure 50S. DEPT-135 NMR spectrum of *N'*-[(*E*)-(5-chloro-1*H*-indol-3-yl)methylidene]-2,4,6-trimethylbenzene-1-sulfonohydrazide, **8** in DMSO-d_6

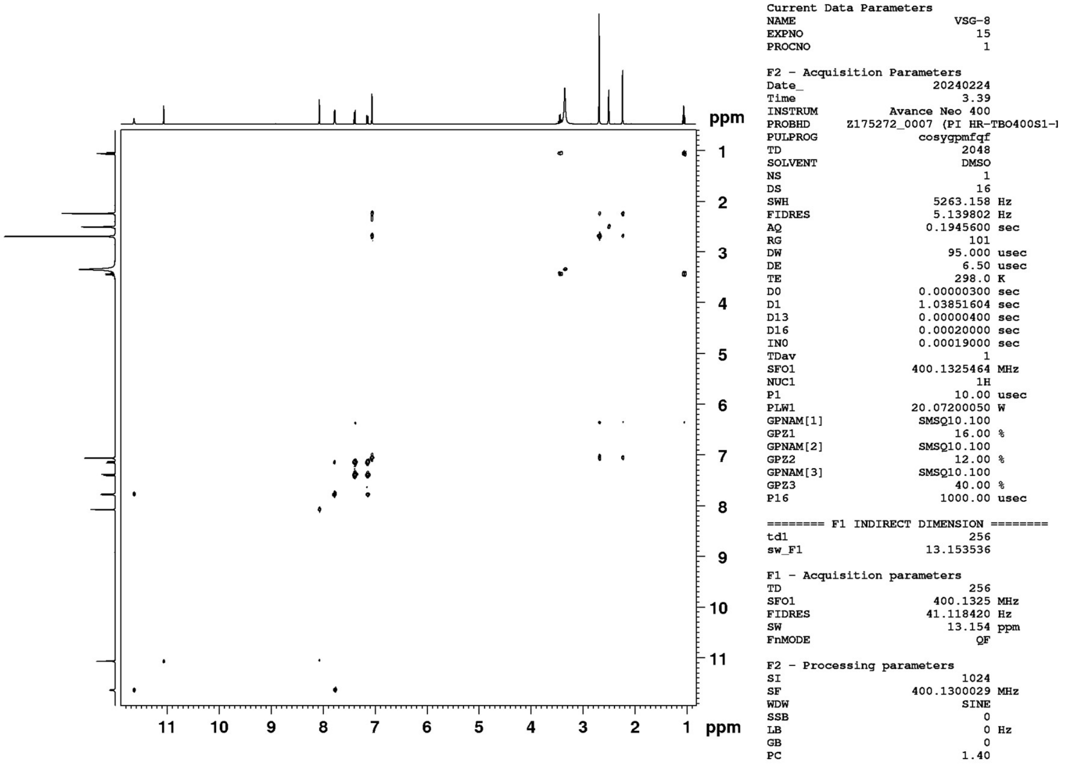

Figure 51S. 2D COSY NMR spectrum of *N'*-[(*E*)-(5-chloro-1*H*-indol-3-yl)methylidene]-2,4,6-trimethylbenzene-1-sulfonohydrazide, **8** in DMSO-d_6

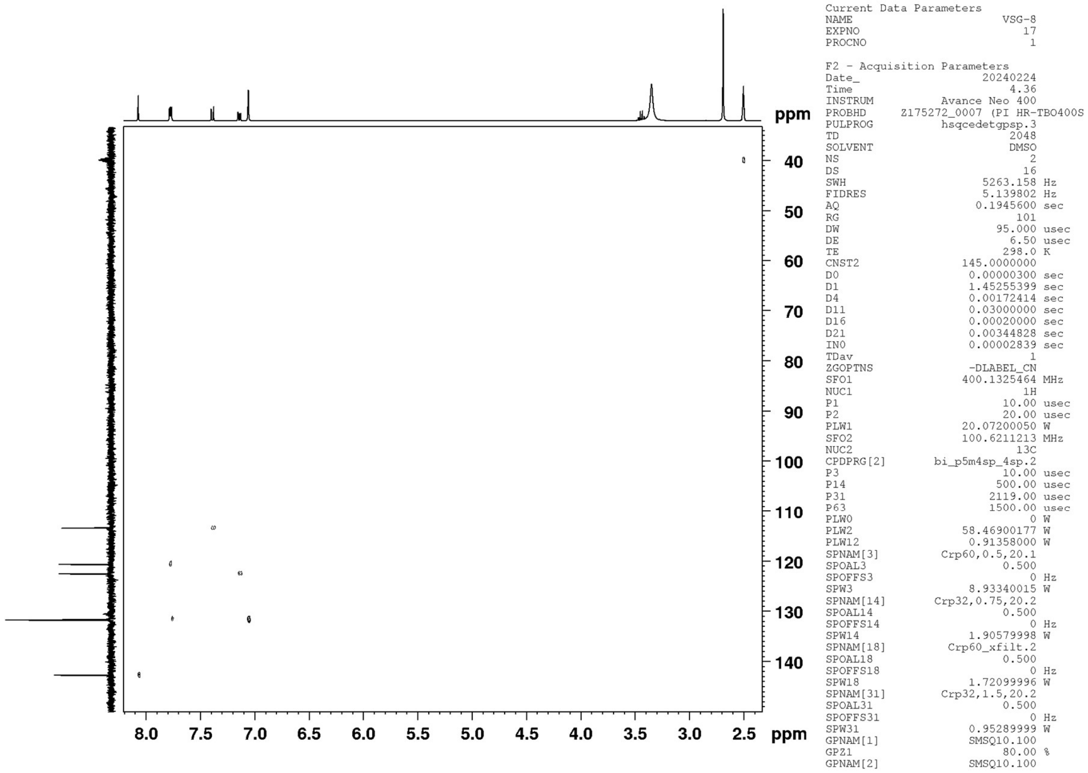

Figure 52S. 2D HSQC NMR spectrum of *N*'-[(*E*)-(5-chloro-1*H*-indol-3-yl)methylidene]-2,4,6-trimethylbenzene-1-sulfonohydrazide, **8** in DMSO-d_6

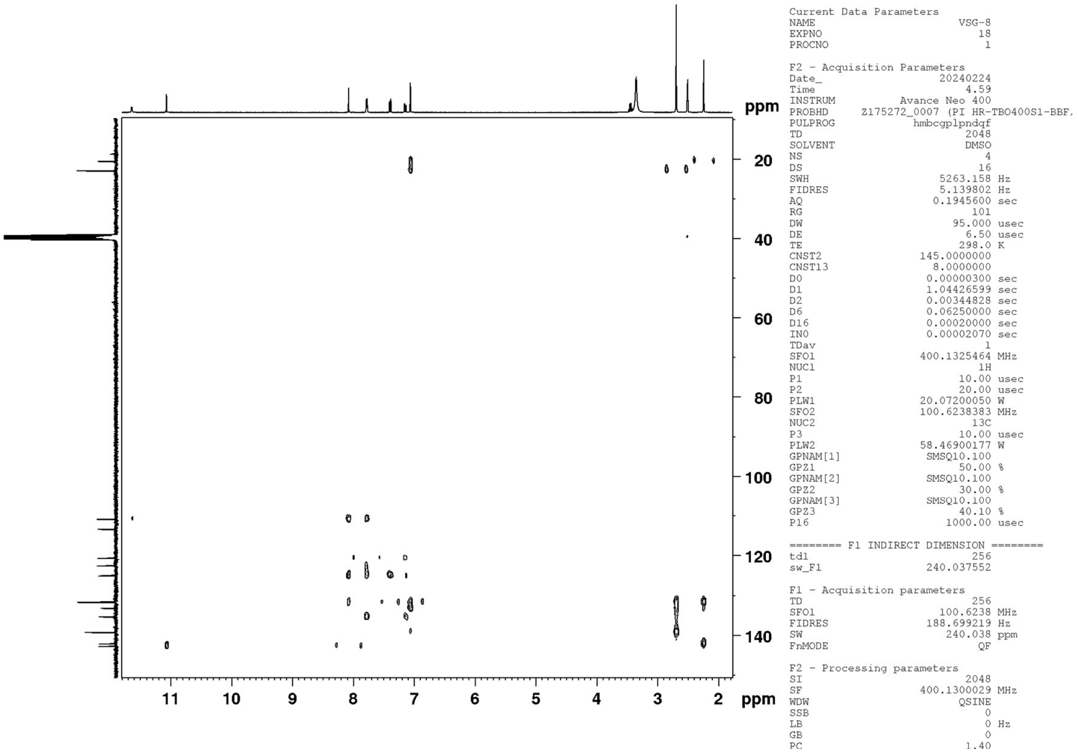

Figure 53S. 2D HMBC NMR spectrum of *N*'-[(*E*)-(5-chloro-1*H*-indol-3-yl)methylidene]-2,4,6-trimethylbenzene-1-sulfonohydrazide, **8** in DMSO-d_6

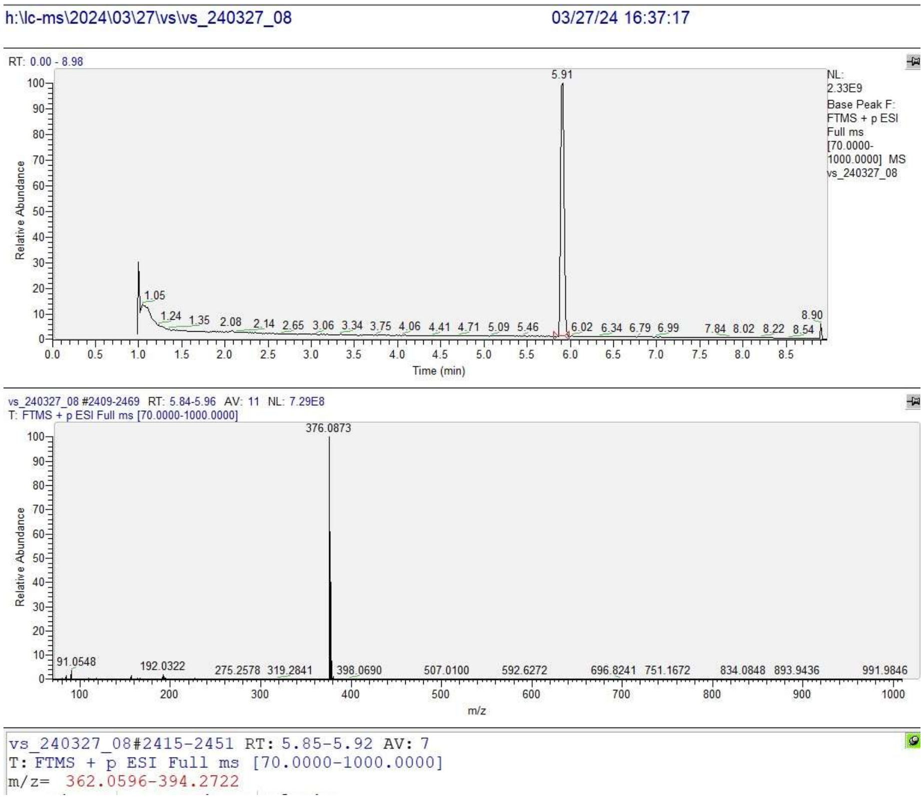

Figure 54S. HRMS of *N'*-[(*E*)-(5-chloro-1*H*-indol-3-yl)methylidene]-2,4,6-trimethylbenzene-1-sulfonohydrazide, **8**

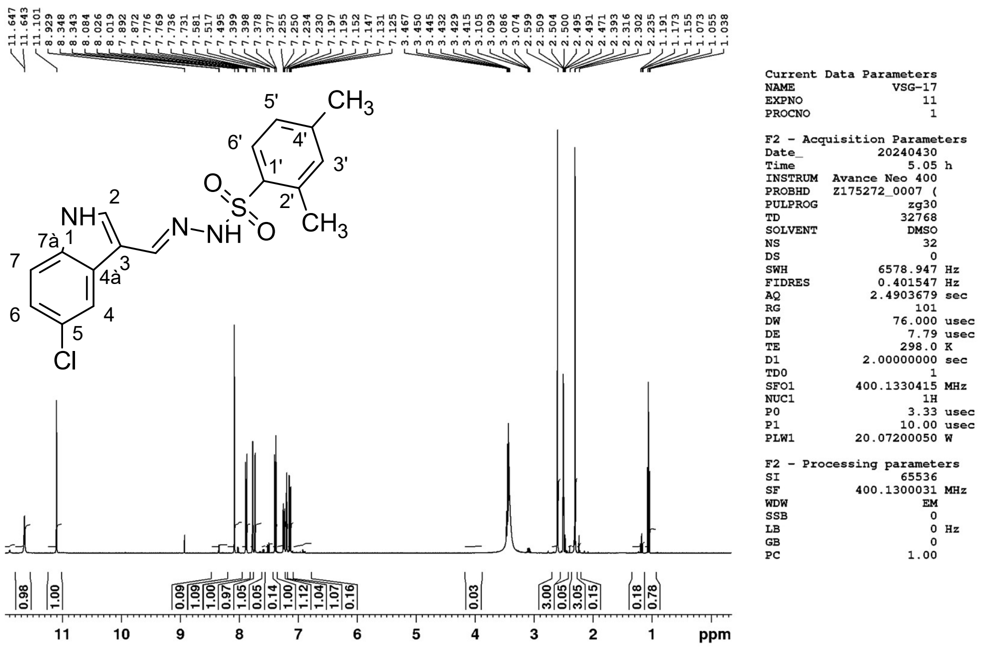

Figure 55S. ^{1}H NMR spectrum of *N'*-[(*E*)-(5-chloro-1*H*-indol-3-yl)methylidene]-2,4-dimethylbenzene-1-sulfonohydrazide, **9** in DMSO-d_6

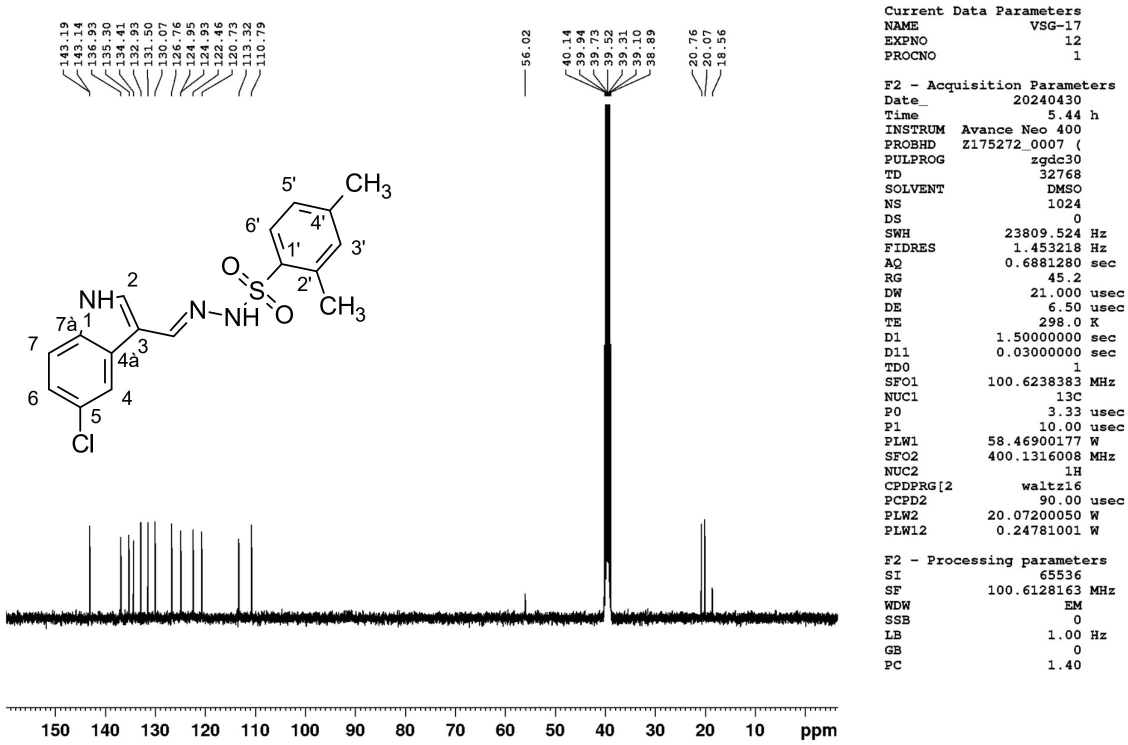

Figure 56S. ^{13}C NMR spectrum of *N'*-[(*E*)-(5-chloro-1*H*-indol-3-yl)methylidene]-2,4-dimethylbenzene-1-sulfonohydrazide, **9** in DMSO-d_6

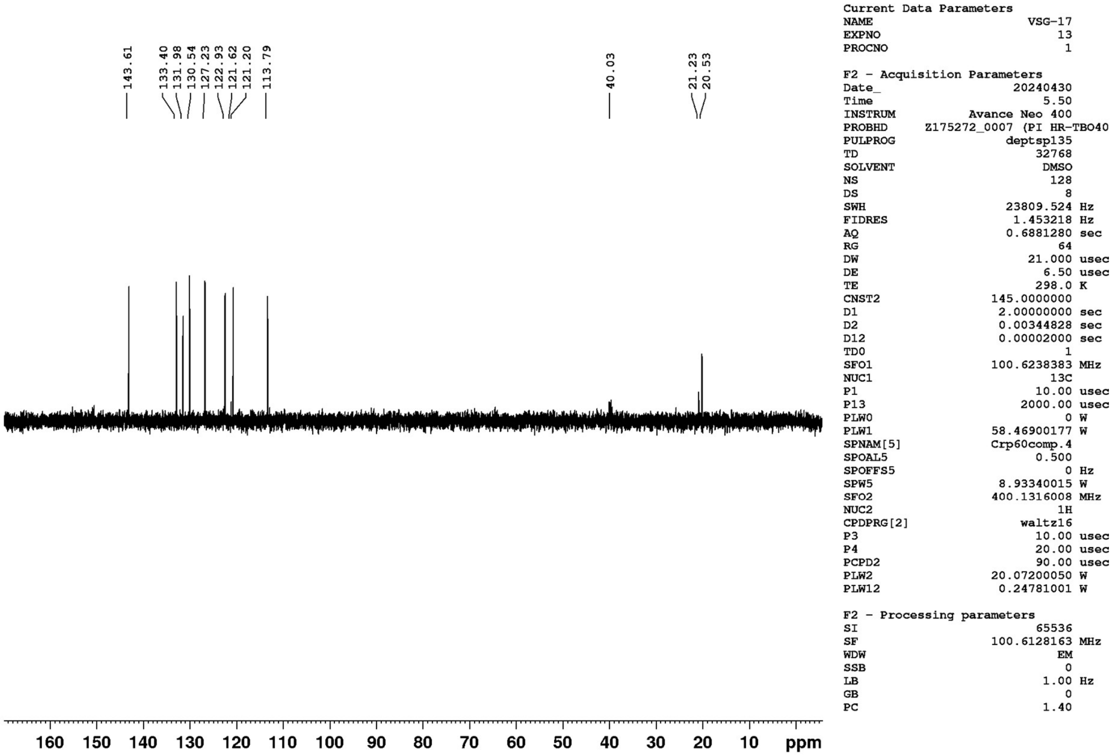

Figure 57S. DEPT-135 NMR spectrum of *N'*-[(*E*)-(5-chloro-1*H*-indol-3-yl)methylidene]-2,4-dimethylbenzene-1-sulfonohydrazide, **9** in DMSO-d_6

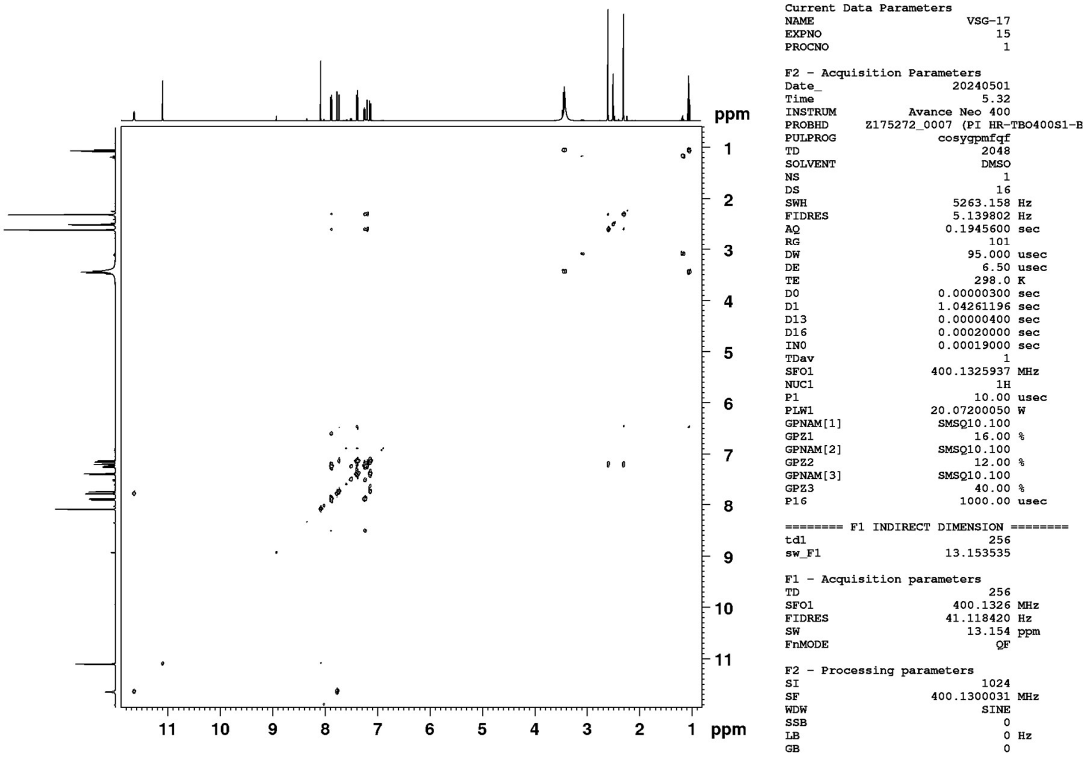

Figure 58S. 2D COSY NMR spectrum of *N'*-[(*E*)-(5-chloro-1*H*-indol-3-yl)methylidene]-2,4-dimethylbenzene-1-sulfonohydrazide, **9** in DMSO-d_6

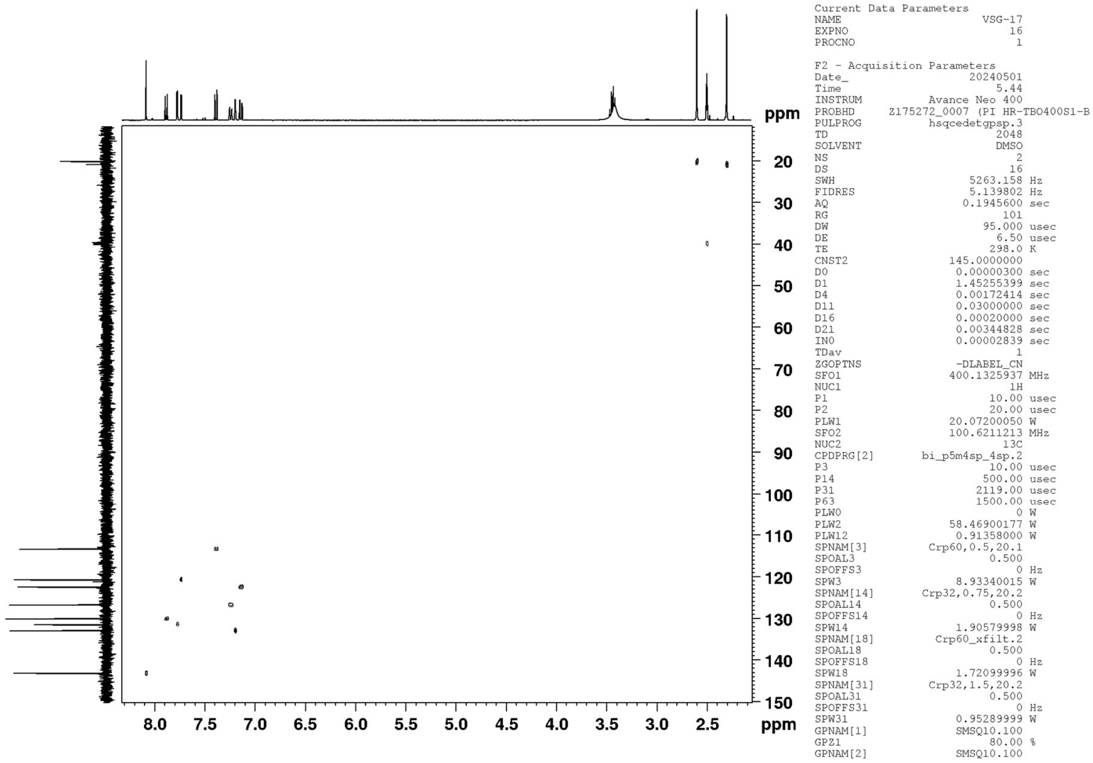

Figure 59S. 2D HSQC NMR spectrum of *N*'-[(*E*)-(5-chloro-1*H*-indol-3-yl)methylidene]-2,4-dimethylbenzene-1-sulfonohydrazide, **9** in DMSO-d_6

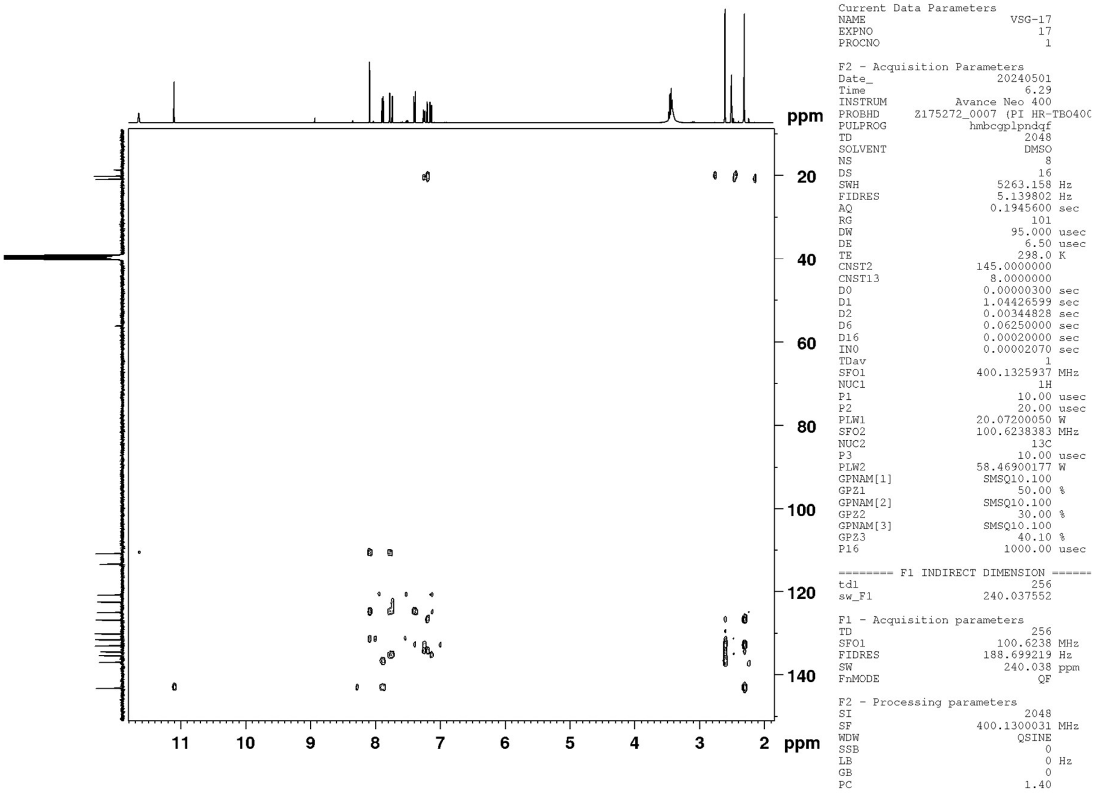

Figure 60S. 2D HMBC NMR spectrum of *N'*-[(*E*)-(5-chloro-1*H*-indol-3-yl)methylidene]-2,4-dimethylbenzene-1-sulfonohydrazide, **9** in DMSO-d_6

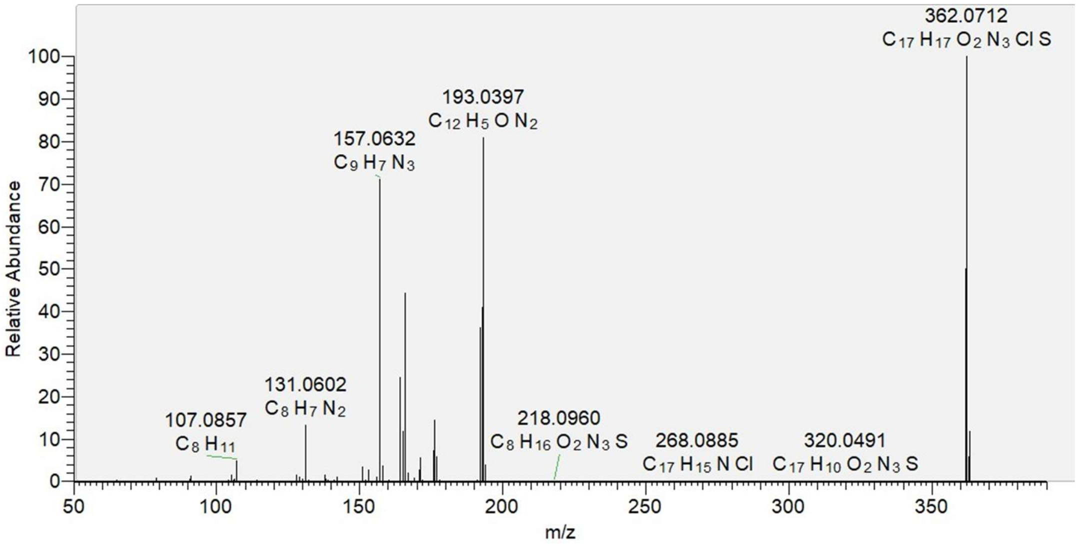

Figure 61S. HRMS of *N'*-[(*E*)-(5-chloro-1*H*-indol-3-yl)methylidene]-2,4-dimethylbenzene-1-sulfonohydrazide, **9**

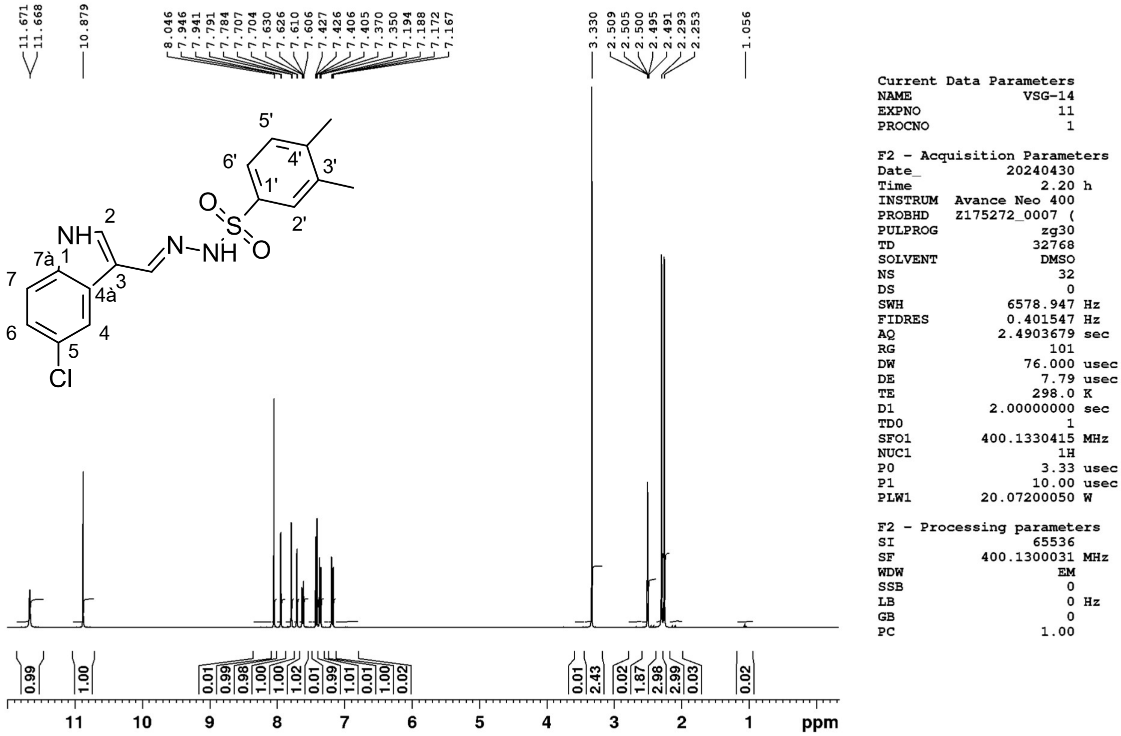

Figure 62S. ^{1}H NMR spectrum of *N'*-[(*E*)-(5-chloro-1*H*-indol-3-yl)methylidene]-3,4-dimethylbenzene-1-sulfonohydrazide, **10** in DMSO-d_6

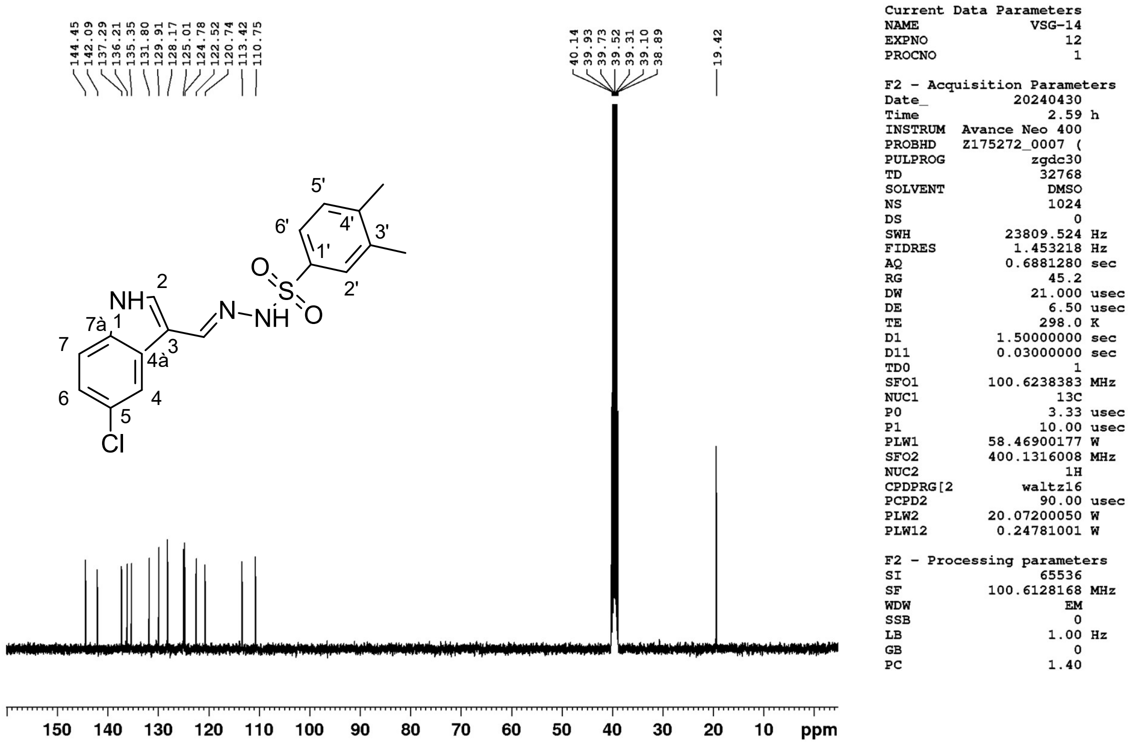

Figure 63S. ^{13}C NMR spectrum of *N'*-[(*E*)-(5-chloro-1*H*-indol-3-yl)methylidene]-3,4-dimethylbenzene-1-sulfonohydrazide, **10** in DMSO-d_6

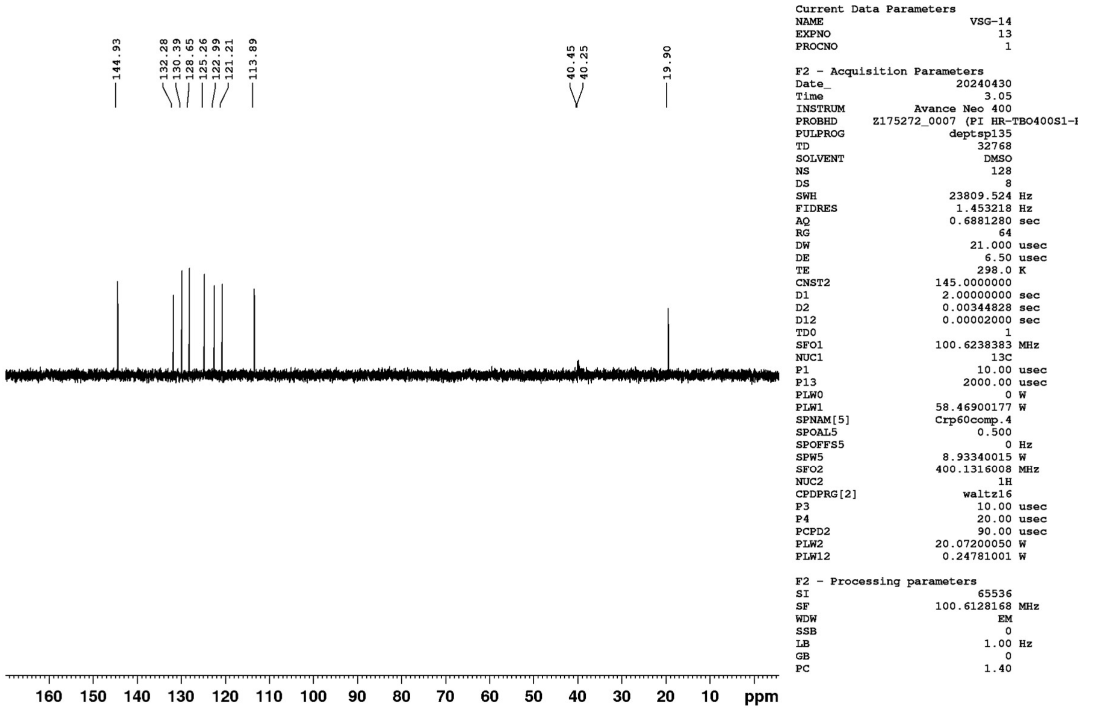

Figure 64S. DEPT-135 NMR spectrum of *N'*-[(*E*)-(5-chloro-1*H*-indol-3-yl)methylidene]-3,4-dimethylbenzene-1-sulfonohydrazide, **10** in DMSO-d_6

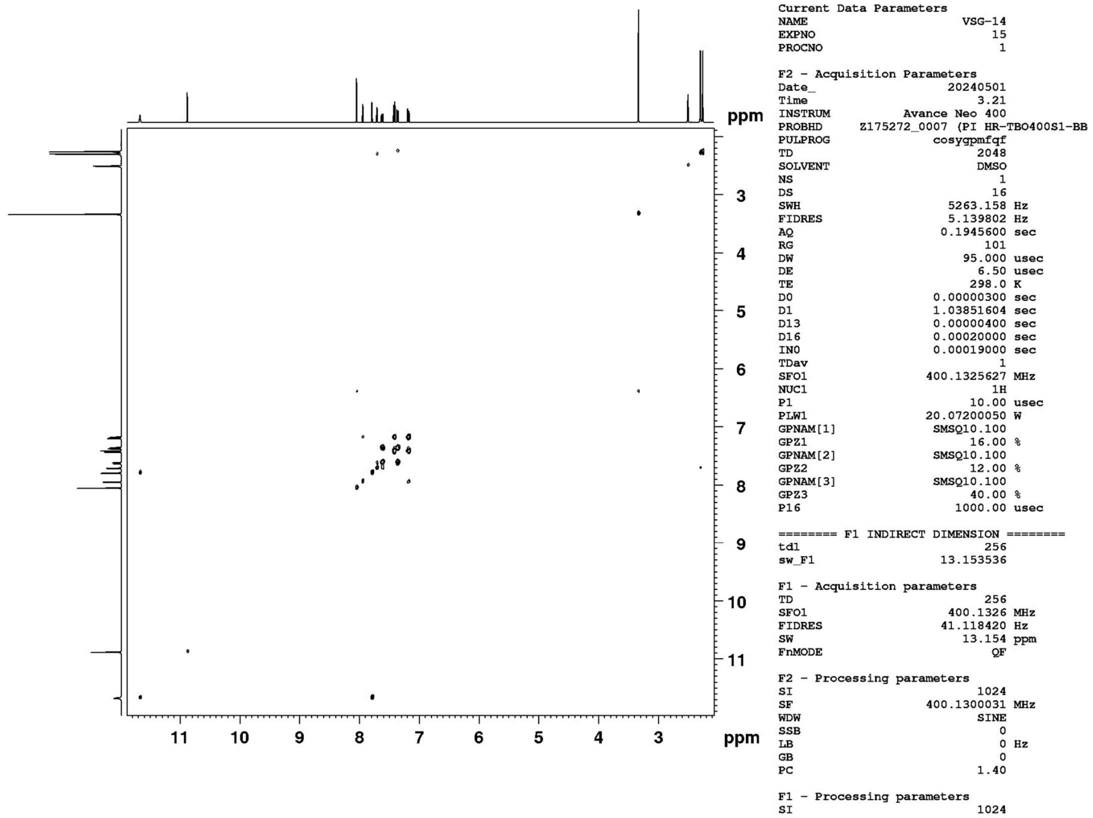

Figure 65S. 2D COSY NMR spectrum of *N'*-[(*E*)-(5-chloro-1*H*-indol-3-yl)methylidene]-3,4-dimethylbenzene-1-sulfonohydrazide, **10** in DMSO-d_6

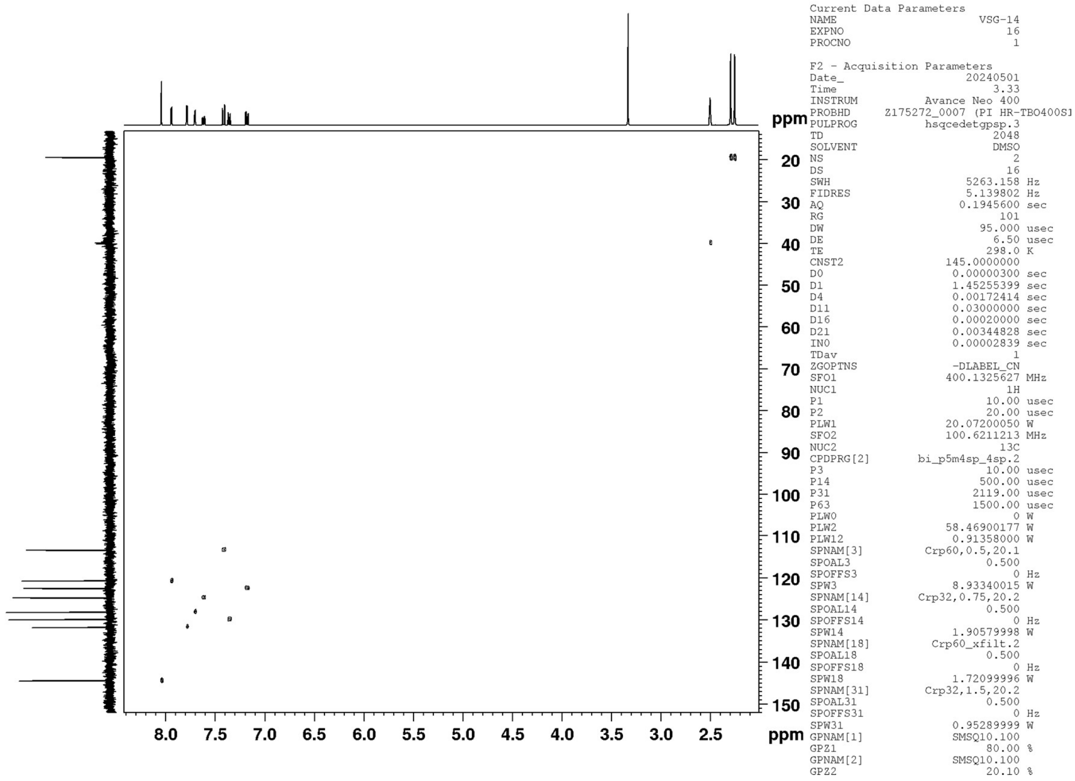

Figure 66S. 2D HSQC NMR spectrum of *N'*-[(*E*)-(5-chloro-1*H*-indol-3-yl)methylidene]-3,4-dimethylbenzene-1-sulfonohydrazide, **10** in DMSO-d_6

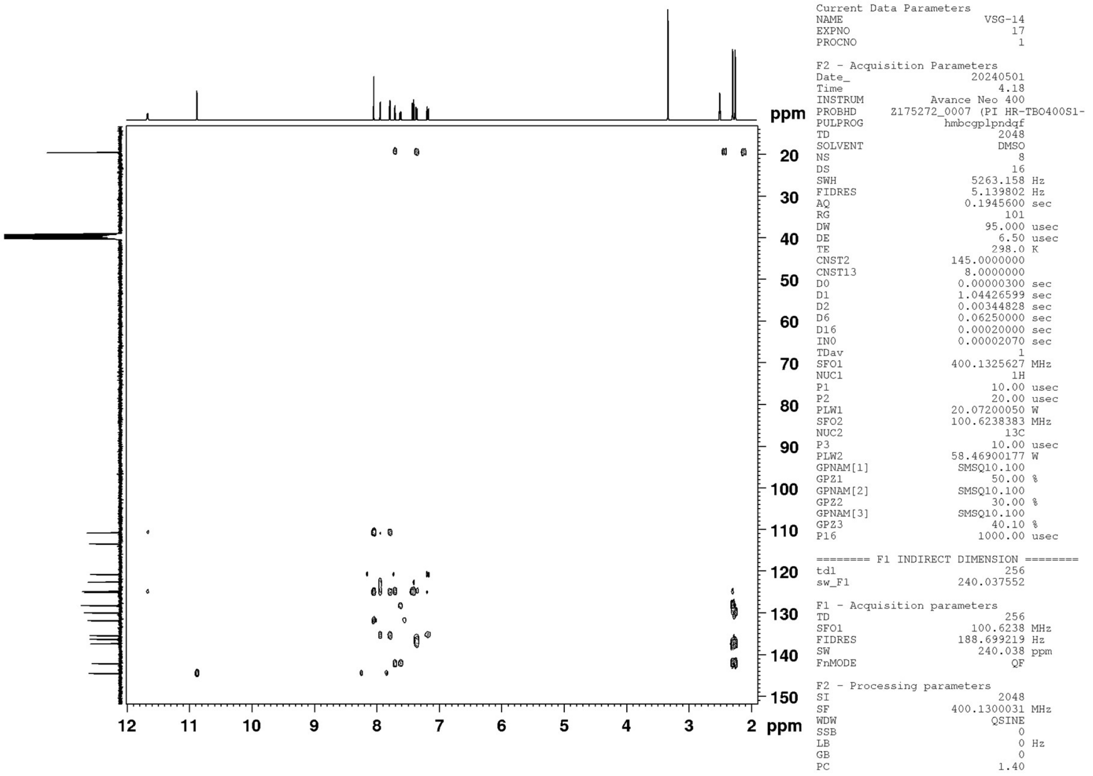

Figure 67S. 2D HMBC NMR spectrum of *N'*-[(*E*)-(5-chloro-1*H*-indol-3-yl)methylidene]-3,4-dimethylbenzene-1-sulfonohydrazide, **10** in DMSO-d_6

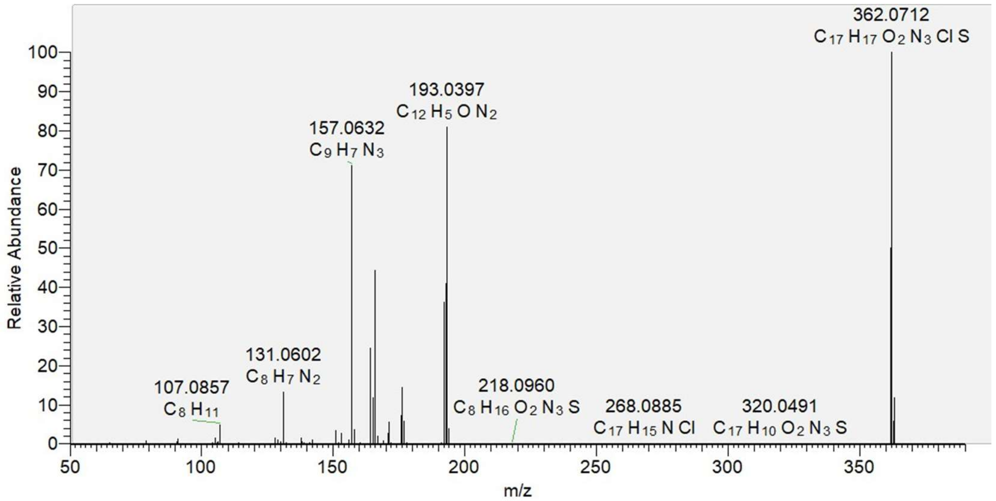

Figure 68S. HRMS of *N'*-[(*E*)-(5-chloro-1*H*-indol-3-yl)methylidene]-3,4-dimethylbenzene-~~1 sulfonohydrazide~~, **10**

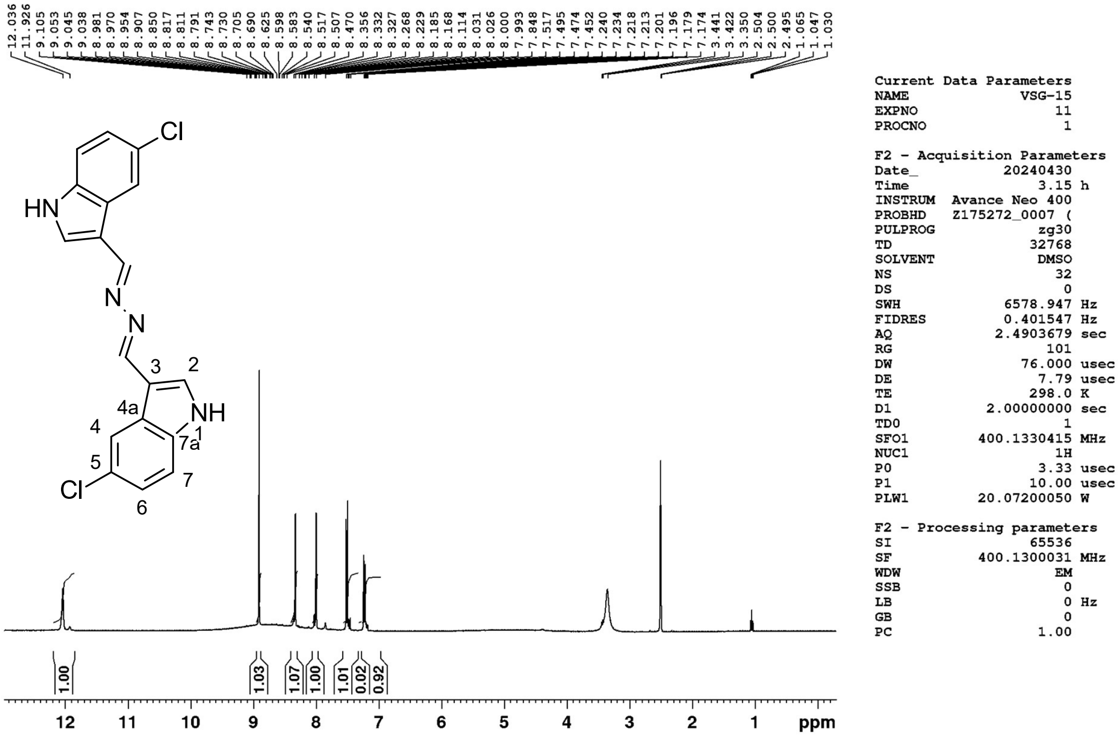

Figure 69S. ^{1}H NMR spectrum of 3,3'-[(1*E*,2*E*)-hydrazinediylidenedi-(*E*)-methanylylidene]bis(5-chloro-1*H*-indole), **11** in DMSO-d$_6$

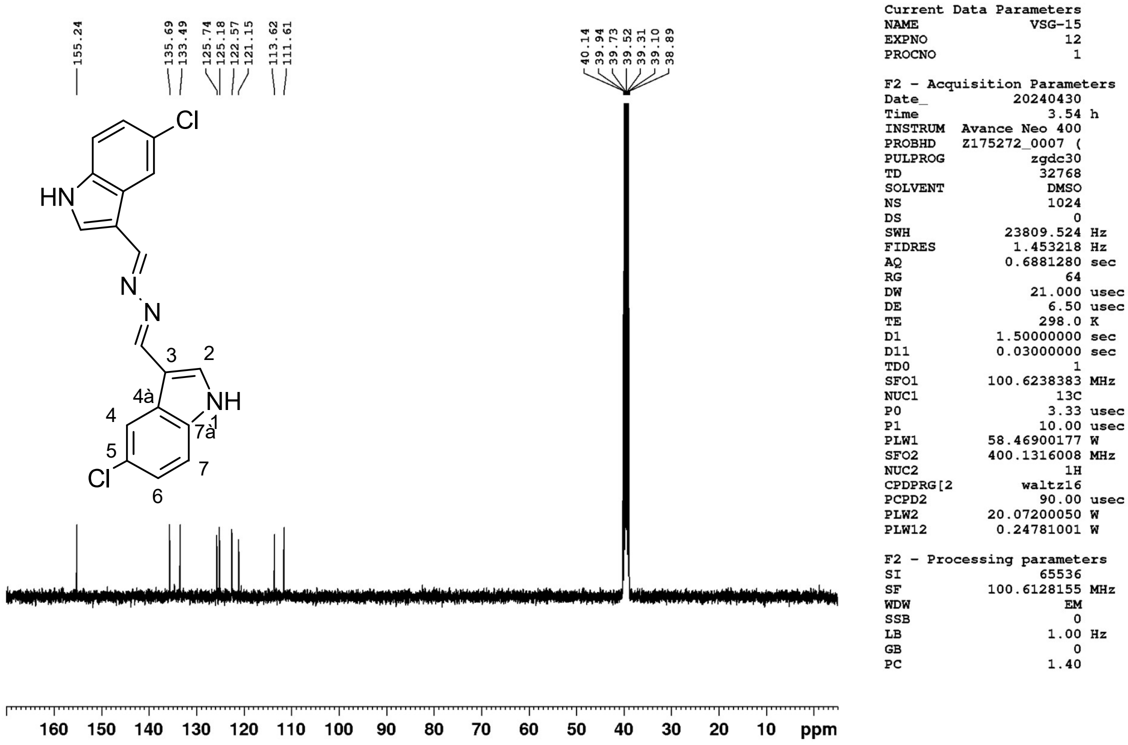

Figure 70S. ^{13}C NMR spectrum of 3,3'-[(1*E*,2*E*)-hydrazinediylidenedi-(*E*)-methanylylidene]bis(5-chloro-1*H*-indole), **11** in DMSO-*d$_6$*

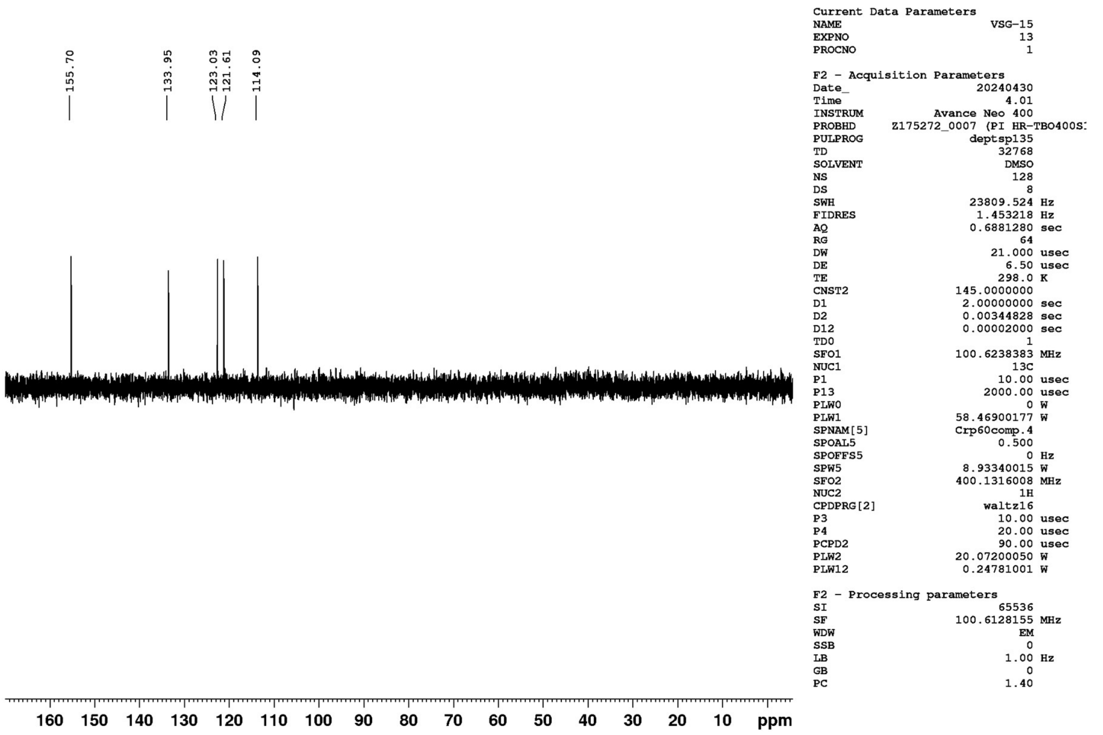

Figure 71S. DEPT-135 NMR spectrum of 3,3'-[(1*E*,2*E*)-hydrazinediylidenedi-(*E*)-methanylylidene]bis(5-chloro-1*H*-indole), **11** in DMSO-d_6

h:\lc-ms\2024\03\27\vs\vs_240327_09 03/27/24 16:51:36

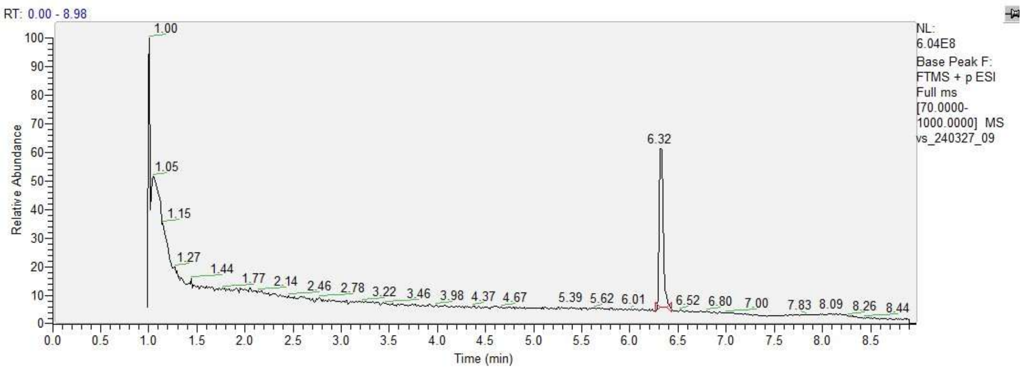

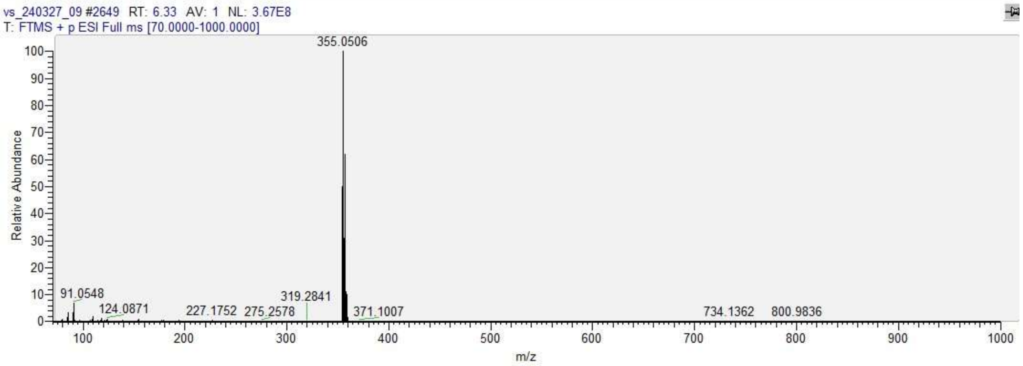

vs_240327_09#2643-2673 RT: 6.32-6.38 AV: 6
T: FTMS + p ESI Full ms [70.0000-1000.0000]
m/z= 342.5378-367.8248

LC-MS

[M+H]+

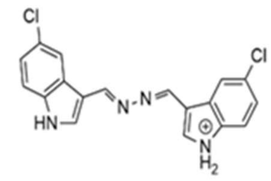

Chemical Formula: $C_{18}H_{13}Cl_2N_4^+$
Exact Mass: 355.05118

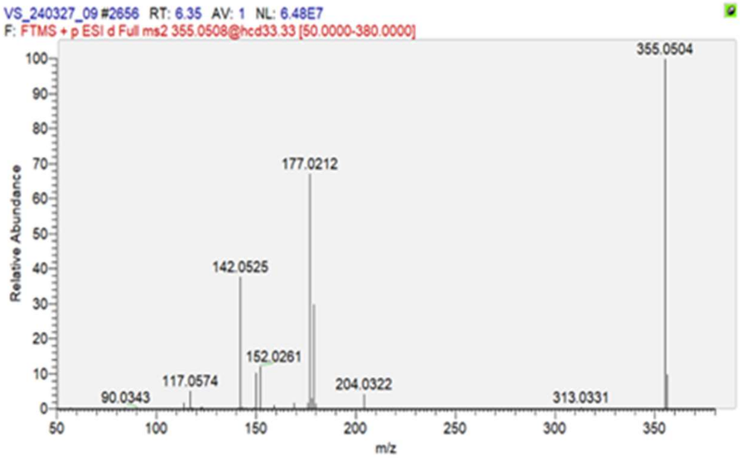

Fragments

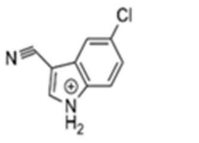

Chemical Formula: $C_9H_6ClN_2^+$
Exact Mass: 177.02140

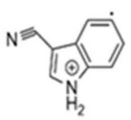

Chemical Formula: $C_9H_6N_2^{\bullet+}$
Exact Mass: 142.05255

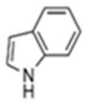

Chemical Formula: C_8H_7N
Exact Mass: 117.05785

Figure 72S. HRMS of 3,3'-[(1*E*,2*E*)-hydrazinediylidenedi-(*E*)-methanylylidene]bis(5-chloro-1*H*-indole), **11**

Table S1. Binary coded structures of the indolyl-methylidene substituted phenylsulfonylhydrazones used in the QSAR study.

Substituents	Ar							R				LE MCF	LE MDA	SI MCF	SI MDA	Reference
id	4-H	4-CH_3	4-OCH_3	2,4,6-CH_3	2,4-CH_3	3,4-CH_3	5-Cl-ind	1-$COCH_3$	5-OCH_3	5-Br	5-Cl					
**1a**	1	0	0	0	0	0	0	0	1	0	0	0.271	0.240	8.667	1.677	48
**1b**	0	1	0	0	0	0	0	0	1	0	0	0.254	0.211	5.000	0.471	48
**1c**	1	0	0	0	0	0	0	1	0	0	0	0.252	0.197	40.222	1.885	48
**1d**	0	1	0	0	0	0	0	1	0	0	0	0.23	0.167	46.000	1.272	48
**1e**	1	0	0	0	0	0	0	0	0	0	1	0.286	0.275	13.000	7.222	48
**1**	0	0	1	0	0	0	0	1	0	0	0	0.206	0.181	13.488	2.886	This study
**2**	0	0	0	1	0	0	0	1	0	0	0	0.200	0.177	20.975	5.147	This study
**3**	0	0	1	0	0	0	0	0	0	1	0	0.220	0.198	3.577	1.051	This study
**4**	0	0	0	1	0	0	0	0	0	1	0	0.215	0.188	5.000	1.055	This study
**5**	0	1	0	0	0	0	0	0	0	1	0	0.197	0.206	1.176	1.861	This study
**6**	1	0	0	0	0	0	0	0	0	1	0	0.238	0.242	1.847	2.319	This study
**7**	0	0	1	0	0	0	0	0	0	0	1	0.204	0.201	2.063	1.782	This study
**8**	0	0	0	1	0	0	0	0	0	0	1	0.193	0.190	1.579	1.326	This study
**9**	0	0	0	0	1	0	0	0	0	0	1	0.204	0.209	1.282	1.674	This study
**10**	0	0	0	0	0	1	0	0	0	0	1	0.206	0.203	3.087	2.669	This study
**11**	0	0	0	0	0	0	1	0	0	0	1	0.195	0.174	2.509	0.814	This study
